# Impaired oxygen-sensitive regulation of mitochondrial biogenesis within the von Hippel–Lindau syndrome

**DOI:** 10.1038/s42255-022-00593-x

**Published:** 2022-06-27

**Authors:** Shuijie Li, Wenyu Li, Juan Yuan, Petra Bullova, Jieyu Wu, Xuepei Zhang, Yong Liu, Monika Plescher, Javier Rodriguez, Oscar C. Bedoya-Reina, Paulo R. Jannig, Paula Valente-Silva, Meng Yu, Marie Arsenian Henriksson, Roman A. Zubarev, Anna Smed-Sörensen, Carolyn K. Suzuki, Jorge L. Ruas, Johan Holmberg, Catharina Larsson, C. Christofer Juhlin, Alex von Kriegsheim, Yihai Cao, Susanne Schlisio

**Affiliations:** 1 Department of Microbiology, Tumor and Cell Biology, Karolinska Institutet, Stockholm, Sweden; 2 College of Pharmacy, Harbin Medical University, Harbin, China; 3 Department of Medical Biochemistry and Biophysics, Karolinska Institute, Stockholm, Sweden; 4 Edinburgh Cancer Research Centre, IGMM, University of Edinburgh, Edinburgh, UK; 5 Department of Physiology and Pharmacology, Karolinska Institutet, Stockholm, Sweden; 6 Department of Medicine, Karolinska University Hospital, Stockholm, Sweden; 7 Department of Microbiology, Biochemistry and Molecular Genetics, Rutgers University-New Jersey Medical School, Newark, NJ USA; 8 Department of Molecular Biology, Faculty of Medicine, Umeå University, Umeå, Sweden; 9 Department of Oncology-Pathology, Karolinska Institutet, Karolinska University Hospital, Stockholm, Sweden

**Keywords:** Cancer metabolism, Metabolism, Tumour-suppressor proteins, Mitochondria

## Abstract

Mitochondria are the main consumers of oxygen within the cell. How mitochondria sense oxygen levels remains unknown. Here we show an oxygen-sensitive regulation of TFAM, an activator of mitochondrial transcription and replication, whose alteration is linked to tumours arising in the von Hippel–Lindau syndrome. TFAM is hydroxylated by EGLN3 and subsequently bound by the von Hippel–Lindau tumour-suppressor protein, which stabilizes TFAM by preventing mitochondrial proteolysis. Cells lacking wild-type *VHL* or in which EGLN3 is inactivated have reduced mitochondrial mass. Tumorigenic *VHL* variants leading to different clinical manifestations fail to bind hydroxylated TFAM. In contrast, cells harbouring the Chuvash polycythaemia *VHL*^*R200W*^ mutation, involved in hypoxia-sensing disorders without tumour development, are capable of binding hydroxylated TFAM. Accordingly, VHL-related tumours, such as pheochromocytoma and renal cell carcinoma cells, display low mitochondrial content, suggesting that impaired mitochondrial biogenesis is linked to VHL tumorigenesis. Finally, inhibiting proteolysis by targeting LONP1 increases mitochondrial content in VHL-deficient cells and sensitizes therapy-resistant tumours to sorafenib treatment. Our results offer pharmacological avenues to sensitize therapy-resistant VHL tumours by focusing on the mitochondria.

## Abstract (web summary)

Li et al. show how mitochondrial content is regulated when oxygen is depleted, as well as how this process is impaired in cancer types related to the von Hippel–Lindau syndrome.

## Main

Hypoxia-inducible transcription factor-α (HIFα) functions as a key regulator of cellular and systemic homeostatic responses to hypoxia. The process orchestrating the oxygen-sensitive regulation of the HIFs is regulated by the oxygen-dependent activity of the prolyl hydroxylase enzymes (EGLN). Prolyl hydroxylation of HIFα allows substrate recognition by the von Hippel–Lindau tumour-suppressor protein (pVHL) causing HIFα ubiquitination and degradation under normal oxygen concentrations^1–3^. Although mitochondria are the major consumers of oxygen in the cell, mitochondrial biogenesis has not been reported to be directly regulated by HIFα. However, HIF1α has been reported to potentially inhibit mitochondrial biogenesis indirectly by repression of c-MYC activity^4^.

Von Hippel–Lindau (VHL) disease is a hereditary cancer syndrome caused by mutations of the *VHL* gene resulting in different tumour subtypes including haemangioblastoma (HB) of the retina and the nervous system, clear cell renal cell carcinoma (ccRCC) and pheochromocytoma and paraganglioma (PPGL)^5^. HIF2α deregulation plays an important role in VHL-defective tumours; however, HIF2α mutations have only been observed in some sporadic cases of PPGL and have not been observed in ccRCC^6–8^. Moreover, the discovery of the oxygen-sensitive regulation of HIFα by pVHL cannot explain the mechanisms underlying the complex genotype–phenotype correlations in VHL syndrome. Type 1 VHL disease is defined as ccRCC and HB with low risk of PPGL and caused by truncating or missense *VHL* mutations. In contrast, type 2 VHL disease is associated with *VHL*-missense mutations and defined by PPGL, either alone (type 2C) or in combination with HB (type 2A) or with HB and ccRCCs (type 2B). Importantly, some germline type 2C *VHL* mutants in familial PPGL retain the ability to suppress HIFα^9,10^. Therefore, VHL’s canonical substrate, HIFα, cannot fully explain the complex genotype–phenotype manifestation within the VHL syndrome and there is no evidence that HIFα deregulation is sufficient to cause cancer^11^. Instead, a number of other VHL functions independent of HIFα regulation have been ascribed to pVHL, including binding to fibronectin, collagen, atypical PKC, SFMBT1, TBK1, ZHX2 and AKT^12–19^. Previously, we also described a new VHL target, BIM-EL, that links type 2C *VHL* mutations to PPGL independent of HIFα regulation^20^.

Another puzzling phenotype of *VHL* germline mutations has been described in individuals from the Chuvash region who are homozygotes for the *VHL*^*R200W*^ mutation^21^. Whereas germline *VHL* mutations commonly predispose individuals to the development of multiple tumours, homozygous carriers of germline *VHL*^*R200W*^ mutations show total absence of tumour development despite increased HIFα signalling^22–24^. These individuals present with a congenital erythrocytosis (excess of red blood cell production) named Chuvash polycythaemia^21^. The absence of tumour development in people with Chuvash polycythaemia suggests that deregulation of HIFα may not be sufficient to drive tumorigenesis in the VHL cancer syndrome and that VHL has other substrates that are required for tumour suppression.

Here we identified an oxygen-sensitive function of pVHL regulating mitochondrial biogenesis independent of the canonical substrate HIFα, that is defective in all VHL cancer syndrome mutations we tested, but normal in the *VHL*^*R200W*^ Chuvash mutation. Mitochondrial transcription factor A (TFAM), a key activator of mitochondrial transcription and replication, is hydroxylated by the oxygen-sensitive hydroxylase EGLN3 on proline 53/66 and subsequently bound and stabilized by pVHL. VHL-related tumours such as PPGL and ccRCC show low mitochondrial content, implicating that lack of mitochondrial content is related to malignancies of tumorigenesis in the VHL syndrome.

## Results

### Mitochondrial content is regulated by pVHL

**Fig. 1:**
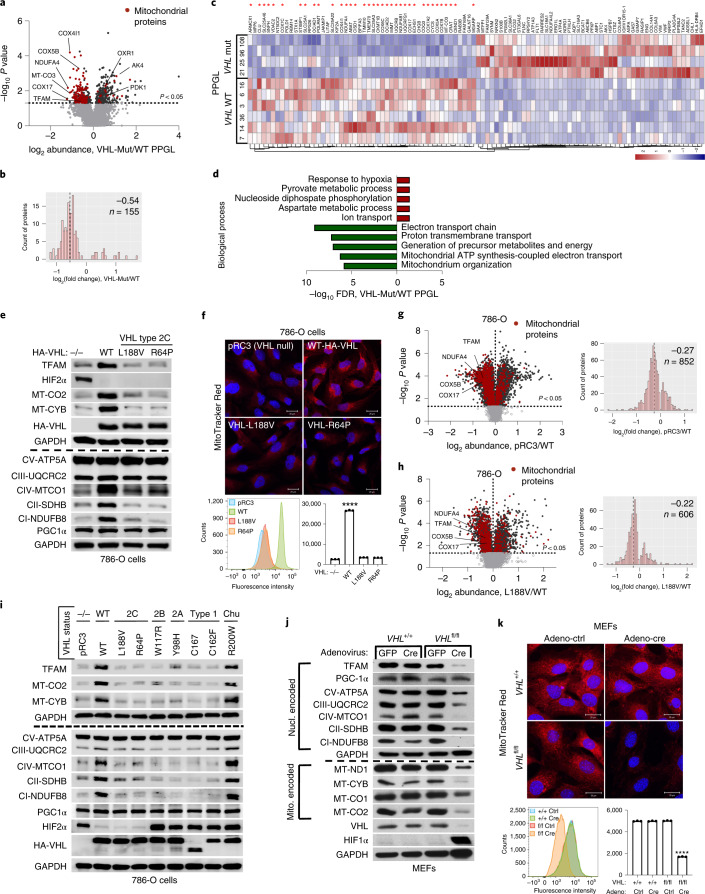
pVHL regulates of mitochondrial mass independent of HIFα. **a**, Volcano plot of proteins detected by nanoLC–MS/MS in human PPGL tumours (*VHL*-mutant/wild type). The dotted line indicates a *P* value of 0.05 (−log_10_ *P* value = 1.3) in two-tailed unpaired *t*-test. **b**, Histogram of mitochondrial proteins regulated in human *VHL*-mutant compared to *VHL* wild-type PPGL tumours. **c**, Heat map of the top 50 downregulated and upregulated proteins in human VHL-related PPGL tumours (VHL-mutant/wild type). Red asterisks indicate mitochondrial proteins. **d**, Top 5 biological processes of the top 50 upregulated (red) and downregulated (green) proteins for human VHL PPGL tumours. **e**, Immunoblot of 786-O cells expressing pVHL WT or type 2C pVHL mutants (p.Leu188Val or p.Arg64Pro). *n* = 3 biological independent experiments. **f**, The corresponding immunofluorescence images are shown. Cells were stained by MitoTracker Red (top). Flow cytometry analysis of MitoTracker Green-stained cells (bottom). Data are presented as mean values ± s.d. One-way analysis of variance (ANOVA) Tukey’s multiple-comparison test. ****P < 0.0001. *n* = 3 biological independent experiments. **g**, The volcano plot shows proteins detected by nanoLC–MS/MS in human ccRCC cells (786-O). *VHL*-null cells (pRC3) were compared to *VHL*-WT-expressing cells. The dotted line indicates a *P* value of 0.05 (−log_10_ *P* value = 1.3) in a two-tailed unpaired *t*-test. The histogram shows fold changes of mitochondrial proteins comparing pRC3 to *VHL*-WT with indicated median value of log_2_(fold change) −0.27. **h**, The volcano plot shows proteins detected by nanoLC–MS/MS in 786-O cells. *VHL*-L188V mutant cells were compared to *VHL*-WT-expressing cells as in **g**. The histogram shows fold changes of mitochondrial proteins comparing *VHL*-L188V to *VHL*-WT with indicated median value of log_2_(fold change) of −0.22. **i**, Immunoblot of 786-O cells stably transfected to produce the indicated pVHL species. **j**, Immunoblot of *VHL* MEFs with indicated genotype. In **i** and **j**, *n* = 3 biological independent experiments. **k**, Corresponding immunofluorescence of *VHL* MEFs. Cells were stained by MitoTracker Red to visualize mitochondria (top). Flow cytometry analysis of MitoTracker Green-stained MEFs (bottom). *n* = 3 biological independent experiments. Data are presented as mean values ± s.d. One-way ANOVA Tukey’s multiple-comparison test. ****P < 0.0001. Source data

**Extended Data Fig. 1:**
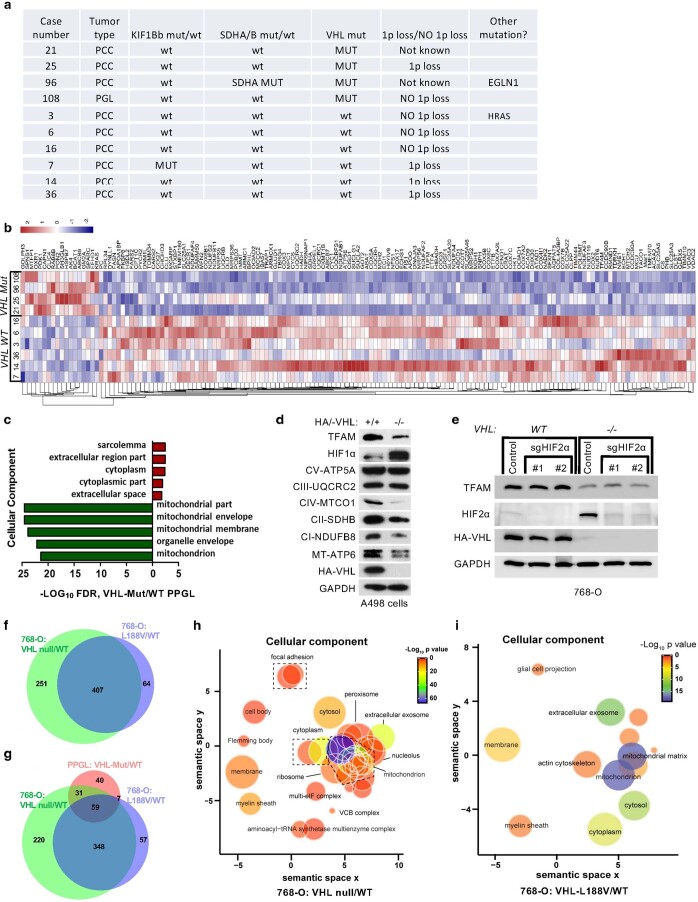
VHL regulates of mitochondrial mass independent of HIFa. (a) List human primary PPGL tumors with characterized mutation status and 1p36 status that were analyzed by by nanoLC-MS/MS in Fig. 1A-D. wt = wild-type. (b) Heatmap of significantly regulated mitochondrial proteins in VHL-mutant compared to VHL wild-type PPGL tumors (p < 0.05, two-tailed unpaired t test). (c) Top 5 cellular component of top 50 up (red)- and down (green)-regulated proteins for human VHL mutant PCC/PGL tumors compared to VHL wild type PCC/PGL tumors according to the false discovery rate (FDR). Medium confidence threshold (0.4) was used to define protein-protein interactions. (d) Immunoblot analysis of A498 VHL-null cells (−/−) stably transfected to generate HA-VHL (WT). n = 3 biological independent experiments. (e) Immunoblot analysis of 786-O cells with indicated genotype stably transduced with lentivirus encoding sgRNA targeting HIF2α. n = 3 biological independent experiments. (f) Venn diagram representing significantly downregulated proteins shared in VHL-null 786-O cells with type 2 C VHL-L188V mutant cells and (g) shared with VHL mutant PPGL. (h, i) GO term enrichment in cellular component of 393 significantly down-regulated proteins (p values < 0.0001, two-tailed unpaired t test) comparing VHL-null to VHL-WT cells (h) and 200 significantly down-regulated proteins (p values < 0.0001, two-tailed unpaired t test) comparing VHL-L188V to VHL-WT cells (i) performed using DAVID and plotted using REVIGO. The size of the bubbles is indicative of the number of proteins annotated with that GO term; bubbles are color coded according to significance. Source data

Germline type 2C *VHL* mutations predisposing to PPGL retain the ability to suppress HIFα^9,10^. To identify the pVHL functions independent of its canonical substrate HIFα, we performed comparative proteomics of PPGL (*n* = 10) with wild-type or mutated *VHL* (Extended Data Fig. 1a). The cellular proteomes from primary PPGL tumours were extracted and analysed by nanoscale liquid chromatography coupled to tandem mass spectrometry (nanoLC–MS/MS). A total of 6,196 proteins were identified and quantified, 5,576 of which were common to all the samples (Supplementary Table 1). To investigate the effect of *VHL* mutations, we combined the proteomes of all the *VHL* wild-type PPGL samples and compared it with the *VHL*-mutant proteome (Fig. 1a). We observed a significantly larger percentage of mitochondrial proteins downregulated in *VHL*-mutant samples as compared to wild-type PPGL samples (Fig. 1a,b and Extended Data Fig. 1b; uncorrected *P* value = 7.95 × 10^−35^, Fisher exact test). Among the significantly differentially expressed proteins, 36 of the top 50 (that is, 72%) that were downregulated in *VHL*-mutant PPGL samples were mitochondrial proteins including the mitochondrial-encoded protein MT-CO3 (Fig. 1c), implicating that mitochondrial proteomes differ between *VHL*-mutant and wild-type PPGL. Furthermore, Gene Ontology (GO) term enrichment was tested among the top 50 significantly upregulated and downregulated proteins (Fig. 1d; *P* < 0.05, two-tailed unpaired *t*-test). Response to hypoxia and pyruvate metabolism were found as the most significantly enriched biological processes for the upregulated proteins, while downregulated proteins related to electron transport and mitochondrial content were overrepresented in biological processes and cellular components according to false discovery rate values in STRING results (Fig. 1d and Extended Data Fig. 1c).

HIFα activation has been reported to be sufficient for many of the manifestations of *VHL* loss^1–3^ and HIFα activation has also been reported to inhibit mitochondrial biogenesis^4^. To understand if the downregulation of mitochondrial proteins in *VHL*-mutant PPGL is dependent on HIFα, we tested type 2C *VHL* mutants that predispose to PPGL without grossly deregulating HIFα^9^. Compared to wild-type *VHL*, the type 2C *VHL* mutants (*VHL*^*L188V*^ and *VHL*^*R64P*^) were clearly defective with respect to restoring abundance of mitochondrial proteins despite their ability to repress HIF2α (Fig. 1e). In particular, PGC1α, a key transcriptional co-activator regulating mitochondrial biogenesis^25^ was unaffected. To exclude potential effects of HIF2α regulating TFAM expression, we depleted *EPAS1* (HIF2α) in 786-O cells (Extended Data Fig. 1e). *EPAS1* loss in cells expressing wild-type pVHL (*VHL*-WT) or *VHL*-null cells had no effect on TFAM protein expression. Similarly to 786-O cells, wild-type *VHL* restored abundance of mitochondrial proteins in another ccRCC cell line, A498 (Extended Data Fig. 1d).

In addition, mitochondrial stainings of 786-O cells using MitoTracker combined with flow cytometry analysis confirmed that mitochondrial fluorescence intensity is restored in *VHL* wild-type cells, but not in type 2C *VHL*-mutant cells (Fig. 1f). Analysing the proteome of *VHL*-null and *VHL*^*L188V*^ cells confirmed that the percentage of mitochondrial proteins was significantly lower in both *VHL*-null cells (uncorrected *P* value = 4.51 × 10^−44^, Fisher exact test) as well as in type 2 *VHL*-mutant cells (uncorrected *P* value = 2.94 × 10^−21^, Fisher exact test) as compared to *VHL* wild-type-expressing cells (Fig. 1g,h, Extended Data Fig. 1h,i and Supplementary Table 2) Importantly, the majority of significant downregulated mitochondrial proteins in *VHL*-null cells (total of 656) was shared with the mitochondrial proteome in type 2C *VHL*^*L188V*^ cells (407 shared mitochondrial proteins; Extended Data Fig. 1f), suggesting a HIFα-independent function of pVHL. Indeed, the percentage of significantly downregulated mitochondrial proteins that was shared between *VHL*^*L188V*^ and *VHL*-null cells was significantly higher than for significantly downregulated non-mitochondrial proteins (odds ratio = 1.26, uncorrected *P* value = 0.0098, Fisher exact test). Additionally, the percentage of downregulated mitochondrial proteins shared between *VHL* mutated PPGL and *VHL*^*L188V*^ cells (Extended Data Fig. 1g) was also significantly higher than for significantly downregulated non-mitochondrial proteins (odds ratio = 3.27, uncorrected *P* value = 4.64 × 10^−5^, Fisher exact test), indicating HIFα-independent function of pVHL.

Furthermore, it has been previously reported that primary ccRCC cells display minimal mitochondrial respiratory capacity and low mitochondrial number^26^. Thus, we asked if other VHL type 1 (low risk PPGL), type 2A (low risk ccRCC) or type 2B (high risk ccRCC) mutants present similarly low mitochondrial contents as those observed in the type 2C *VHL* mutants. Only the reintroduction of wild-type *VHL* but none of the type 1, 2A, 2B or 2C mutations tested could restore the expression of mitochondrial proteins (Fig. 1i). In addition, we confirmed low mitochondrial content in a genetically defined system, using Cre-mediated deletion of *VHL* in mouse embryonic fibroblasts (MEFs) homozygous for a floxed *VHL* allele (Fig. 1j,k).

### Regulation of mitochondrial mass is hydroxylation dependent

**Fig. 2:**
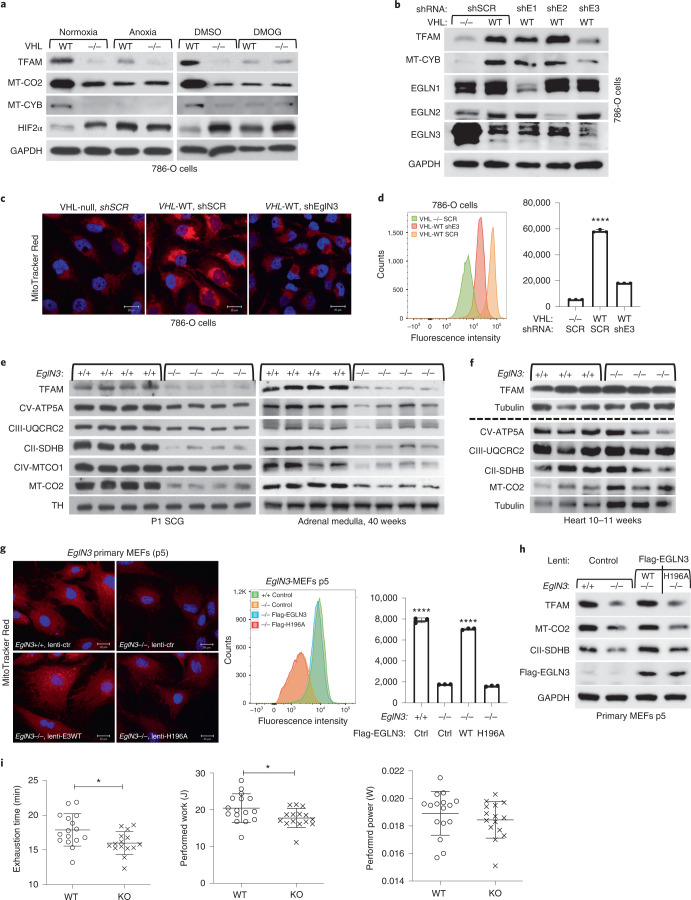
pVHL regulation of mitochondrial mass is hydroxylation and EGLN3 dependent. **a**, Immunoblot analysis of 786-O cells with indicated genotype upon anoxic condition for 16 h or treated with 1 mM DMOG for 8 h. *n* = 3 biological independent experiments. **b**, Immunoblot analysis of 786-O cells with indicated VHL status transduced with lentiviral pL.KO shRNA targeting *EGLN1* (shE1), *EGLN2* (shE2), *EGLN3* (shE3) or no targeting control (shSCR). *n* = 3 biological independent experiments. **c**, Fluorescence images of 786-O cells with indicated VHL status transduced with lentiviral pL.KO shRNA targeting *EGLN3* or no targeting control. Mitochondria are visualized by MitoTracker Red staining. **d**, Corresponding flow cytometry analysis of MitoTracker Green-stained 786-O cells. Data are presented as mean values ± s.d. One-way ANOVA Tukey’s multiple-comparison Test. ****P < 0.0001. *n* = 3 biological independent experiments. **e**, Immunoblot analysis of mouse SCG and adrenal medulla of indicated genotypes. *n* = 4 biologically independent *EGLN3* wild-type or knockout mice. **f**, Immunoblot analysis of mouse heart of the indicated genotypes. *n* = 3 biologically independent *EGLN3* wild-type or knockout mice. **g**, Fluorescence images of primary *EGLN3* MEFs of the indicated genotypes stably transduced with lentivirus encoding *EGLN3* WT, catalytic death mutant *EGLN3*-H196A or empty control. Mitochondria were visualized by MitoTracker Red staining. Corresponding flow cytometry analysis of MitoTracker Green-stained primary MEFs cells of indicated genotype stably transduced with lentivirus encoding *EGLN3* WT, catalytic death mutant *EGLN3*-H196A or empty control. *n* = 3 biological independent experiments. Data are presented as mean values ± s.d. One-way ANOVA Tukey’s multiple-comparison test. ****P < 0.0001. **h**, Corresponding immunoblot of primary *EGLN3* MEFs. *n* = 3 biological independent experiments. **i**, KO mice aged 56–60 weeks reached exhaustion significantly earlier and performed less work at a comparable performed power (WT *n* = 16, KO *n* = 15 independent biological samples per genotype, male mice). Data are presented as mean values ± s.d. Two-tailed unpaired *t*-test. *P* = 0.014, *P* = 0.0318. Source data

**Extended Data Fig. 2:**
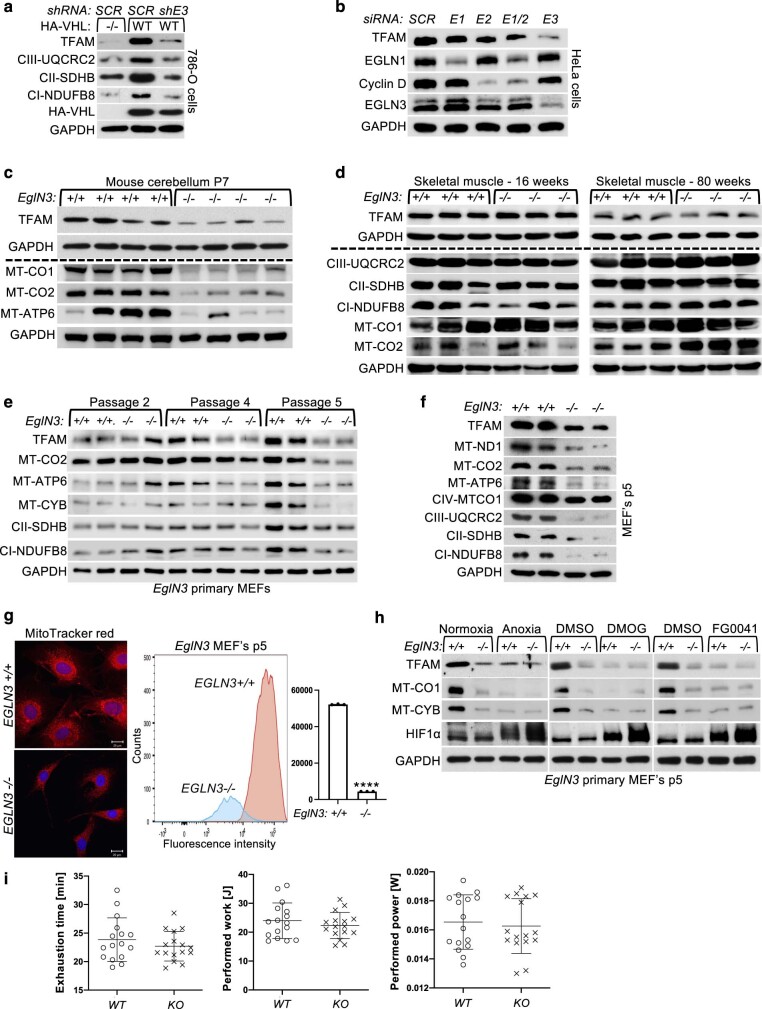
VHL regulation of mitochondrial mass is hydroxylation and EglN3 dependent. (a) Immunoblot analysis of 786-O cells with indicated VHL status transduced with lentiviral pL.KO shRNA targeting EGLN3 (shE3) or no targeting control (SCR). n = 3 biological independent experiments. (b) Immunoblot analysis of HeLa cells transduced with lentiviral pL.KO shRNA targeting EGLN1, EGLN2, EGLN3 or no targeting control. n = 3 biological independent experiments. (c) Immunoblot analysis of mouse cerebellum of indicated genotype. n = 4 biologically independent EGLN3 wildtype or knockout mice. (d) Immunoblot analysis of mouse skeletal muscles of indicated genotype. n = 3 biologically independent EGLN3 wildtype or knockout mice. (e) Immunoblot of primary EglN3-MEFs of indicated genotype with different passages. n = 3 biological independent experiments. (f) Immunoblot analysis of primary EGLN3-MEFs of indicated genotype. n = 3 biological independent experiments. (g) Left: Fluorescence images of primary EGLN3-MEFs of indicated genotype. Mitochondria were stained by MitoTracker Red. Right: Flow cytometry analysis of MitoTracker Green-stained primary MEFs of indicated genotype. Data are presented as mean values ± S.D. Two-tailed unpaired t test. ****p < 0.0001. n = 3 biological independent experiments. (h) Immunoblot of EGLN3 primary MEFs with indicated genotype upon normoxic or anoxic conditions for 16 h or treated with 1 mM DMOG or 50 μM FG0041 for 8 h. n = 3 biological independent experiments. (i) In contrast young adult, KO mice (18-19 weeks of age) show a comparable exhaustion time, performed work and performed power (n = 16 per genotype, male mice). Data represent means ± SD and individual measurements. Source data

pVHL has previously been shown to bind hydroxylated prolines of other substrates besides HIFα, such as AKT, BIM-EL, ZHX2 and pTBK1 (refs. ^16,19,20,27^). To determine if regulation of mitochondrial content by pVHL is mediated by proline hydroxylation, we cultured 786-O cells expressing exogenous HA-VHL under anoxia (0.1% oxygen) or with hydoxylase inhibitor dimethyloxalylglycine (DMOG) and found that mitochondrial protein levels were decreased, similar to those of *VHL*-null cells under these conditions (Fig. 2a). To investigate the prolyl hydroxylase that may contribute to the regulation of mitochondrial proteins by pVHL, we silenced the three EGLN family members in HeLa and 786-O cells expressing *VHL* and found that EGLN3 is the primary prolyl hydroxylase that showed the most robust decrease of mitochondrial proteins (Fig. 2b and Extended Data Fig. 2a,b). Consistent with these results, MitoTracker stainings of mitochondria and corresponding flow cytometry analysis confirmed significantly low mitochondrial content in *VHL-*expressing cells in which *EGLN3* was downregulated with shRNA (Fig. 2c,d). To understand the impact of mitochondrial content by loss of *EGLN3* in vivo, we analysed tissues from *EGLN3*^−/−^ knockout (KO) mice. Mitochondrial proteins in *EGLN3*^*−/−*^ mouse superior cervical ganglia (P1 SCG), adult adrenal medulla and cerebellum (P7) were remarkably reduced (Fig. 2e and Extended Data Fig. 2c). However, other tissues such as heart and skeletal muscle did not show any changes in mitochondrial protein content (Fig. 2f and Extended Data Fig. 2d). It is possible that degradation of mitochondrial proteins by mitochondrial protease LONP1 might contribute to these tissue-specific differences. Low LONP1 protein expression in skeletal muscle and heart is demonstrated in the Human Protein Atlas (https://www.proteinatlas.org/ENSG00000196365-LONP1/tissue/) and thus might contribute to higher mitochondrial content in these tissues independent of EGLN3 expression.

In addition, we detected decreased expression of mitochondrial proteins in *EGLN3*^−/−^ MEFs when cultured more than five passages (p5), but not in their first or second passage (Extended Data Fig. 2e,f). MitoTracker staining visualizing mitochondria and corresponding flow cytometry analysis confirmed significant low mitochondrial content in *EGLN3*^−/−^ MEFs cultured beyond five passages (Extended Data Fig. 2g). Consistent with our observations in 786-O cells expressing wild-type pVHL (Fig. 2a), *EGLN3* wild-type MEFs (p5) showed low abundance of mitochondrial proteins when cultured under anoxia, or with hydroxylase inhibitors (Extended Data Fig. 2h). To confirm that low mitochondrial content in *EGLN3*^*−/−*^ MEFs (p5) is mediated by EGLN3 hydroxylase activity, we transduced cells with lentivirus encoding WT *EGLN3* or a catalytically dead p.His196Arg *EGLN3* mutant. Mitochondrial content was restored in *EGLN3*^*−/−*^ MEFs transduced with Lenti-EGLN3-WT, but not Lenti-*EGLN3*-p.His196Arg mutant (Fig. 2g,h). Thus, EGLN3 hydroxylation activity regulates mitochondrial content under these conditions. Next, we asked whether changes in mitochondrial content observed in some tissues of *EGLN3*-constitutive KO mice would culminate in an exercise intolerance phenotype, a common feature in settings of decreased mitochondrial biogenesis. Thus, we analysed exercise endurance in younger and older *EGLN3* mice using a treadmill running test. We observed a minor but significant impairment in exercise capacity in older *EGLN3*^*−/−*^ males (56–60 weeks; Fig. 2i), but not in younger males aged 18–19 weeks of age (Extended Data Fig. 2i). Thus, the decreased mitochondrial content observed in certain tissues does not seem to have an impact on the exercise capacity of younger mice, but might play a more indirect role during ageing.

### TFAM hydroxylation on proline 53/66 causes pVHL recognition

**Fig. 3:**
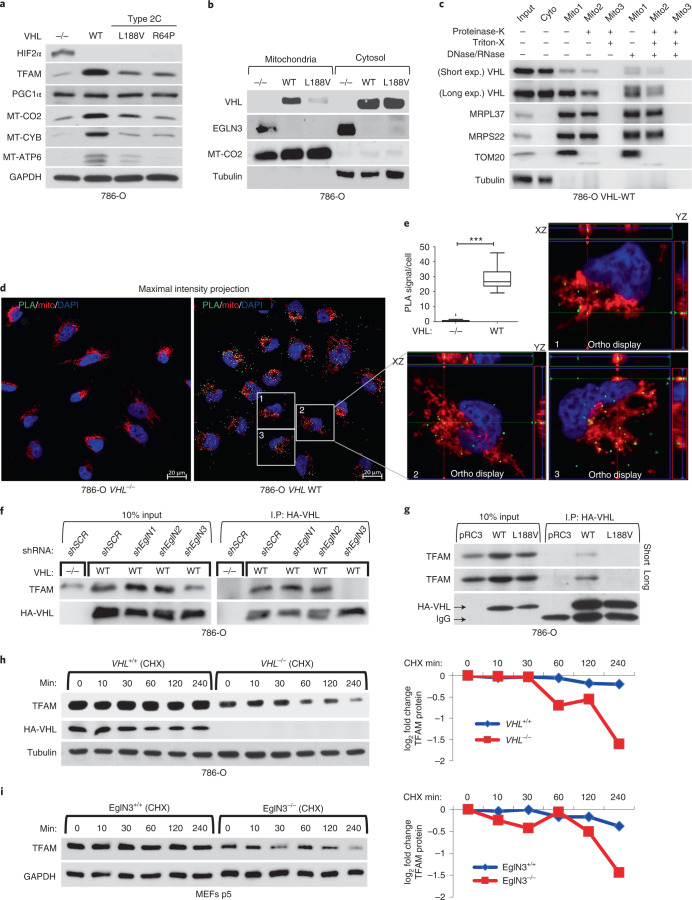
pVHL regulates TFAM protein stability depending on EGLN3 enzymatic activity. **a**, Immunoblot of 786-O cells stably transfected to produce the indicated pVHL species. **b**, Immunoblot analysis of mitochondrial and cytosolic fractions of 786-O stable cells. **c**, Immunoblot analysis of subcellular fractionation of 786-O pVHL WT cells. Mitochondrial fractions were treated with 25 μg ml^−1^ proteinase K with or without 1% Triton X-100. In **a**–**c**, *n* = 3 biological independent experiments. **d**, Representative confocal images of in situ PLA between TFAM and pVHL in 786-O cells with indicated *VHL* status. PLA signal is shown in green, DAPI in blue and MitoTracker in red. Orthogonal views of three cells identified in pVHL-expressing cells are presented and demonstrate colocalization of PLA signal in mitochondria (yellow). Magnification ×63; scale bar, 5 µm. **e**, Quantification of the number of PLA signals per cell in both conditions with indicated *VHL* status; Mann–Whitney *U* test; ***P value < 0.001 (*n* > 200 cells per group examined). Similar results were seen more than three times. The term five-number summary is used to describe a list of five values: the minimum, the 25th percentile, the median, the 75th percentile and the maximum. These are the same values plotted in a box-and-whisker plot when the whiskers extend to the minimum and maximum. **f**, Immunoblots of HA-VHL immunoprecipitation from 786-O cells transduced with lentivirus encoding shRNA targeting *EGLN1*, *EGLN2*, *EGLN3* or scramble control (SCR). **g**, HA-VHL immunoprecipitation from 786-O cells with stable expression of either HA-VHL-WT or HA-VHL-L188V. Immunoblots showing co-immunoprecipitation of endogenous TFAM and HA-VHL. **h**, 786-O *VHL*-null cells or stable HA-VHL-WT-expressing cells were treated with 10 μg ml^−1^ cycloheximide (CHX). At the indicated time points, whole-cell lysates were prepared for immunoblot analysis. Corresponding quantification of the band intensities is shown on the right. **i**, *EGLN3* MEFs with indicated genotype were treated with CHX (10 μg ml^−1^) and whole-cell lysates were prepared for immunoblot analysis at the indicated time points. Corresponding quantification of the band intensities is shown on the right. In **f**–**i**, *n* = 3 biological independent experiments. Source data

**Extended Data Fig. 3:**
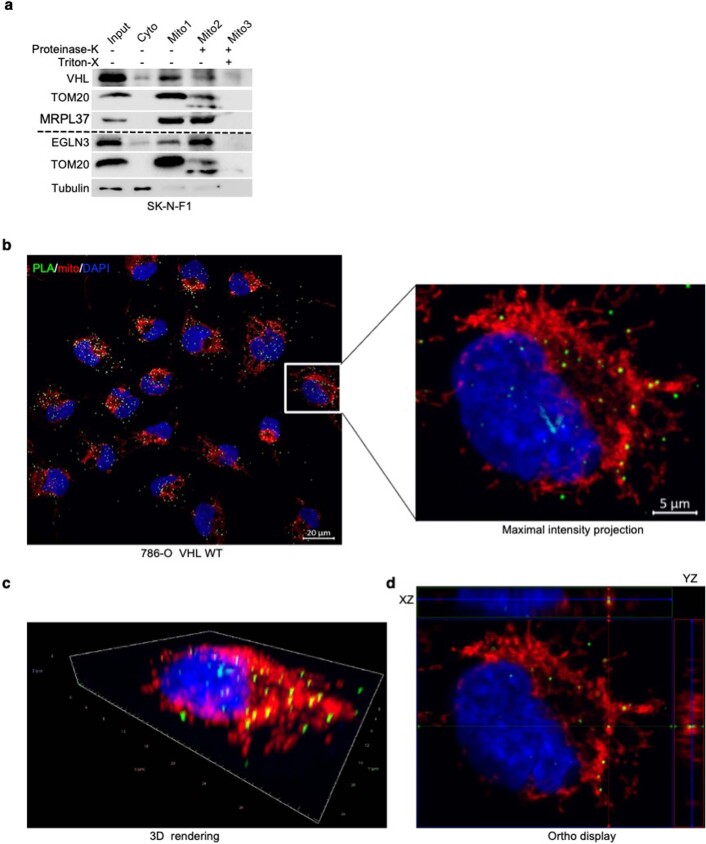
VHL interacts with TFAM within mitochondria. (a) Immunoblot analysis of subcellular fractionation of SK-N-F1 cells. Cell lysates were fractionated into cytosolic and mitochondrial fractions. In addition, aliquots of the mitochondrial fractions were treated with 25 μg/ml Proteinase K with or without treatment with 1% Triton X-100. Fractions were analyzed by western blotting and the localization of VHL or EglN3 was assessed in comparison to that of protein markers of the cytosol (tubulin), outer mitochondrial membrane (TOM20), and mitochondrial matrix (mitochondrial ribosomal protein MRPL37). n = 3 biological independent experiments. (b) Representative images of proximity ligation assay (PLA) signal (green), DAPI (blue) and MitoTracker Red (red) triple staining in 786-0 cells expressing VHL wildtype. The images show the maximal intensity projection of the signal/staining. (c) 3D rendering and (d) Orthogonal view showing co-localization of PLA signal in mitochondria (yellow). Magnification 63x; scale bar: 5 µm. (b-d) Similar results were seen more than three times. Source data

To understand the mechanism of how pVHL can regulate mitochondrial content depending on EGLN3 hydroxylation activity, we investigated regulators of mitochondrial biogenesis, a process by which cells increase their individual mitochondrial mass and copy. Mitochondrial biogenesis is largely coordinated by PGC1α^28^, which in turn regulates the activity of TFAM, a key activator of mitochondrial transcription and mitochondrial genome replication^29^. pVHL and EGLN3 restored TFAM protein abundance in *VHL*-null cells (Fig. 1e,i and Fig. 3a) and in *EGLN3*^−/−^ cells (Fig. 2g,h), respectively, whereas PGC1α protein abundance remained unaffected (Fig. 3a). Because TFAM is localized at the mitochondria, we performed mitochondrial fractionation and found that both, pVHL and EGLN3, partially localized to the mitochondria (Fig. 3b), consistent with previous reports^30^. Interestingly, the *VHL*^*L188V*^ type 2C mutation was barely detected in the mitochondria fraction (Fig. 3b). To validate that pVHL and EGLN3 are localized within the mitochondria, we performed proteinase K digestion to exclude proteins at the mitochondrial outer membrane. This allows the detection of proteins within the inner membrane or matrix only (Fig. 3c and Extended Data Fig. 3a). In *VHL*-expressing 786-O cells, pVHL was detected both in the cytosol, and also within the mitochondria after proteinase K digestion (Fig. 3c). In addition, we could also detect endogenous EGLN3 and pVHL within the mitochondria after proteinase K digestion (Extended Data Fig. 3a).

To investigate direct binding of pVHL with endogenous TFAM within the mitochondria in intact cells, we performed proximity ligation assays (PLAs) combined with mitochondrial staining. We detected PLA fluorescence signal caused by endogenous TFAM binding to pVHL in *VHL*-expressing 786-O cells, but not in *VHL*-null cells (Fig. 3d,e and Extended Data Fig. 3b–d). We visualized PLA fluorescence signal with standard maximum intensity projection and further used spatial resolution with an orthogonal view to the projection axis to validate mitochondrial localization (Fig. 3d,e and Extended Data Fig. 3b–d).

To further explore whether prolyl hydroxylation of TFAM by EGLN3 is responsible for the pVHL-dependent regulation of TFAM abundance, we first investigated if pVHL interacts with endogenous TFAM in 786-O cells expressing HA-VHL. TFAM was readily detected in anti-HA immunoprecipitates of cells expressing HA-VHL unless *EGLN3* (but not *EglN1* or *EglN2)* was downregulated with an effective shRNA (Fig. 3f). Furthermore, TFAM co-immunoprecipitated only with wild-type *VHL*, but not with *VHL*^*L188V*^ type 2C mutant (Fig. 3g). Moreover, when 786-O cells were treated with the protein synthesis inhibitor cycloheximide, the half-life of TFAM was shorter in *VHL*^−/−^ cells compared to wild-type *VHL*-expressing cells (Fig. 3h). Similarly, the half-life of TFAM was shorter in *EGLN3*^−/−^ KO MEFs compared to wild-type MEFs (Fig. 3i), indicating that pVHL and EGLN3 stabilize TFAM protein.

**Fig. 4:**
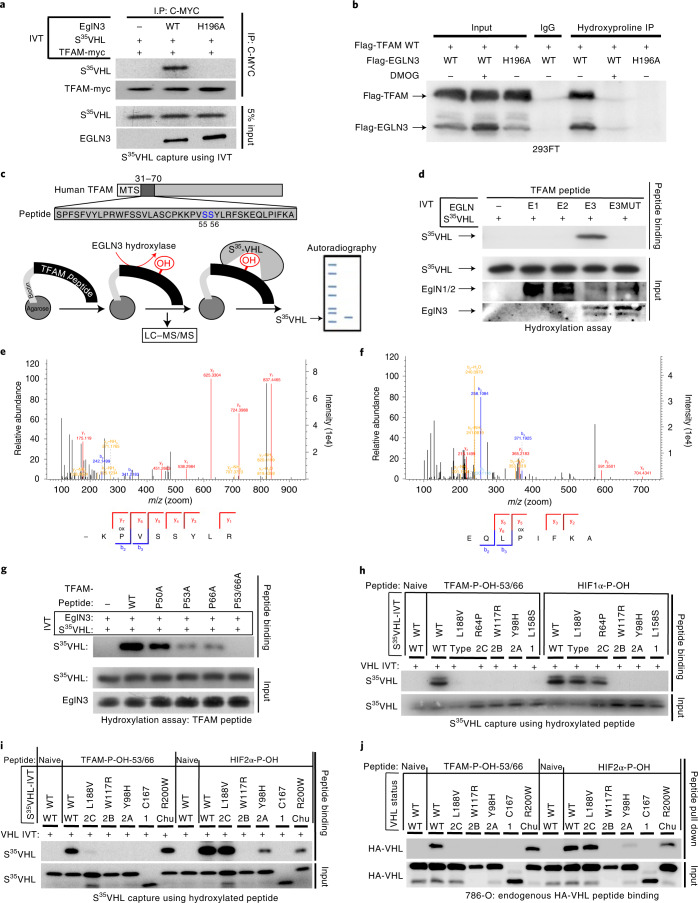
TFAM is hydroxylated by EGLN3 at proline 53/66 causing pVHL recognition. **a**, Autoradiograms showing recovery of ^35^S-labelled VHL protein bound to HA-immunoprecipitated (IP) full-length TFAM that was first subjected before to hydroxylation by EGLN3 WT or *EGLN3*-H196A catalytic mutant. **b**, Immunoprecipitation using anti-hydroxyproline antibody (HydroxyP) from 293FT cells that were transiently transfected with plasmids encoding Flag-TFAM and Flag-EGLN3 WT or catalytically dead mutant (H196A) with or without DMOG treatment. Immunoblots show co-immunoprecipitation of Flag-TFAM and Flag-EGLN3. In **a** and **b**, *n* = 3 biological independent experiments. **c**, Schematic of the hydroxylation assay using the biotinylated synthetic TFAM peptide 31–70. **d**, Autoradiograms showing recovery of ^35^S-labelled VHL protein bound to biotinylated TFAM peptide 31–70. Before pulldown, peptides were incubated with EGLN1, EGLN2, EGLN3 or EGLN3 catalytic mutant (Mut) generated by IVT or unprogrammed reticulocyte lysate (−). Expression of IVT-produced EglN proteins in each reaction was verified by immunoblot. *n* = 3 biological independent experiments. **e**,**f**, Mass spectrometry of biotinylated TFAM peptide 31–70 was subjected to EGLN3 hydroxylation assay. Representative fragmentation spectra of hydroxylated Biotin-KP(ox)VSSYLR (**e**) and hydroxylated Biotin-EQLP(ox)IFKA (**f**). **g**, Autoradiograms of EGLN3 hydroxylation and ^35^S-VHL capture as shown in using biotinylated TFAM peptides containing proline-to-alanine substitutions, or no substitution (WT). **h**, Autoradiograms showing recovery of ^35^S-labelled VHL protein (WT) or corresponding disease mutants (as indicated) bound to biotinylated TFAM peptides synthesized with double hydroxyl-prolines on prolines 53 and 66 (TFAM-P-OH-53/66). Synthetic biotinylated HIF1α peptide (residues 556 to 575) with hydroxylated proline 564 (HIF1α-P-OH) was included as a control. Biotinylated TFAM naïve peptide was used as negative controls. **i**, Autoradiograms showing recovery of ^35^S-labelled VHL protein (WT) or corresponding disease mutants (as indicated) bound to biotinylated TFAM peptides synthesized with double hydroxyl-prolines on prolines 53 and 66 (TFAM-P-OH-53/66). Synthetic biotinylated HIF2α peptide (residues 521 to 543) with hydroxylated proline 531 (HIF2α-P-OH) was included as a control. Biotinylated TFAM and HIF2α naïve peptides were used as negative controls. **j**, Peptide pulldown using biotinylated TFAM-P-OH-53/66 peptide incubated with whole-cell lysates from 786-O cells expressing either HA-VHL-WT or HA-VHL disease mutant. Biotinylated TFAM and HIF2α naïve peptides were used as negative controls. In **g**–**j**, *n* = 3 biological independent experiments. Source data

**Extended Data Fig. 4:**
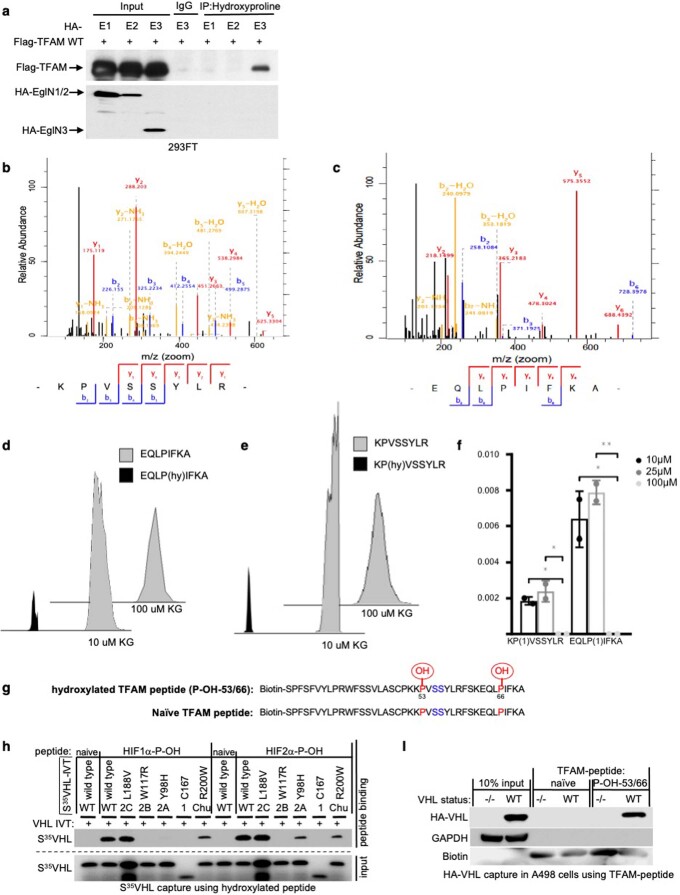
TFAM is hydroxylated by EglN3 at Proline 53/66 causing pVHL recognition. (a) Immunoprecipitation using antihydroxyproline antibody (HydroxyP) from 293FT cells that were transiently transfected with plasmids encoding Flag-TFAM and HA-EGLN1, HA-EGLN2 and HA-EGLN3. Immunoblots show co-immunoprecipitation of Flag-TFAM. n = 3 biological independent experiments. (b-c) Mass spectrometry of unmodified biotinylated TFAM-peptide-30-70. Shown is the representative fragmentation peptide spectra of non-hydroxylated Biotin-KPVSSYLR (b) and non-hydroxylated Biotin-EQLPIFKA (c). (d,e) Extracted ion chromatogram of biotinylated unmodified and mono hydroxylated proline residues 53 (d) or proline residues 66 (e) TFAM peptide following an in vitro hydroxylation reaction with EglN3 with indicated concentration of α-ketoglutarate (KG). Control indicates unmodified biotinylated TFAM-peptide that was not subjected to EGLN3 hydroxylation. (f) Hydroxylation levels of proline residues 53 and 66 of TFAM peptide following hydroxylation with EGLN3 generated via IVT with indicated concentration of KG. Data are presented as mean values ± SD. n = 3 biological experiments. One way ANOVA Tukey’s Multiple Comparison Test. *p < 0.05,**p < 0.01. p = 0.0288, p = 0.0143, p = 0.0148, p = 0.0082. (g) Schematic illustration of synthetic biotinylated TFAM peptide hydroxylated at P-OH-53 and P-OH-66 and naïve TFAM peptide. (h) Autoradiograms showing recovery of 35S-labeled VHL protein (WT) or corresponding disease mutants (as indicated) bound to biotinylated HIF1α peptide (residues 556 to 575) with hydroxylated proline 564 (HIF1α-P-OH) and HIF2α peptide (residues 521 to 543) with hydroxylated proline 531 (HIF2α-P-OH). Biotinylated HIF1α and HIF2α naïve peptides were used as negative controls. n = 3 biological independent experiments. (i) Peptide pulldown using biotinylated TFAM-P-OH-53/66 peptide incubated with whole-cell lysates from A498 cells expressing HA-VHL WT or empty control. Biotinylated TFAM naïve peptide was used as negative control. n = 3 biological independent experiments. Source data

To test whether prolyl hydroxylation is responsible for the *VHL*-dependent regulation of TFAM protein abundance, we investigated if TFAM could be hydroxylated by EGLN3 and subsequently recognized by pVHL. Myc was immunoprecipitated from in vitro translated (IVT) full-length TFAM-myc, which was then used in EGLN3 hydroxylation assays. TFAM-myc captured ^35^S-labelled pVHL after incubation with EGLN3 wild type, but not with a catalytically impaired p.His196Arg *EGLN3* mutant (Fig. 4a). Furthermore, a pan-hydroxyproline antibody immunoprecipitated Flag-TFAM from 293 cells that exogenously expressed EGLN3 unless the EGLN3 was catalytically inactive or the cells were treated with DMOG (Fig. 4b). Hydroxyproline antibody immunoprecipitation of Flag-TFAM was not detected with exogenously expressed EGLN1 or EGLN2 (Extended Data Fig. 4a).

Recent reports demonstrated that phosphorylation within the HMG1 (high mobility group 1) domain of TFAM by protein kinase A (PKA) promotes its degradation by the mitochondrial LONP1 protease^31,32^. Since we observed that EGLN3 is responsible for the pVHL-dependent TFAM stabilization, we generated a 40-amino-acid peptide (TFAM 31–70) spanning the HMG1 domain containing the PKA phosphorylation sites (Fig. 4c) to identify potential hydroxylation residues. Proline hydroxylation was assayed by ^35^S-VHL capture and confirmed by LC–MS/MS analysis (Fig. 4c–f). TFAM peptide 31–70 captured ^35^S-VHL after the EGLN3 hydroxylation reaction, but not after the hydroxylation reaction with the p.His196Arg *EGLN3* catalytically impaired mutant (Fig. 4d). This was a specific function of EGLN3 among the EGLN paralogues (Fig. 4d), consistent with our earlier observations that regulation of TFAM abundance (Fig. 2b and Extended Data Fig. 2b) and hydroxyproline antibody immunoprecipitation of TFAM (Extended Data Fig. 4a) is a distinguishing feature of EGLN3. Hydroxylation of the TFAM peptide 31–70 was confirmed by LC–MS/MS analysis (Fig. 4e,f and Extended Data Fig. 4b–f). MS confirmed that EGLN3 catalyses the hydroxylation of proline 53 and proline 66 (Fig. 4e,f and Extended Data Fig. 4b,c). We detected mono-hydroxylated peptide on either proline 53 or proline 66 (Extended Data Fig. 4d,e). The detected intensity of each proline-hydroxylated peptide was quantified and normalized to the non-hydroxylated peptide (Extended Data Fig. 4f). To understand the importance of mono-hydroxylation or potential di-hydroxylation of the respective proline residues for pVHL binding, we synthesized TFAM peptide 31–70 peptides with the proline-to-alanine substitutions p.Pro50Ala, p.Pro53Ala, p.Pro66Ala and p.Pro53/66Ala, and measured their hydroxylation by EGLN3 using the ^35^S-VHL capture assay (Fig. 4g). The p.Pro50Ala proline substitution did not alter hydroxylation relative to the wild-type peptide. In contrast, the p.Pro53Ala and p.Pro66Ala substitutions significantly impaired ^35^S-VHL capture and the double substitutions p.Pro53/66Ala completely abolished ^35^S-VHL recognition (Fig. 4g). In a reciprocal experiment, we synthesized TFAM peptide 31–70 in which both prolines 53 and 66 (P-OH-53/66) were hydroxylated (Extended Data Fig. 4g). As expected, hydroxylated peptide could, similarly to the hydroxylated HIF1α peptide (556–575), capture ^35^S-VHL (Fig. 4h). In contrast, non-hydroxylated TFAM peptide (naïve) did not capture ^35^S-pVHL. Our finding that TFAM expression can be restored by wild-type *VHL*, but not type 2C *VHL* mutants (Figs. 1e,i and 3a), suggested that the latter cannot recognize hydroxylated TFAM. Indeed, type 2C pVHL mutants bound to hydroxylated HIF1α and HIF2α peptides, but failed to bind hydroxylated TFAM peptide (P-OH-53/66; Fig. 4h,I). Type 1, 2A and 2B pVHL mutants also failed to recognize hydroxylated TFAM (Fig. 4h,i). Next, we tested the *pVHL*^*R200W*^ Chuvash mutation in the ability to bind hydroxylated TFAM. *VHL*^R200W^ has been identified in homozygous carriers with a congenital erythrocytosis (Chuvash polycythaemia) but with a total absence of tumour development^22–24^. In contrast to the VHL cancer syndrome mutations, hydroxylated TFAM peptide captured ^35^S-*VHL*^*R200W*^ mutant similarly to ^35^S-VHL wild type (Fig. 4i). However, HIF1α-P-OH and HIF2α-P-OH peptides were both partially impaired to capture ^35^S-*VHL*^*R200W*^ (Extended Data Fig. 4h), confirming previous reports of partial altered HIFα signalling in Chuvash patients^22–24,33^. Thus, type 1, 2A, 2B and 2C pVHL mutations tested all failed to bind hydroxylated TFAM, regardless of whether they have the ability to bind hydroxylated HIFα or not. In contrast, *pVHL*^*R200W*^ polycythaemia mutation bound hydroxylated TFAM similarly to wild-type VHL. In addition, we noticed that the type 2A pVHL^Y98H^ mutant associated with low risk ccRCC behaved similarly to *pVHL*^*R200W*^ Chuvash mutant being partially, but not fully impaired in HIF2α-P-OH peptide binding, reinforcing the role of HIF2α as oncogenic driver in ccRCC (Extended Data Fig. 4h).

Next, we complemented these studies by expressing wild-type or mutant HA-pVHL in 786-O cells and performing pulldown assays with immobilized TFAM or HIF2α peptide. As expected, wild-type HA-VHL and Chuvash HA-*VHL*^*R200W*^ mutant bound similarly to the dihydroxy-TFAM peptide (P-OH-53/66), but not to any VHL syndrome mutants (Fig. 4j and Extended Data Fig. 4i), confirming the IVT ^35^S-VHL capture assay (Fig. 4i). In contrast to TFAM binding and consistent with the ^35^S-VHL capture assay (Fig. 4i), HA-*VHL*^*R200W*^ capture to hydroxylated HIF2α peptide was partially impaired (Fig. 4j).

In summary, we observed that all tested VHL syndrome mutations failed to recognize hydroxylated TFAM. In contrast, the *VHL*-Chuvash polycythaemia mutant had similar binding affinity as wild-type VHL to hydroxylated TFAM, but was impaired in binding hydroxylated HIFα.

### pVHL binding to hydroxylated TFAM protects from LONP1 degradation

**Fig. 5:**
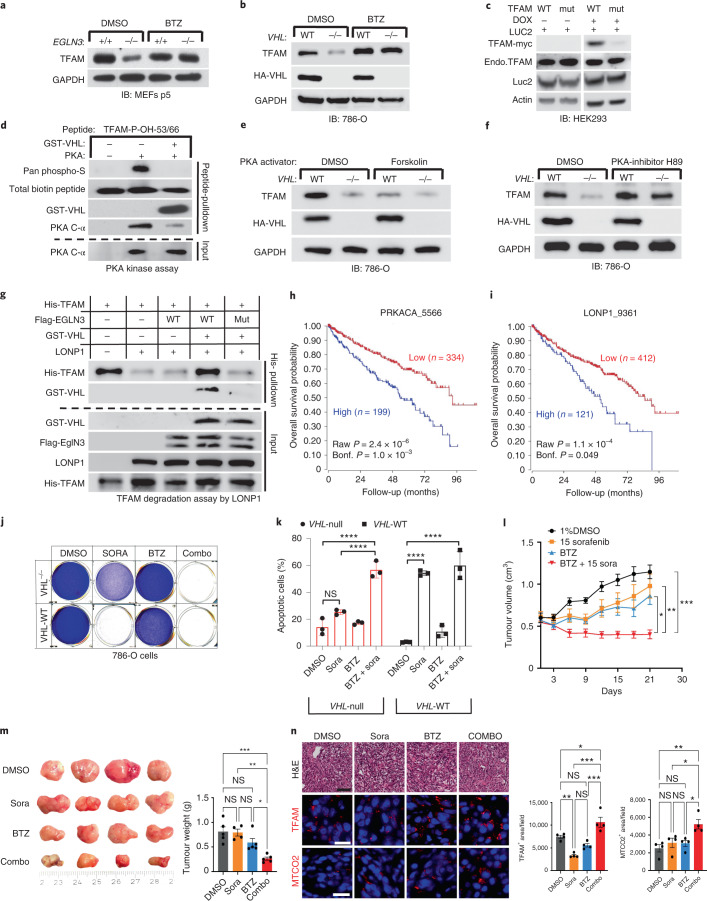
pVHL protects TFAM from LONP1 degradation. **a**,**b**, Immunoblot analysis of primary *Egln3*^+/+^ and *Egln3*^*−/−*^ MEFs (**a**) and 786-O cells (**b**) treated with 1 μM LONP1 inhibitor bortezomib (BTZ) for 16 h. **c**, Immunoblot analysis of HEK293 cells transfected with transposon vectors pB-TRE-TFAM-WT-Luc2, pB-TRE-TFAM-mut-Luc2 and transposase vector pCAG-hyPBase. **d**, Immunoblot analysis of PKA activity assay using biotinylated TFAM peptides with double hydroxyl-prolines 53 and 66. **e**,**f**, Immunoblot analysis of 786-O cells treated with 20 μM PKA activator forskolin (24 h) (**e**) or 5 μM PKA inhibitor H89 (24 h) (**f**). **g**, Immunoblot analysis of TFAM degradation assay by LONP1 using purified His-TFAM, GST-VHL, LONP1 and IVT-synthesized Flag-EGLN3 WT or Flag-EGLN3 catalytic mutant. In **a**–**g**, *n* = 3 biological independent experiments. **h**,**i**, Kaplan–Meier overall survival curve for individuals with high (blue) and low (red) expression of PKA catalytic subunit (PRKACA) (**h**) and LONP1 (**i**) using the Kidney Renal Clear Cell Carcinoma dataset from The Cancer Genome Atlas which contains 533 tumour samples (https://hgserver1.amc.nl/cgi-bin/r2/main.cgi; minimal patient group size of 50 in the iterations). The overall survival probability was estimated with the KaplanScanner tool, using a Bonferroni-corrected logrank test between the two groups of patients. The graph depicts the best *P* value corrected for multiple testing (Bonferroni method). **j**, Crystal violet staining of 786-O cells pretreated with BTZ (10 nM) for 24 h and then treated for 48 h with sorafenib (Sora; 20 μM), BTZ (10 nM) or a combination (combo) of these two drugs as indicated. **k**, Cell apoptosis rate was detected by Annexin V-FITC/propidium iodide (PI) staining using flow cytometry. Data are presented as mean values ± s.d. Two-way ANOVA Tukey’s multiple-comparison test. ****P < 0.0001. *n* = 3 biological independent experiments. **l**, Female athymic NCr^nu/nu^ mice were implanted subcutaneously with 786-O cells. Sorafenib (*n* = 4) or vehicle control (DMSO, *n* = 5) was administered orally, once a day at the dose of 15 mg per kg body weight. BTZ (*n* = 5) was administered by intraperitoneal injection, twice per week at a dose of 1 mg per kg body weight. Combined treatment: 1 mg per kg body weight BTZ + 15 mg per kg body weight sorafenib (*n* = 5). Mean (±s.e.m.) tumour volume data are shown. *P < 0.01, **P < 0.01, ***P < 0.001. **m**, Representative images of tumours after dissection and quantification of tumour weight of each treatment group. **n**, Representative H&E (scale bar indicates 50 µm, ×100), TFAM and MTCO2 immunofluorescence stainings (scale bar indicates 50 µm, ×400) of tumour tissues including quantification. NS, not significant. Source data

We observed that the half-life of TFAM protein was shorter in *VHL*^−/−^ or *EGLN3*^−/−^ cells compared to cells expressing *VHL* or *EGLN3*, demonstrating that pVHL and EGLN3 stabilize TFAM protein (Fig. 3h,i). Recent reports demonstrated that phosphorylation of TFAM by PKA promotes its degradation by the mitochondrial LONP1 protease^31,32^. Thus, we hypothesized that binding of pVHL to hydroxy-P53/P66-TFAM masks S55/S56 phosphorylation and LONP1 recognition site and thus prevents degradation by LONP1. First, we used the LONP1 protease inhibitor bortezomib to determine if low TFAM abundance in *EGLN3*^−/−^ primary MEFs and *VHL*-null 786-O cells was due to protease degradation. Bortezomib increased TFAM protein abundance in *EGLN3*^−/−^ MEFs to the level of *EGLN3*^+*/*+^ MEFs (Fig. 5a). Likewise, bortezomib treatment of *VHL*^−/−^ cells (786-O) restored TFAM levels (Fig. 5b), providing evidence that loss of either *EGLN3* or *VHL* accelerates TFAM degradation. In addition, we observed that exogenous expression of inducible TFAM-p.Pro53Arg/p.Pro66Arg mutant in HEK293 cells was robustly decreased compared to TFAM wild type (Fig. 5c). As shown earlier, TFAM-p.Pro53Arg/p.Pro66Arg mutant peptide failed to capture S35-VHL (Fig. 4g). We predict that TFAM-p.Pro53Arg/p.Pro66Arg protein can no longer bind pVHL, and therefore can be targeted by PKA phosphorylation and rapid LONP1 degradation. Thus, we investigated further if PKA phosphorylation of hydroxylated TFAM peptide is impaired by VHL binding. Preincubation with GST-VHL prevented the hydroxylated TFAM peptide from PKA binding and phosphorylation (Fig. 5d). Consistent with these results, we observed that PKA activation using forskolin in HA-VHL-WT-expressing cells (786-O) decreased TFAM protein to a similar level as observed in *VHL*-null cells (Fig. 5e). In a reciprocal experiment using the PKA inhibitor H89 in 786-O cells, we observed increased TFAM protein abundance in *VHL*-null cells to a similar level as observed in HA-VHL-WT-expressing cells (Fig. 5f).

Next, we tested the hypothesis that free TFAM (not bound to mitochondrial DNA) is resistant to LONP1 degradation when bound to VHL. Purified TFAM and LONP1 were incubated with ATP/Mg^2+^ causing TFAM to be rapidly degraded (Fig. 5g). In contrast, when purified TFAM was hydroxylated by EGLN3 and subsequently incubated with purified GST-VHL before LONP1 incubation, TFAM became resistant to LONP1 degradation (Fig. 5g). Hydroxylation assay with a catalytically dead p.His196Arg *EGLN3* mutant, however, impaired GST-VHL binding and TFAM was rapidly degraded by LONP1 despite incubation with GST-VHL.

Thus, we conclude that binding of pVHL to hydroxylated TFAM prevents TFAM from LONP1 recognition and degradation and thus allows free TFAM protein to stabilize in the absence of mitochondrial DNA binding.

### LONP1 inhibition sensitizes *VHL*-null clear cell renal cell carcinoma cells to sorafenib

The decrease of mitochondrial content observed in the *VHL* mutated PPGL and ccRCC cells (Fig. 1) suggests that mitochondrial content is a pathogenic target within the VHL cancer syndrome. Indeed, high expression of either catalytic subunit of PKA (PRKACA) or LONP1 that facilitates TFAM degradation was associated with shorter overall survival of individuals with ccRCC (Fig. 5h,i). Thus, we hypothesized that the decreased mitochondrial content in ccRCC might contribute to therapy resistance and contribute to lower overall survival in these individuals. *VHL*-null 786-O cells were resistant to apoptosis in contrast to *VHL* wild-type-expressing cells when treated with sorafenib (Fig. 5j,k). Sorafenib is a kinase inhibitor approved for the treatment of primary kidney cancer, advanced primary liver cancer, acute myeloid leukaemia and advanced thyroid carcinoma. Indeed, *VHL*-null cells pretreated with LONP1 inhibitor bortezomib were re-sensitized to sorafenib and underwent complete apoptosis similar to *VHL* wild-type-expressing cells (Fig. 5j,k). This suggests that the low level of mitochondrial content might contribute to therapy resistance.

To examine if *VHL*-null ccRCC can be re-sensitized to sorafenib treatment in vivo, we subcutaneously transplanted *VHL*-null 786-O cells into immunocompromised SCID mice. After approximately 4 weeks of tumour growth, mice were treated with DMSO (control), sorafenib (15 mg per kg body weight), bortezomib (1 mg per kg body weight), or with combination of both, sorafenib and bortezomib (Fig. 5l,m). Treatment with sorafenib or bortezomib alone did not significantly inhibit tumour growth compared with control treatment (Fig. 5l,m); however, combination treatment with sorafenib and bortezomib resulted in significant inhibition of tumour growth compared with single or control treatment (Fig. 5l,m), recapitulating our in vitro cell culture observation (Fig. 5j,k). Immunofluorescence stainings confirmed that combination treatment resulted in increased TFAM and mitochondrial protein MTCO2 (Fig. 5n). Collectively, these results indicate that *VHL*-null ccRCC cells are sensitized to sorafenib when combined with LONP1 inhibitor bortezomib, leading to a profound tumour growth defect in vivo that was associated with increased levels of mitochondrial content.

### VHL restores oxygen consumption independent of HIFα

**Fig. 6:**
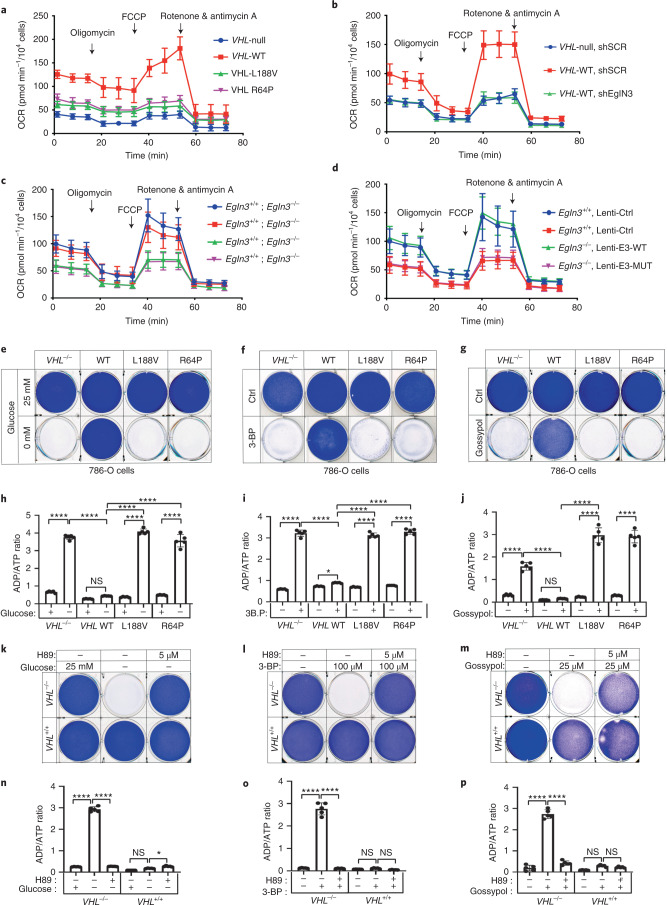
pVHL restores cellular oxygen consumption rate. **a**, Mitochondrial respiration reflected by OCR of 786-O cells with the indicated genotypes was monitored using the Seahorse XF-96 Extracellular Flux Analyzer with the sequential injection of oligomycin (1 μM), FCCP (1 μM) and rotenone/antimycin (0.5 μM). **b**–**d**, OCR measurement in 786-O cells with indicated VHL status transduced with lentiviral pL.KO shRNA targeting *EGLN3* or no targeting control (**b**), primary *EGLN3*^+*/*+^ and *EGLN3*^*−/−*^ MEFs (**c**) stably transduced with lentivirus encoding EglN3 WT, catalytic death mutant or empty control (**d**). In **a**–**d**, data are presented as mean values ± s.d. *n* = 3 biological independent experiments. **e**, Crystal violet staining of 786-O cells with the indicated VHL status treated with high glucose (25 mM) or no glucose (0 mM) for 36 h. Corresponding ADP/ATP ratio is shown in **h**. **f**, Crystal violet staining of 786-O cells with the indicated VHL status treated with 100 μM 3-BP for 4 h. Corresponding ADP/ATP ratio is shown in **i**. **g**, Crystal violet staining of 786-O cells with the indicated VHL status treated with 25 μM gossypol for 36 h. Corresponding ADP/ATP ratio is shown in **j**. In **h**–**j**, data are presented as mean values ± s.d. One-way ANOVA Tukey’s multiple-comparison test. *P < 0.05, ****P < 0.0001. *n* = 3 biological independent experiments. **k**, Crystal violet staining of 786-O cells with the indicated VHL status treated with 5 μM PKA inhibitor H89 for 24 h, before glucose deprivation for 36 h. Corresponding ADP/ATP ratio is shown in **n**. **l**, Crystal violet staining of 786-O cells treated with 5 μM PKA inhibitor H89 for 24 h, before 100 μM 3-BP treatment for 4 h. Corresponding ADP/ATP ratio is shown in **o**. **m**, Crystal violet staining of 786-O cells treated with 5 μM PKA inhibitor H89 for 24 h, before 25 μM gossypol for 36 h. Corresponding ADP/ATP ratio is shown in **p**. In **n**–**p**, data are presented as mean values ± s.d. One-way ANOVA Tukey’s multiple-comparison test. *P < 0.05, ****P < 0.0001. *n* = 3 biological independent experiments. Source data

**Extended Data Fig. 5:**
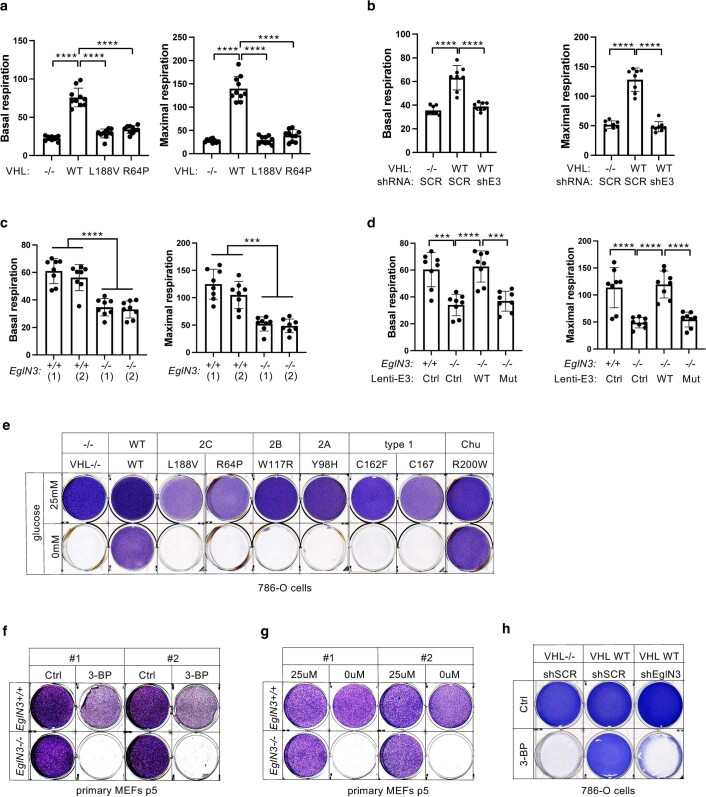
VHL restores cellular oxygen consumption rate. (a) Seahorse XF-96 analysis of oxygen consumption rate (OCR). Mitochondrial respiration reflected by OCR levels was detected in 786-O cells with indicated genotype. The rates of basal respiration and maximal respiratory capacity were respectively quantified by normalization of amount of cells. One way ANOVA Tukey’s Multiple Comparison Test. ****p < 0.0001. (b) Seahorse XF-96 analysis of oxygen consumption rate (OCR) of 786-O cells with indicated VHL status transduced with lentiviral pL.KO shRNA targeting EGLN3 or no targeting control. The rates of basal respiration and maximal respiratory capacity were respectively quantified as described above. One way ANOVA Tukey’s Multiple Comparison Test. ****p < 0.0001. (c) Seahorse XF-96 analysis of oxygen consumption rate (OCR) of primary EGLN3 + / + and EGLN3-/- MEFs. The rates of basal respiration and maximal respiratory capacity were respectively quantified by normalization of amount of cells. One way ANOVA Tukey’s Multiple Comparison Test. ***p < 0.001, ****p < 0.0001. (d) Seahorse XF-96 analysis of oxygen consumption rate (OCR) of primary EGLN3-MEFs of indicated genotype stably transduced with lentivirus encoding EGLN3 WT, catalytic death mutant or empty control. The rates of basal respiration and maximal respiratory capacity were respectively quantified as described above. ***p < 0.001, ****p < 0.0001. **a-d**, data are presented as mean values ± SD. n = 3 biological independent experiments. (e) Crystal violet staining of 786-O cells with indicated VHL status treated with high glucose (25 mM) or no glucose respectively for 36 hours. (f) Crystal violet staining of primary EGLN3 + / + and EGLN3-/- MEFs treated with 100 μM 3-bromopyruvic acid (3-BP) for 4 hours. (g) Crystal violet staining of primary EGLN3 + / + and EGLN3-/- MEFs treated with high glucose (25 μM) or no glucose (0 μM) respectively for 48 hours. (h) Crystal violet staining of 786-O cells with indicated VHL status transduced with lentiviral pL.KO shRNA targeting EGLN3 or no targeting control, treated with 100 μM 3-bromopyruvic acid (3-BP) for 4 hours. Source data

**Extended Data Fig. 6:**
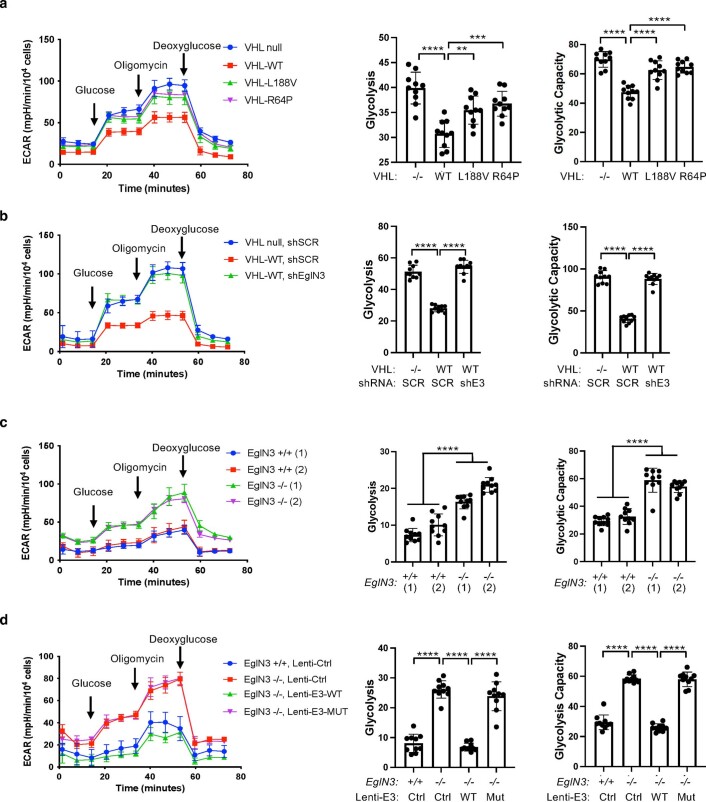
VHL decreases glycolysis. (a) Extracellular acidification rate (ECAR) of 786-O cells with indicated genotype was monitored using the Seahorse XF-96 Extracellular Flux Analyzer with the sequential injection of glucose (10 mM), oligomycin (1 μM) and 2-deoxy-glucose (2-DG) (50 μM). The rates of glycolysis and glycolysis capacity were respectively quantified by normalization of amount of cells. One way ANOVA Tukey’s Multiple Comparison Test. **p = 0.003, ***p = 0.0002, ****p < 0.0001. (b) Extracellular acidification rate (ECAR) of 786-O cells with indicated VHL status transduced with lentiviral pL.KO shRNA targeting EGLN3 or no targeting control was measured as described above. The rates of glycolysis and glycolysis capacity were respectively quantified by normalization of amount of cells. One way ANOVA Tukey’s Multiple Comparison Test. ****p < 0.0001. (c) Extracellular acidification rate (ECAR) of primary EGLN3 + / + and EGLN3-/- MEFs. The rates of glycolysis and glycolysis capacity were respectively quantified by normalization of amount of cells. One way ANOVA Tukey’s Multiple Comparison Test. ****p < 0.0001. (d) Extracellular acidification rate (ECAR) of primary EGLN3-MEFs of indicated genotype stably transduced with lentivirus encoding EGLN3 WT, catalytic death mutant or empty control was monitored as described above. The rates of glycolysis and glycolysis capacity were respectively quantified by normalization of amount of cells. One way ANOVA Tukey’s Multiple Comparison Test. ****p < 0.0001. **a-d**, data are presented as mean values ± SD. n = 3 biological independent experiments. Source data

We observed that mitochondrial mass can be restored by wild-type *VHL*, but not type 2C *VHL* mutants that are normal regarding HIFα regulation (Fig. 1f). Thus, we tested if the restored mitochondrial content resulted in functional mitochondria and increased cellular oxygen consumption rate (OCR). Compared to 786-O *VHL*-null cells, overall respiration was significantly increased in 786-O cells stably expressing wild-type *VHL*, but not in cells expressing type 2C *VHL* mutants (Fig. 6a and Extended Data Fig. 5a), indicating that *VHL* can restore mitochondrial function independent of HIFα regulation. Glycolysis and glycolytic capacity measured by extracellular acidification rate (ECAR) in VHL type 2C cells was, however, in between *VHL*-null and *VHL* wild-type cells, suggesting that activation of HIFα in *VHL*-null cells additionally contributes to increased glycolysis (Extended Data Fig. 6a).

Consistent with our findings that pVHL-mediated regulation of mitochondrial mass is EGLN3 dependent (Fig. 2b–d), cellular respiration was impaired in *VHL*-expressing 786-O cells after inactivation of *EGLN3* by an effective shRNA (Fig. 6b and Extended Data Fig. 5b).

Consistent with these observations, glycolysis was increased after inactivation of *EGLN3* by shRNA (Extended Data Fig. 6b). Furthermore, OCR was impaired (Fig. 6c and Extended Data Fig. 5c) and glycolysis was increased (Extended Data Fig. 6c) in primary *EGLN3*^−/−^ MEFs (passage 5) compared to control *EGLN3*^+*/*+^ MEFs. This was dependent on EGLN3 enzymatic activity, as respiration was restored in *EGLN3*^−/−^ MEFs that were transduced with wild-type *EGLN3* (lenti-EGLN3-WT), but not when transduced with catalytically inactive mutant (lenti-EGLN3-H196A; Fig. 6d and Extended Data Figs. 5d and 6d).

To test if the low mitochondrial content in *VHL*-null cells will result in a glycolytic dependency to maintain energy homeostasis, we performed glucose deprivation or glycolysis inhibition and measured the ADP:ATP ratio, a central parameter of cellular energy metabolism (Fig. 6e–p). The 786-O cells expressing *VHL* wild type were resistant to glucose deprivation-induced cell death, compared to *VHL*-null cells or cells expressing *VHL* type 2C, 2B, 2A or type 1 mutants (Fig. 6e and Extended Data Fig. 5e). Similar results were observed inhibiting glycolysis using hexokinase inhibitor (3-bromopyruvatic acid (3-BP)) or lactate dehydrogenase inhibitor gossypol (Fig. 6f,g). Glycolytic dependency was similarly observed in *EGLN3*-null cells (Extended Data Fig. 5f–h). Furthermore, an unbalanced ADP/ATP ratio was significantly induced after glucose deprivation or glycolysis inhibition in *VHL*-null or type 2C mutant cells but not in *VHL* wild-type-expressing cells, indicating a cellular response to energy crisis (Fig. 6h–j). Since PKA inhibitor (H89) restored TFAM expression in *VHL*-null cells, we explored if PKA inhibition can restore resistance to glycolysis inhibition in *VHL*-null cells. Similarly to cells expressing wild-type *VHL*, *VHL*-null cells showed resistance to glucose deprivation and glycolysis inhibition when pretreated with PKA inhibitor, and the ADP/ATP ratio was restored (Fig. 6k–p).

In summary, 786-O cells lacking *VHL* or expressing type 2C *VHL* mutants depend on glycolysis to obtain energy. Restoring wild-type *VHL* or pretreating cells with PKA inhibitor restores mitochondrial content and function and promotes metabolic reprogramming to oxidative phosphorylation and thus reverses vulnerability to glycolysis inhibition-induced cell death.

### Low mitochondrial content causes impaired differentiation

**Fig. 7:**
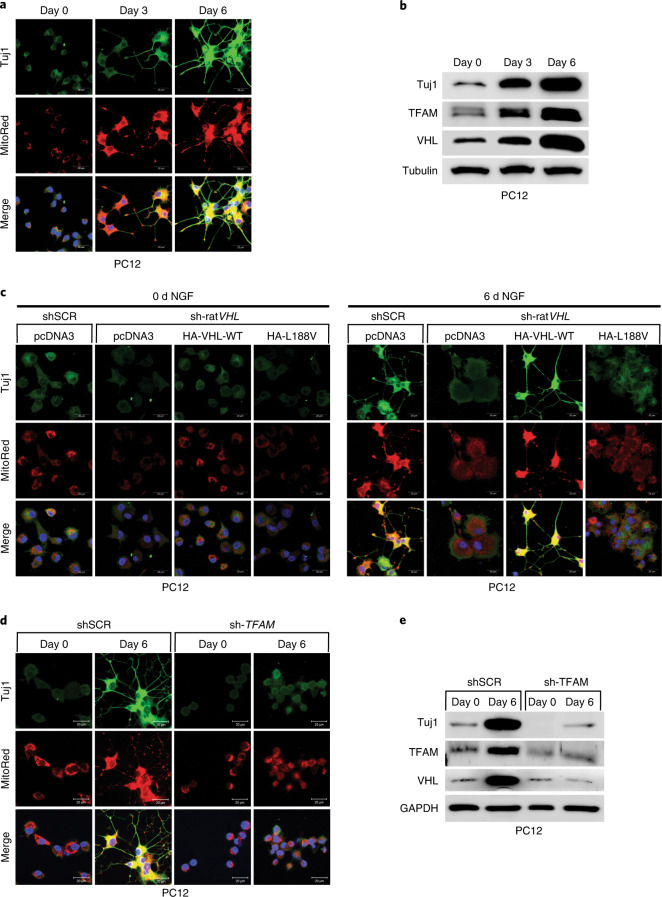
Low mitochondrial content in pheochromocytoma cells causes impaired differentiation. **a**, Fluorescence images of PC12 cells treated with 50 ng ml^−1^ NGF at the indicated time points. Cells were stained by MitoTracker Red to visualize mitochondria and endogenous Tuj1 (neuron-specific class III beta-tubulin) was stained in green. **b**, Corresponding immunoblot analysis. *n* = 3 biological independent experiments. **c**, Fluorescence images of stable polyclonal PC12 cells expressing the indicated human *VHL* (hu*VHL*) species selected with G418 (0.5 mg ml^−1^) for 2 weeks. PC12 clones were transduced for 48 h with lentivirus encoding shRNA targeting endogenous rat *VHL* (sh-rat*VHL*) or scramble control (shSCR) and subsequently treated with NGF for 6 d. Cells were stained by MitoTracker Red to visualize mitochondria and endogenous Tuj1 was stained in green. **d**, Fluorescence images of polyclonal PC12 cells transduced for 48 h with lentivirus encoding shRNA targeting endogenous rat *TFAM* (sh-*TFAM*) or scramble control (shSCR) and subsequently treated with NGF for 6 d. Cells were stained by MitoTracker Red to visualize mitochondria and endogenous Tuj1 in green. **e**, Corresponding immunoblot analysis. *n* = 3 biological independent experiments. In **a**, **c** and **d**, similar results were observed more than three times. Source data

**Extended Data Fig. 7:**
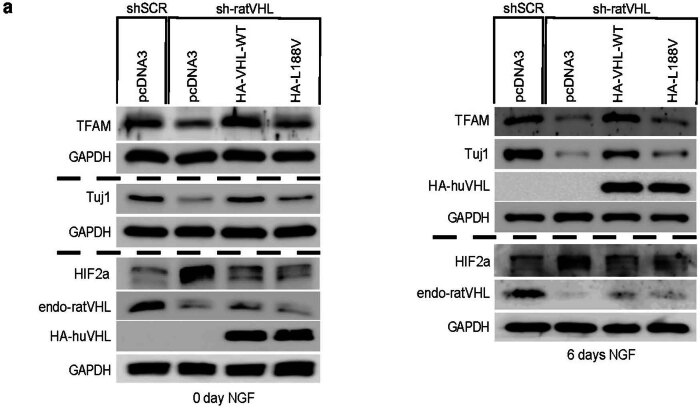
Low mitochondrial content in pheochromocytoma cells causes impaired differentiation. (a) Immunoblot analysis of stable polyclonal PC12 cells expressing the indicated human VHL (huVHL) species. Stable polyclonal PC12 cells were transduced for 48 h with lentivirus encoding shRNA targeting endogenous rat VHL (endg. sh-ratVHL) or scramble control (shSCR) and subsequently treated with NGF for 6 days. n = 3 biological independent experiments. Source data

It has been recently demonstrated that metabolic reprogramming from aerobic glycolysis to oxidative phosphorylation is tightly coupled to differentiation, although the exact molecular basis underlying the transition is unknown^34,35^. Thus, we asked if type 2C *VHL* cancer mutations contributing to low mitochondrial content can impair differentiation in pheochromocytoma PC12 cells. PC12 cells have been used as a model to study differentiation by nerve growth factor (NGF)^36^. PC12 cells resemble differentiated sympathetic neurons when grown under low-serum conditions in the presence of NGF^37^ (Fig. 7a,b). Neurite outgrowth and the induction of neuron-specific class III beta-tubulin (Tuj1) was evident within 6 d of NGF culture condition. Differentiation was accompanied by induction of mitochondrial mass measured by MitoTracker staining and induction of TFAM protein (Fig. 7a,b). Next, we generated stable PC12 cells expressing either human HA-*VHL* wild-type or type 2C *VHL* mutants and subsequently transduced cells with an effective shRNA silencing endogenous rat *VHL* (Extended Data Fig. 7a). Control cells (shSCR) grown in the presence of NGF differentiated as evidenced by neurite outgrowth and Tuj1 induction. However, cells transduced with an effective shRNA inactivating endogenous rat *VHL* (sh-rat*VHL*) failed to respond to NGF-mediated differentiation (Fig. 7c). In contrast, cells with restored expression of human HA-*VHL* fully rescued the differentiation induced by NGF as evident by neurite outgrowth, Tuj1 induction and induction of mitochondrial content. In contrast, cells restored with HA-*VHL*-p.Leu188Val type 2C mutant showed no differentiation-associated phenotypic changes or induction of Tuj1 or mitochondrial content (Fig. 7c and Extended Data Fig. 7a). Similarly to inactivation of *VHL*, inactivation of *TFAM* following transduction with an effective shRNA, PC12 cells showed no characteristics of phenotypical changes associated with NGF-induced differentiation (Fig. 7d,e), indicating that low mitochondrial content prevents neural differentiation induced by NGF.

## Discussion

**Fig. 8:**
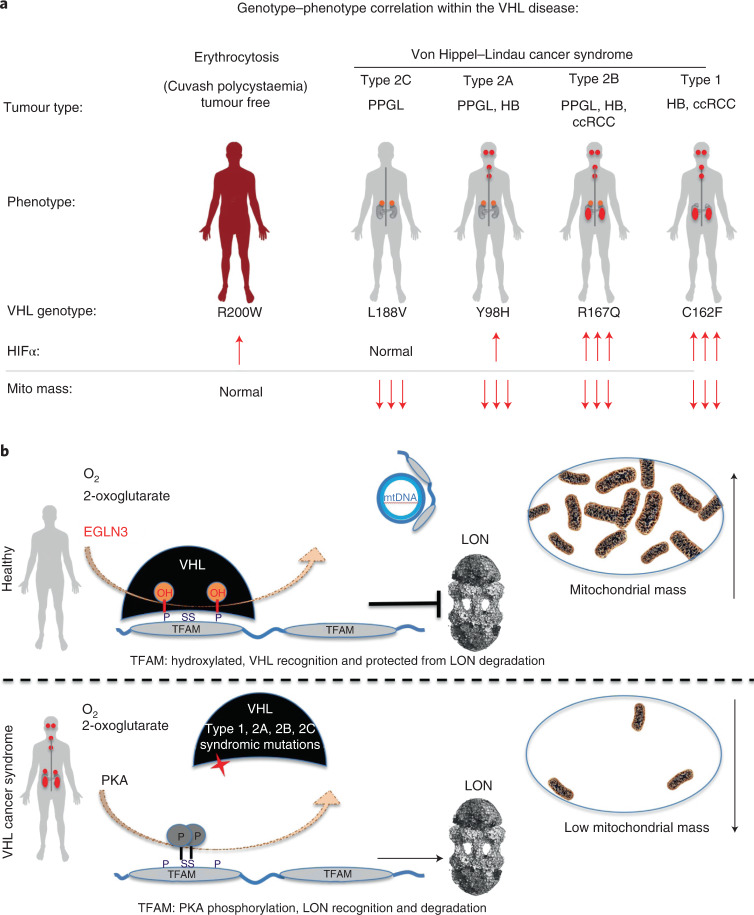
Schematic of oxygen-dependent regulation of mitochondrial content within the von Hippel–Lindau syndrome. **a**, Genotype–phenotype correlation in cancers arising in the VHL syndrome and its association with regulation of HIFα and mitochondrial content. Note that the Cuvash polycythaemia mutation *VHL*^*R200W*^ shows total absence of tumour development despite increased HIFα signalling and appears normal with regard to regulating mitochondrial content. **b**, Schematic of oxygen-dependent regulation of mitochondrial transcription factor TFAM by pVHL, independent of the canonical substrate HIFα. mtDNA, mitochondrial DNA.

Functional mitochondria are essential for the cell energy metabolism of most tumour types. At the same time, mutations in genes impairing oxidative phosphorylation causing defects in mitochondrial energy metabolism have been reported for a restricted subset of tumours such as succinate dehydrogenase in hereditary PPGL, RCC and gastrointestinal stromal tumours^38^, fumarate hydratase in hereditary leiomyomatosis and RCC^39^ and isocitrate dehydrogenase 1 (*IDH1*) and *IDH2* in secondary glioblastomas and acute myeloid leukaemia^40,41^. Here, we observed that all tested *VHL* cancer syndrome mutations (type 1 and type 2A, 2B and 2C), but not the *VHL*^R200W^ Chuvash polycythaemia mutation, are impaired in regulating TFAM abundance and contribute to decreased mitochondrial mass (Fig. 8). Patients with *VHL*^R200W^ mutation causing Chuvash polycythaemia are reported to show total absence of tumour development despite increased HIFα signalling^22–24^. Thus, alterations in mitochondrial biogenesis might play a role in initiating and/or sustaining the transformed state, independent of HIFα oncogenic functions. In this regard, we found that low mitochondrial content in pheochromocytoma cells (PC12) expressing type 2C *VHL* mutants prevented NGF-induced differentiation. Type 2C *VHL* mutations were clearly defective in binding hydroxylated TFAM and failed to restore mitochondrial content, despite their ability to suppress HIFα. Impaired mitochondrial biogenesis caused by germline VHL syndrome mutations might impair differentiation of a progenitor cell during embryonic development, independent of HIFα. In this regard, in most affected *VHL* carriers, the disease displays an autosomal dominant pattern of inheritance^42,43^. This is in contrast with gain-of-function HIF2α mutations that are observed in only few sporadic cases of PPGL and have not been detected to date in ccRCC^6–8^. Although HIF2α is considered to be an oncogenic driver in *VHL*-related ccRCC, familial gain-of-function HIF2a mutations have only been reported to be associated with familial erythrocytosis^44^. Similarly to *VHL*-Chuvash polycythaemia, patients with familial gain-of-function HIF2α mutation had no history of RCC, PPGL or central nervous system HB, the hallmarks of the VHL syndrome. This suggests additional pVHL tumour-suppressor functions outside HIFα regulation and implies that activation of HIFα might be necessary, but not sufficient for driving tumorigenesis in the VHL cancer syndrome (Fig. 8a). Furthermore, recent data show that *VHL*-related ccRCC can be classified into HIF2α-dependent and HIF2α-independent tumours and that these tumours differ in HIF2α levels and in their gene expression^45^. These observations also point to HIF-independent mechanisms of tumorigenesis downstream of pVHL and thus may underlie differences in responsiveness to HIF2a inhibitor^45^.

Non-cancerous *VHL*^R200W^ Chuvash polycythaemia mutation was normal with regard to TFAM regulation in contrast to all other *VHL* syndromic cancer mutations, which suggests that impaired mitochondrial biogenesis is an important feature of the VHL syndrome (Fig. 8a). Low mitochondrial content could provide an energetic vulnerability for all tumour types arising in the VHL syndrome, including type 2C PPGL. In this regard, we observed that *VHL*-null ccRCC cells or cells expressing type 2C *VHL* mutations are highly dependent on glycolysis to maintain energy homeostasis and undergo rapid cell death when treated with glycolysis inhibitors. Efforts to target glucose uptake or lactate production, however, have been limited due to toxicity associated with hypoglycaemia symptoms^46^.

Advanced ccRCC is a lethal disease with a 5-year survival of only 11.7%^47^, and traditional chemotherapy and radiation therapy are largely ineffective. We hypothesized that the low mitochondrial content in ccRCC might contribute to the known therapy resistance in ccRCC. *VHL*-null 786-O cells were resistant to apoptosis in contrast to *VHL* wild-type-expressing cells when treated with sorafenib, a multi-kinase inhibitor approved for the treatment of primary kidney cancer. By understanding the precise molecular mechanism by which pVHL regulates mitochondrial mass, we performed pharmacological studies to increase mitochondrial content in VHL-deficient ccRCC cells. pVHL binding to hydroxylated TFAM, a key activator of mitochondrial transcription and replication, stabilizes TFAM by preventing LONP1 recognition and subsequent mitochondrial proteolysis (Fig. 8b). *VHL*-null ccRCC cells responded to LONP1 inhibitor bortezomib, causing an increase of mitochondrial content and re-sensitized cells to sorafenib. Combined treatment of both sorafenib and bortezomib provided a profound tumour growth defect in vivo. Thus, LONP1 inhibition provides a pharmacological tool to increase mitochondrial content in VHL-deficient ccRCC and can sensitize therapy-resistant ccRCC cells to sorafenib.

## Methods

### Ethical considerations

Collection and analyses of human samples (normal adrenal tissues, PPGL) are covered by the ethical approval numbers 01-136, KI forskningsetikkommitté Nord and 2020-04226. All samples were obtained following an informed patient consent. Ethical permits for animal studies were approved by the appropriate local and national authorities (Jordbruksverket, Sweden).

### Cell culture

Human renal carcinoma cell lines 786-O (American Type Culture Collection (ATCC), CRL-1932) and A498 (ATCC, HTB-44), HeLa cells (ATCC, CCL-2), 293FT cells (Thermo Fisher, R70007) and MEFs were cultured in DMEM (glucose 4.5 g l^−1^) containing 10% FBS in 5% CO_2_ at 37 °C. *VHL*^fl/fl^ and *EGLN3*^−/−^ primary MEFs^48^ and isolation of primary MEFs^49^ have been described previously. 786-O cells were purchased from the ATCC. Rat PC12 cells (ATCC, CRL-1721) were differentiated with 50 ng ml^−1^ NGF in DMEM medium (glucose 1 g l^−1^) supplemented with 1% horse serum. Before differentiation, stable polyclonal PC12 cells expressing the indicated human *VHL* (hu*VHL*) species were selected with G418 (0.5 mg ml^−1^) for 2 weeks. PC12 clones were transduced for 48 h with lentivirus encoding shRNA targeting endogenous rat *VHL* (sh-rat*VHL*) or scramble control (shSCR) and subsequently treated with NGF for 6 d.

### *EGLN3*-knockout mice

Generation of the *EGLN3* mouse strain (C57BL/6) has been previously described^48^. Animal experiments were performed in accordance with Swedish animal welfare laws authorized by the Stockholm Animal Ethics Committee (7694/17). P1 SCG dissections of *EGLN3* pups are described in ref. ^50^. Mice were housed in individually ventilated cages with free access to food and water in constant temperature (20 ± 3 °C) and humidity (50% ± 10%). Light/dark cycle was 12 h:12 h from 06.00 to 18.00 and 12 h of darkness with dusk and dawn periods in between. The mice received standard diet from Special Diets Services CRM (P; 9.5 mm pelleted; 801722).

### Human tissue specimens

Tumour tissue samples (PCC *n* = 9 and PGL *n* = 1) were collected from individuals at the Karolinska University Hospital, Stockholm, Sweden, and previously characterized for mutations in PPGL susceptibility genes^51^ (Extended Data Fig. 2). All samples were obtained with informed patient consent and with approval from the local ethical committees. *VHL* mutations in cases 21, 25, 96 and 108 as well as WT-*VHL* status for the other six cases have been previously described^51^.

### Confocal microscopy

MEFs and 786-O cells were cultured on glass coverslips and stained with MitoTracker Red CMXRos (100 nM) at 37 °C for 30 min, washed twice with pre-warmed PBS, and fixed for 15 min in pre-warmed 4% paraformaldehyde. Coverslips were immersed into PBS overnight, and mounted using ProLong Diamond Antifade Mountant with DAPI (Thermo Fisher, P36962). Fluorescence images were acquired using a Zeiss LSM 700 laser scanning confocal microscope equipped with a ×63 Plan-Apochromat/1.4-NA Oil with DIC capability objective. The excitation wavelengths for MitoTracker Red CMXRos and DAPI were 579 nm and 405 nm, respectively. Images were acquired using the settings: frame size of 1,024, scan speed of 6 and 12-bit acquisition and line averaging mode of 8. Pinholes were adjusted so that each channel had the same optical slice of 1–1.2 μm.

### Flow cytometry analysis

786-O or MEFs cells were stained with MitoTracker Green FM (100 nM) at 37 °C for 30 min for labelling mitochondria. Mean fluorescence intensity analysis of labelled mitochondria was performed by gating on single cells (see FACS gating strategies in Supplementary Fig. 1). Samples were analysed on an LSRFortessa flow cytometer (BD Biosciences) and analysed using FlowJo software (Tree Star). Cell apoptosis rate was detected by annexin V-FITC/PI staining. After 48 h of treatment, the 786-O cells were rinsed with PBS and collected for annexin V-FITC/PI staining. Each cell pellet was resuspended in 500 μl of binding buffer supplemented with 5 μl of FITC and 5 μl of PI, and the cells were incubated for 15 min. The apoptotic ratios were determined by flow cytometry.

### Graded treadmill running test

All treadmill running experiments were approved by the regional animal ethics committee of Northern Stockholm (4039-2018 and 4359-2020) and mice were housed as described above. Male mice aged 58–60 weeks old (wild type *n* = 15, KO *n* = 16) and young males 18–19 weeks old (wild type *n* = 16 and KO *n* = 16) were used. Acclimation to treadmill was performed with male wild-type and *EGLN3*^−/−^ mice of the indicated ages for 3 d before the experiment by running 10 min per day. Each day, mice started with 5 min at a speed of 6 m min^−1^. On day 1, this was followed by 5 min at 9 m min^−1^. On day 2, this was followed by 2 min at 9 m min^−1^, 2 min at 12 m min^−1^ and 1 min at 6 m min^−1^. On day 3, this was followed by 2 min at 9 m min^−1^, 2 min at 15 m min^−1^ and 1 min at 6 m min^−1^. The graded treadmill running test was performed on a 10° slope. During the test, the speed was increased every 3 min up to a maximum of 35 m min^−1^. Exhaustion time was determined when the animal could no longer continue running despite gentle prodding. Body weight was recorded after exhaustion to calculate work and power.

### Mouse tumour models and treatment

Mouse tumour xenograft models were approved by the Swedish Board of Agriculture (ethical no. 6197-2019). Male CB17/Icr-*Prkdc*^scid^/Rj mice aged 6–8 weeks old were purchased from Janvier Labs (France). Mice were housed in individually ventilated cages with free access to food and water in constant temperature (20 ± 3 °C) and humidity (50% ± 10%). Light/dark cycle was 12 h:12 h from 06.00 to 18.00 and 12 h of darkness with dusk and dawn periods in between. The mice received standard diet from Special Diets Services CRM (P; 9.5 mm pelleted; 801722). They were randomly divided into each group. Approximately 5 × 10^6^ 786-O tumour cells were subcutaneously injected into the back along the middorsal line of each mouse. Tumour volume was measured every 3 d and calculated according to the standard formula: length × width^2^ × 0.52. Drug treatment was initiated when tumour volume reached 5 mm^3^. DMSO (1%; STBJ9836, SIGMA) and sorafenib (15 mg per kg body weight, SML2653, Sigma-Aldrich) were orally delivered to mice every day. Bortezomib (1 mg per kg body weight; 3514175, Merck) was intraperitoneally injected twice per week in either monotherapy or combination therapy. The experiment terminated when the tumour volume of the 1% DMSO group reached 1.2–1.3 cm^3^. The maximal tumour size permitted by the ethics committee was 2.5 cm^3^ and the tumour size did not exceed the permitted tumour size.

### Histology and immunofluorescence

Paraffin-embedded tissues were cut into 5-μm slides. Slides were baked for 1 h at 60 °C and deparaffinized in Tissue-Clear (1466, Sakura), and sequentially rehydrated in 99%, 95% and 70% ethanol and counterstained with H&E. Mounting was performed with Pertex (0081, HistoLab). Deparaffinized slides were boiled for 20 min in an unmasking solution (H3300, Vector Labs) then subsequently blocked with 4% serum. Tissues were incubated with a mouse anti-human mtTFA antibody (1:200 dilution; 119684, Abcam) and a mouse anti-human MTCO2 antibody (1:200 dilution; 110258, Abcam) at 4 °C overnight, followed by staining with a species-matched secondary Alexa Fluor 555-labelled donkey anti-mouse (1:400 dilution; A-31570, Thermo Fisher Scientific) and a DAPI (10236276001, Roche). Slides were mounted with Vectashield (H-1000, Vector Labs). Signals were detected by fluorescence microscope equipped with a camera (Nikon, DS-QilMC). Images were analysed using an Adobe Photoshop software (CC 2019, Adobe) programme.

### Viruses

Lentiviruses encoding wild-type Flag*-EGLN3* and catalytically dead Flag-*EGLN3* with the p.His196Arg mutation (Flag-EGLN3-H196A) were generated via TOPO cloning using pLenti6.3 backbone from Invitrogen (Life Technologies).

### Immunoblot analysis

The lysis of cell lines, mouse and human tissue was performed in EBC buffer (50 mM Tris at pH 8.0, 120 mM NaCl, 0.5% NP-40) containing phosphatase inhibitors (04906837001, Sigma) and protease inhibitors (11697498001, Roche Life Science). Proteins were quantified by Bradford assay, and samples containing equal protein amounts were immunoblotted using previously described methodology^52^. Quantification of western blots was performed by ImageJ (Supplementary Fig. 2)

Antibodies used were: rabbit monoclonal anti-TFAM (1:1,000 dilution; Cell Signaling Technology, 8076), rabbit polyclonal anti-PKA C-α (1:1,000 dilution; Cell Signaling Technology, 4782), rabbit monoclonal anti-PHD-2/Egln1 (1:500 dilution; Cell Signaling Technology, 4835), rabbit monoclonal anti-HIF2α (1:1,000 dilution; Cell Signaling Technology, 7096), rabbit polyclonal anti-TOM20 (1:2,000 dilution; Cell Signaling Technology, 13929), rabbit polyclonal anti-TFAM (1:1,000 dilution; Abcam, ab131607), mouse monoclonal anti-GAPDH (1:2,000 dilution; Abcam, ab8245), mouse monoclonal anti-OXPHOS (1:1,000 dilution; Abcam, ab110413), mouse monoclonal anti-MT-CO1 (1:1,000 dilution; Abcam, ab14705), rabbit polyclonal anti-MT-CO2 (1:1,000 dilution; Abcam, ab91317), rabbit polyclonal anti-MT-ND1 (1:1,000 dilution; Abcam, ab181848), rabbit polyclonal anti-MT-ATP6 (1:1,000 dilution; Abcam, ab192423), rabbit polyclonal anti-MT-CYB (1:1,000 dilution; Abcam, ab81215), rabbit polyclonal anti-LONP1 (1:1,000 dilution; Abcam, ab103809), rabbit polyclonal anti-tyrosine hydroxylase (1:1,000 dilution; Abcam, ab112), rabbit polyclonal anti-hydroxyproline (1:1,000 dilution; Abcam, ab37067, GR3215743-1 and GR3179915-1), rabbit monoclonal anti-cyclin D1 (1:1,000 dilution; Abcam, ab134175), rabbit polyclonal anti-HIF1α (1:500 dilution; Novus Biologicals, NB100-479), rabbit polyclonal anti-HIF2α (1:500 dilution; Novus Biologicals, NB100-122), mouse monoclonal anti-α-tubulin (1:2,000 dilution; Sigma-Aldrich, T5168), mouse monoclonal anti-HA (1:1,000 dilution; Sigma-Aldrich, H9658), rabbit polyclonal anti-flag (1:1,000 dilution; Sigma-Aldrich, F7425), mouse monoclonal anti-PGC1α (1:1,000 dilution; Millipore, ST1202), mouse monoclonal anti-VHL (1:500 dilution; BD Biosciences, 556347), mouse monoclonal anti-VHL (1:1,000 dilution; BD Biosciences, 564183), rabbit polyclonal anti-EGLN2 (1:500 dilution; Affinity Biosciences, DF7918), mouse monoclonal anti-TUJ1 (1:2,000 dilution, Covance, MMS-435P), mouse monoclonal anti-c-Myc (1:1,000 dilution; Thermo Fisher Scientific, 13-2500) and mouse monoclonal anti-p-Ser (16B4; 1:500 dilution; Santa Cruz Biotechnology, sc-81514).

### Proteomics analyses by nanoLC–MS/MS

NanoLC–MS/MS including database search for protein identification and quantification were performed at the Proteomics Biomedicum core facility, Karolinska Institutet, Stockholm. For protein extraction, human tissues were homogenized and lysed in EBC buffer (50 mM Tris, pH 8; 0.5% NP-40 and 120 mM NaCl) and proteins in supernatant were precipitated with chilled acetone at −20 °C overnight. Proteins (50 µg) were solubilized in 1 M urea (Sigma-Aldrich), 50 mM ammonium bicarbonate in 10% acetonitrile (AcN) and reduced with dithiothreitol to a final concentration of 5 mM by incubation for 1 h at 25 °C and alkylated with iodoacetamide to a final concentration of 15 mM via incubation for 1 h at 25 °C in the dark. The excess iodoacetamide was quenched by adding an 10 mM dithiothreitol.

Digestion was performed with 1.5 µg trypsin (final enzyme-to-protein ratio of 1:30) at 37 °C overnight followed by additional proteolysis with 1 µg lys-C at 37 °C for 6 h. After acidification with formic acid (5% final concentration), the tryptic peptides were cleaned with C18 HyperSep Filter Plate (bed volume of 40 µl; Thermo Scientific) and dried in a speedvac (miVac, Thermo Scientific).

Tandem Mass Tag (TMT)-10plex reagents (Thermo Scientific) in 100-µg aliquots were dissolved in 30 µl dry AcN, scrambled and mixed with 25 µg digested samples dissolved in 70 µl of 50 mM TEAB (resulting in a 30% AcN final concentration), followed by incubation at 22 °C for 2 h at 450 rpm. The reaction was then quenched with 11 µl of 5% hydroxylamine at 22 °C for 15 min at 450 r.p.m. The labelled samples were pooled and dried in a speedvac (miVac, Thermo Scientific).

The TMT-labelled tryptic peptides were dissolved in 2% AcN/0.1% formic acid at 1 µg µl^−1^ and 2-µl samples were injected in an UltiMate 3000 nano-flow LC system online coupled to an Orbitrap Fusion mass spectrometer (Thermo Scientific). Peptides were separated by chromatography using a 50-cm-long C18 EASY-Spray column (Thermo Scientific), and 4–26% AcN for 120 min, 26–95% AcN for 5 min and 95% AcN for 8 min at a flow rate of 300 nl min^−1^. The mass spectrum ranged from a *m/z* of 375 to 1,600, acquired with a resolution of 120,000 (*m/z* of 200), followed by data-dependent HCD fragmentations of precursor ions with a charge state from 2+ to 7+, using 45 s of dynamic exclusion. The tandem mass scans were acquired with a resolution of 50,000, targeting 5 × 10^4 ^ions, setting isolation width to a *m/z* of 1.4 and normalized collision energy to 35%.

### Protein identification and quantification

Protein identification and quantification were performed with Proteome Discoverer v2.3 with human Swiss-Prot protein databases (21,008 entries) using the Mascot 2.5.1 search engine (Matrix Science). Parameters used were up to two missed cleavage sites for trypsin, precursor mass tolerance of 10 ppm and 0.02 Da for the HCD fragment ions. Quantifications used both unique and razor peptides.

### Pathway analysis

According to the fold change of the protein abundance in human VHL-mutant PPGL compared to *VHL* wild-type PPGL, the top 50 significantly upregulated and downregulated proteins (*P* value < 0.05, two-tailed unpaired *t*-test) were selected for protein network analysis. STRING v10.5 was used to map the top 50 upregulated and downregulated regulated proteins in human PCC/PGL tumours onto protein–protein interaction networks (https://string-db.org/) with a medium confidence threshold (0.4). To identify enriched GO terms and KEGG (Kyoto Encyclopedia of Genes and Genomes) pathways, in-built gene-set enrichment analysis with the whole genome background was used.

GO term enrichment in cellular components of significantly downregulated proteins (*P* value < 0.0001, two-tailed unpaired *t*-test) in *VHL*-null and *VHL*^*L188V*^ 786-O cells was performed using DAVID and plotted using REVIGO. GO term enrichment was performed using DAVID with the full human proteome supplied by DAVID used as the background list, and plotted to reduce redundancy using ReviGo. The size of the bubbles is indicative of the number of proteins annotated with that GO term; bubbles were colour coded according to significance.

### In vitro hydroxylation of full-length TFAM and ^35^S-VHL capture

In vitro hydroxylation and S^35^ capture have been recently described^20^. In short, Myc-TFAM, ^35^S-HA-VHL, Flag-*EGLN3* WT and Flag-*EGLN3* catalytically dead mutant were synthesized by IVT reactions using TnT T7 Quick Master Mix and used as substrates. IVT was added to 300 μl hydroxylation reaction buffer and 100 μM iron chloride, 2 mM ascorbate and 5 mM 2-oxoglutarate. Next, 15 μl IVT EGLN3 was added to the hydroxylation reaction for 2 h at room temperature. Then, 500 μl EBC lysis buffer was used to stop the hydroxylation reaction and 15 μl IVT-synthesized ^35^S-HA-VHL was added subsequently and incubated for 2 h. Myc-TFAM was immunoprecipitated with anti-c-Myc antibody overnight at 4 °C with rotation and captured with 70 μl (50% slurry) protein G beads. The beads pellet was washed five times with immunoprecipitation buffer (0.5% NP-40, 150 mM NaCl, 10 mM Tris-HCl). Immunoprecipitated protein complexes were eluted with Laemmli buffer, boiled and centrifuged. Supernatant was analysed by immunoblot or ^35^S autoradiography (Fig. 4a).

### Peptide synthesis

The following biotinylated peptides were synthesized by peptides&elephants:

Naïve TFAM: -SPFSFVYLPRWFSSVLASCPKKPVSSYLRFSKEQLPIFKA

TFAM-p.Pro50Arg mutant: -SPFSFVYLPRWFSSVLASCAKKPVSSYLRFSKEQLPIFKA

TFAM-p.Pro53Arg mutant: -SPFSFVYLPRWFSSVLASCPKKAVSSYLRFSKEQLPIFKA

TFAM-p.Pro66Arg mutant: -SPFSFVYLPRWFSSVLASCPKKPVSSYLRFSKEQLAIFKA

TFAM-p.Pro53/66Arg double mutant:

-SPFSFVYLPRWFSSVLASCPKKAVSSYLRFSKEQLAIFKA

Hydroxy-TFAM-P-OH-53/66:

-SPFSFVYLPRWFSSVLASCPKKP(OH)VSSYLRFSKEQLP(OH)IFKA

Naïve HIF1α: DLDLEMLAPYIPMDDDFQLR

Hydroxy-HIF1α-P-OH 564: DLDLEMLAP(OH)YIPMDDDFQLR

Naïve HIF2α: FNELDLETLAPYIPMDGEDFQLS

Hydroxy-HIF2α-P-OH 531: FNELDLETLAP(OH)YIPMDGEDFQLS

### TFAM peptide hydroxylation and ^35^S-VHL capture

Peptide hydroxylation and ^35^S-VHL capture (Fig. 4d) were performed as described above^20^. In short, HA-EGLN1, HA-EGLN2, HA-EGLN3 AND HA-EGLN3 catalytically dead p.His196Arg mutant were synthesized by IVT using TnT T7 Quick Master Mix. Naïve biotin-TFAM peptide (1 μg) was conjugated with Streptavidin agarose beads (GE Healthcare Life Sciences) in 1 ml PBS at room temperature. The beads pellet was washed twice with PBS and once with hydroxylation buffer (40 mM HEPES, pH 7.4, 80 mM KCl) and resuspended with 300 μl hydroxylation reaction buffer. A total of 15 μl of IVT-synthesized HA-EGLN was added to the hydroxylation reaction and rotated for 2 h. Then, 500 μl EBC buffer and 15 μl IVT-synthesized S^35^ radioactive-labelled HA-VHL was added and incubated overnight at 4 °C. Samples were centrifuged and washed five times with immunoprecipitation wash buffer. Bound peptide/protein complexes were eluted with 30 μl Laemmli buffer, boiled and centrifuged. Bound ^35^S-HA-VHL was eluted by boiling in SDS-containing sample buffer, resolved by PAGE and detected by autoradiography.

### Mass spectrometry analysis for peptide hydroxylation

The hydroxylation assay with TFAM peptide and EGLN3 (Fig. 4c–f) was performed as described above and processed as recently described^53^. In short, after hydroxylation assay, beads conjugated to TFAM peptide were washed once with hydroxylation buffer and three times with IP buffer without detergent. Peptides were digested with trypsin and directly analysed by MS on a Q-Exactive mass spectrometer connected to an UltiMate 3000 chromatography system as recently described^20^.

### S^35^VHL-mutant capture with hydroxylated hydroxy-TFAM-P-OH-53/66

The following biotinylated peptides were used for ^35^S-VHL-mutant capture (Fig. 4h,i and Extended Data Fig. 4h):

hydroxy-TFAM-P-OH-53/66, hydroxy-HIF1α-P-OH 564 and hydroxy-HIF2α-P-OH 531.

Peptides were rotated for 1 h at room temperature and samples were subsequently washed twice with PBS. ^35^S-VHL produced by IVT was captured as previously described^20^ and above.

### HA-VHL pulldown using hydroxylated peptides in ccRCC

HA-VHL pulldown in ccRCC cells (Fig. 4j and Extended Data Fig. 3) was performed with the following biotinylated peptides: naïve TFAM and naïve HIF2α as control, hydroxy-TFAM-P-OH-53/66 and hydroxy-HIF2α-P-OH. First, peptides were conjugated with streptavidin beads and incubated with cell lysates for 4 h at 4 °C with rotation. Samples were then washed four times with immunoprecipitation washing buffer (0.5% NP-40, 150 mM NaCl and 10 mM Tris-HCl) and eluted with 30 μl Laemmli buffer, boiled for 5 min and centrifuged at 8,000*g* for 30 s. The resulting supernatant was subjected to immunoblot analysis.

### TFAM degradation assay by LONP1

In total, 60 μl Dynabeads His-Tag Isolation & Pulldown (10103D) was incubated with purified His-TFAM (2 μM) for 1 h 30 min at room temperature with rotation. Meanwhile, Flag-EGLN3 WT and Flag-*EGLN3*-p.His196Arg catalytic dead mutant were synthesized by IVT as described above. His-TFAM conjugated beads were resuspended with 1 ml hydroxylation reaction buffer supplemented with 100 μM iron chloride, 2 mM ascorbate and 5 mM 2-oxolgutarate. Then, 75 μl unprogrammed reticulocyte lysate, IVT-synthesized wild-type EGLN3 or EGLN3-p.His196Arg mutant was added to start the hydroxylation reaction. The hydroxylation reaction was processed for 2 h at room temperature with rotation. Next, 1 μg HA-VHL was incubated with the reaction samples for 2 h at room temperature with rotation and the bound protein complexes were washed twice with distilled water and resuspended with 25 μl LONP1 degradation buffer containing 30 mM NaCl, 10 mM HEPES-potassium hydroxide buffer (pH 8.0), 2 mM magnesium chloride, 0.1 mg ml^−1^ BSA, 4 mM ATP and 100 nM LONP1. The TFAM degradation assay by LONP1 was processed for 2 h at 37 °C. Bound protein complexes were eluted with 30 μl of Laemmli buffer, boiled for 5 min and centrifuged at 8,000*g* for 30 s. Eluted supernatant was analysed by immunoblotting.

### Expression plasmids, shRNA, siRNAs and gRNA

pcDNA3 Flag-*EGLN3*, Flag-p.His196Arg mutant and pcDNA3-*VHL* including *VHL*-missense mutations have been described previously^54^. Lentiviruses encoding FLAG-*EGLN3* and FLAG-*EGLN3-*p.His196Arg were generated in 293FT cells (Thermo Fisher, R70007) as previously described^48^. siRNAs targeting *Egln1*, *Egln2* or *Egln3* were generated with the following sequences: si*EGLN1*: (5′->3′): (AGCUCCUUCUACUGCUGCA)(UU); si*EGLN2*: (5′->3′): (GCCACUCUUUGACC-GGUUGCU)(UU); si*EGLN3*: (5′->3′): (CAGGUUAUGUUCGCCAC-GU)(UU). Lentiviruses encoding shRNAs targeting human *EGLN1*, *EGLN2* and *EGLN3* were generated using the pLKO.1 plasmid using the following sequences:

*EGLN1* (5′-CCGGGACGACCTGATACGCCACTGTCTCGAGACAGGGCGTATCAGG-TCGTCTTTTT-3′);

*EGLN2* (5′-CCGGCTGGGACGTTAAGGTGCATGGCTCGAGCCATGCACCTTAACG-TCCCAGTTTTT-3′);

*EGLN3* (5′-CCGGGTTCTTCTGGTCAGATCGTAGCTCGAGCTACGATCTGACCAGA-AGAACTTTTTG-3′).

sgRNA (Sigma-Aldrich) sequences targeting *EPAS1* or control were cloned into pLentiCRISPR-V2 (Addgene no. 52961, provided by the laboratory of J.H.).

sgEPAS1-EX2-T1-SS: 5ʹ-caccgGTGCCGGCGGAGCAAGGAGA-3ʹ,

sgEPAS1-EX2-T1-AS: 5ʹ-aaacTCTCCTTGCTCCGCCGGCACc-3ʹ;

sgEPAS1-EX2-T2-SS: 5ʹ-caccgGATTGCCAGTCGCATGATGG-3ʹ,

sgEPAS1-EX2-T2-AS: 5ʹ-aaacCCATCATGCGACTGGCAATCc-3ʹ,

sgCONTROL-SS: 5ʹ-caccgCTTGTTGCGTATACGAGACT-3ʹ,

sgCONTROL-AS: 5ʹ-aaacAGTCTCGTATACGCAACAAGc-3ʹ.

### Generation of piggyBac vector expressing wild-type and mutant *TFAM*

*TFAM* wild-type and P53/66A mutant ORF CDS were PCR from pReciver-M07 (GeneCopoeia, EX-F0074-M09). Primers included were iTFAM-F:

5ʹ-GAATGGTCTCTCTAGCGCCGCCACCATGGCGTTTCTCCGAAGC-3ʹ;

iTFAM-R: 5ʹ-GAATGGTCTCTCGCGTTCACAGATCCTCTTCAGAGATGAGT-3ʹ (Integrated DNA Technologies). The PCR products and pB-TRE-empty or pB-TRE-Luc2-empty vectors (gifts from the laboratory of J.H.) were digested by NheI and MluI (New England Biolabs). After purification, the PCR products were ligated into the pB-TRE-empty or pB-TRE-Luc2-empty vector. Sequences were confirmed using the Sanger method (Integrated DNA Technologies).

### Generation of stable and inducible *TFAM* expression in HEK293 cells

Transposon vectors pB-TRE-*TFAM*-wt-Luc2, pB-TRE-*TFAM*-mut-Luc2 and transposase vector pCAG-hyPBase were transfected at the ratio of 4:1 into HEK293 cells by Lipofectamine 2000 (Invitrogen) according to the manufacturer’s instructions. Two days later, cells were selected under 200 µg ml^−1^ hygromycin B until the blank cells were 100% dead. Then 0.9 × 10^5^ cells were seeded into six-well dishes, and 100 ng ml^−1^ doxycycline (Clontech) was added into the cells for 48 h before the cells were harvested for western blot.

### Co-immunoprecipitation

One confluent p150 plate of 786-O cells stably expressing *VHL* (pPC3, WT-*VHL*, p.Leu188Val*-VHL*) or 786-O WT-*VHL* cells infected with lentivirus targeting *Egln1*, *Egln2* and *EGLN3*, respectively, was washed once in ice-cold PBS, trypsinized and subsequently harvested with 10 ml ice-cold PBS, collected by centrifugation (5 min at 1,200*g*, 4 °C), resuspended and homogenized in EBC buffer (50 mM Tris at pH 8.0, 120 mM NaCl, 0.5% NP-40) containing protease inhibitor and phosphatase inhibitor. The lysates were centrifuged at 14,000*g* for 10 min to pellet unlysed cellular debris and the resulting supernatant was incubated with HA antibody overnight at 4 °C. Samples were incubated for 5 h with rotation at 4 °C with 70 μl (50% slurry) Protein G agarose beads (sc-2002, Santa Cruz) were pre-washed twice with immunoprecipitation washing buffer (0.5% NP-40, 150 mM NaCl, 10 mM Tris-HCl). Samples were centrifuged at 8,000*g* for 30 s and washed five times with immunoprecipitation washing buffer (0.5% NP-40, 150 mM NaCl, 10 mM Tris-HCl). Immunoprecipitated proteins were eluted with 50 μl Laemmli buffer, boiled for 5 min and centrifuged at 8,000*g* for 1 min. The resulting eluted supernatant was subjected to immunoblot analysis.

### Co-immunoprecipitation using anti-hydroxyproline antibody

293FT cells were transiently transfected with plasmids encoding Flag-*TFAM* and Flag-*EGLN3* WT or catalytically dead mutant (Mut) with or without DMOG treatment or transiently transfected with plasmids encoding Flag-TFAM and HA-*Egln1*, HA-*Egln2* and HA-*EGLN3*. Immunoprecipitation using anti-hydroxyproline antibody (HydroxyP; ab37067, GR3215743-1 and GR3179915-1) from 293FT cells (Fig. 4b and Extended Data Fig. 4a) was performed as described above.

### Protein kinase A activity assay

Hydroxylated TFAM (1 μg) biotinylated peptide was conjugated with 30 μl Streptavidin agarose beads (GE Healthcare Life Sciences) in 1 ml PBS at room temperature with rotation for 1 h. The beads pellet was washed three times with PBS and resuspended in 500 μl PBS supplemented with 1 μg purified GST-pVHL protein for 2 h at room temperature. Subsequently, TFAM phosphorylation by PKA was assayed using PKA Kinase Activity Assay Kit (ab139435, Abcam) according to the manufacturer’s instructions. Samples were centrifuged at 8,000*g* for 30 s and washed three times with PBS and resuspended with 50 μl kinase assay dilution buffer supplemented with 1 μg μl^−1^ ATP and 50 ng purified active PKA. The phosphorylation assay was processed for 40 min at room temperature with rotation and for 40 min at 30 °C. Samples were centrifuged at 8,000*g* for 30 s and washed three times with PBS and eluted with 30 μl Laemmli buffer, boiled for 5 min and centrifuged at 8,000*g* for 30 s. Eluted supernatant was analysed by immunoblotting using pan-phospho-serine antibody (Santa Cruz, sc-81514).

### Crystal violet staining for apoptosis

ccRCC 786-O cells and MEFs were treated with 3-BP or gossypol for the indicated times. Cells were washed once with PBS and then fixed and stained by crystal violet solution (0.1% crystal violet, 20% methanol, 80% dH_2_O) for 45 min at room temperature and washed four times with PBS.

### TFAM half-life

786-O cells and MEFs were cultured in six-well plates in 2 ml DMEM medium to reach 50% confluency. Cells were treated with 10 μM cycloheximide at the indicated times, subsequently harvested and lysed in EBC lysis buffer. Cell lysates were then subjected to western blot analysis with the rabbit anti-TFAM antibody.

### Evaluation of mitochondrial respiration rate and extracellular acidification rate

The OCR and ECAR were determined using Mito Stress Test Kit (Agilent,103015–100), Glycolysis Stress Test Kit (Agilent, 103020-100) and an XFe96 Extracellular Flux Analyzer (Seahorse Bioscience). Then, the XFe96 Sensor Cartridge (102416-100) was hydrated using 200 μl of sterile calibrant (100840-000) in each well of the utility plate. The assembled sensor cartridge and utility plate were kept in a 37 °C CO_2_-free incubator overnight. 786-O cells and MEFs were seeded into XFe96 cell culture microplates (101085-004) at a density of 10,000 cells per well and allowed to adhere to plate overnight. The cell culture medium was replaced with Seahorse XF DMEM medium (103575-100) containing 10 mM glucose, 2 mM glutamine, 1 mM pyruvate and placed in a 37 °C CO_2_-free incubator for 1 h. Finally, after preincubation, OCR was measured in the Agilent’s Seahorse Bioscience XF-96 Extracellular Flux Analyzer (Agilent Technologies) from the baseline OCR determination and subsequent sequential injections of three compounds that affect the cellular bioenergetic processes, as follows: 20 μl of oligomycin (10 μM) in port A, 22 μl of FCCP (10 μM) in port B and 25 μl of rotenone/antimycin A (5 μM) in port C, according to the manufacturer’s instructions and protocols. ECAR was measured from the baseline ECAR determination and subsequent sequential injections of three compounds as follows: 20 μl of glucose (100 mM) in port A, 22 μl of oligomycin (10 μM) in port B and 25 μl of 2-deoxy-d-glucose (500 μM) in port C, according to the manufacturer’s instructions and protocols.

### Proximity ligation assay, image acquisition and image processing

786-0 cells (pRC3 and *VHL* WT) were plated on glass coverslips. Cells were incubated with 100 nM MitoTracker Red CMXRos (Invitrogen) for 1 h at 37 °C and fixed with 4% paraformaldehyde and permeabilized using 0.1% Triton X-100 (Sigma-Aldrich) in PBS. After incubation with TFAM and VHL antibodies (1:500 dilution; 8076, Cell Signalling Technology; and 1:1,000 dilution; 564183, Becton Dickinson, respectively) at 4 °C overnight, the PLA assay was performed using Duolink In Situ PLA Probe Anti-Rabbit PLUS and Anti-Mouse MINUS, and Duolink In Situ Detection Reagents Green (Sigma-Aldrich) following the manufacturer’s instructions. The immunofluorescence signals were acquired by an LSM 700 Laser Scanning Confocal System with Zeiss Observer Z1 Inverted Phase Contrast Fluorescence Microscope (ZEISS) using ×63 magnification. Twenty images of randomly selected areas per cell line were taken. Each fluorophore channel was pseudo-coloured in ZEN2 (ZEISS), exported as JPEG, and analysed using the CellProfiler 4.2.0 cell image analysis software (Broad Institute of Harvard and MIT). The number of PLA signals per cell was quantified from the maximal intensity projection of each image. Statistical analysis was performed using Prism 9 software to calculate the non-parametric Mann–Whitney *U* test (GraphPad). A *P* value < 0.05 was considered significant.

### Mitochondrial fractionation

The mitochondria were isolated as previously described^55^. The cells were collected and resuspended in the MSE buffer (109 mg ml^−1^ mannitol (M4125, Sigma), 10 mM Tris pH 7.4, 1 mM EDTA) containing 0.1% BSA (A7030, Sigma). The cells were homogenized followed by low-speed centrifugation to remove the cell debris. Mitochondria were pelleted and washed with MSE buffer. One-third of the mitochondrial fraction was pelleted as ‘untreated mitochondrial fraction’. Two-thirds of the mitochondria were digested with 15 µg ml^−1^ proteinase K (25530049, Thermo Fisher); half of them were treated with 1 mM PMSF followed by washing twice with MSE buffer containing PMSF and the other half was treated with 1% Triton-X-100. The total cell and mitochondrial fractions were lysed in the lysis buffer (LAGIPT1, Sigma) containing protease inhibitor cocktail (Roche) before loading on the SDS–PAGE.

### Chemical reagents

Gossypol (G8761-100 mg), H89 dihydrochloride hydrate (B1427), 3-BP and cycloheximide (C4859) were purchased from Sigma-Aldrich. Bortezomib (B-1408) was purchased from LC Laboratories. MitoTracker Red CMXRos (M7512) and MitoTracker Green FM (M7514) were purchased from Thermo Fisher Scientific. Forskolin (3828) was purchased from Cell Signaling Technology.

### Quantification and statistical analysis

The hypothesis that the percentage of mitochondrial proteins was significantly lower in both *VHL*-null and *VHL*^*L188V*^ cells compared to *VHL* wild-type-expressing cells was tested. We tested the null hypothesis that there is no difference between the proportion of mitochondrial proteins with significantly higher and significantly lower abundances between the study cases and control (for example, wild type), using two-tailed Fisher’s exact tests. The null hypothesis was rejected using a *P*-value threshold of 0.01. Further, we tested the hypothesis that (1) the proportion of HIF-independent (for example, p.Leu188Val) and not-HIF-independent (for example, *VHL*-null excluding intersecting *VHL*^*L188V*^) significantly downregulated mitochondrial proteins, and (2) the proportion of HIF-independent (for example, *VHL* mutated PPGL intersecting *VHL*^*L188V*^) and not-HIF-independent (for example, *VHL* mutated PPGL excluding intersecting *VHL*^*L188V*^) significantly downregulated mitochondrial proteins, are not different than the same proportions in significantly downregulated non-mitochondrial proteins. These were analysed using two-tailed Fisher’s exact tests and were rejected using a *P*-value threshold of 0.01. Fisher’s tests were conducted using the Scipy package version 1.0.0, in Python 2.7.

Analysis of the quantitative proteomics data was performed as previously described. Briefly, TMT reporter was used for MS-based peptide quantification. TMT reporter abundance values were normalized to the total abundance of the same TMT channel. Quality check was performed by calculating the variation (CV) between the replicates as well as by building principal-component analysis models to verify the small data spread between the replicates. The median values of the replicates in each condition were used for fold change calculations. The significance of protein abundance difference between two different conditions was calculated by two-tailed unpaired *t*-test. Mitochondrial proteins were selected according to the cellular component GO term searched by Proteome Discoverer v2.3 in the human Swiss-Prot protein database.

### Reporting summary

Further information on research design is available in the Nature Research Reporting Summary linked to this article.

## Supplementary information

Supplementary Information Supplementary Figs. 1 and 2, including legends (FACS gating strategies and western blot quantification).

Reporting Summary

Supplementary Table 1 The cellular proteomes from primary PPGL tumours were extracted and analysed by nanoLC–MS/MS; 6,196 proteins were identified and quantified, 5,576 of which were common to all the samples.

Supplementary Table 2 List of mitochondrial proteins. Analysing the proteome of *VHL*-null and *VHL*^***L188V***^ cells confirmed that the percentage of mitochondrial proteins was significantly lower in both VHL-null cells as compared to *VHL* wild-type expressing cells.

## Source data

Source Data Fig. 1 Unprocessed western blots.

Source Data Fig. 1 Statistical source data.

Source Data Fig. 2 Unprocessed western blots.

Source Data Fig. 2 Statistical source data.

Source Data Fig. 3 Unprocessed western blots.

Source Data Fig. 3 Statistical source data.

Source Data Fig. 4 Unprocessed western blots.

Source Data Fig. 5 Unprocessed western blots.

Source Data Fig. 5 Statistical source data.

Source Data Fig. 6 Statistical source data.

Source Data Fig. 7 Unprocessed western blots.

Source Data Extended Data Fig. 1 Unprocessed western blots.

Source Data Extended Data Fig. 2 Unprocessed western blots.

Source Data Extended Data Fig. 2 Statistical source data.

Source Data Extended Data Fig. 3 Unprocessed western blots.

Source Data Extended Data Fig. 4 Unprocessed western blots.

Source Data Extended Data Fig. 4 Statistical source data.

Source Data Extended Data Fig. 5 Statistical source data.

Source Data Extended Data Fig. 6 Statistical source data.

Source Data Extended Data Fig. 7 Unprocessed western blots.

## Data Availability

Raw and analysed mass spectrometry data are available at https://www.ebi.ac.uk/pride/. Source data are provided with this paper. All other data associated with this study are presented in the paper or the extended data figures.
