# Publisher Correction: Impaired oxygen-sensitive regulation of mitochondrial biogenesis within the von Hippel–Lindau syndrome

**DOI:** 10.1038/s42255-022-00651-4

**Published:** 2022-09-08

**Authors:** Shuijie Li, Wenyu Li, Juan Yuan, Petra Bullova, Jieyu Wu, Xuepei Zhang, Yong Liu, Monika Plescher, Javier Rodriguez, Oscar C. Bedoya-Reina, Paulo R. Jannig, Paula Valente-Silva, Meng Yu, Marie Arsenian Henriksson, Roman A. Zubarev, Anna Smed-Sörensen, Carolyn K. Suzuki, Jorge L. Ruas, Johan Holmberg, Catharina Larsson, C. Christofer Juhlin, Alex von Kriegsheim, Yihai Cao, Susanne Schlisio

**Affiliations:** 1 Department of Microbiology, Tumor and Cell Biology, Karolinska Institutet, Stockholm, Sweden; 2 College of Pharmacy, Harbin Medical University, Harbin, China; 3 Department of Medical Biochemistry and Biophysics, Karolinska Institute, Stockholm, Sweden; 4 Edinburgh Cancer Research Centre, IGMM, University of Edinburgh, Edinburgh, UK; 5 Department of Physiology and Pharmacology, Karolinska Institutet, Stockholm, Sweden; 6 Department of Medicine, Karolinska University Hospital, Stockholm, Sweden; 7 Department of Microbiology, Biochemistry and Molecular Genetics, Rutgers University-New Jersey Medical School, Newark, NJ USA; 8 Department of Molecular Biology, Faculty of Medicine, Umeå University, Umeå, Sweden; 9 Department of Oncology-Pathology, Karolinska Institutet, Karolinska University Hospital, Stockholm, Sweden

**Keywords:** Cancer metabolism, Metabolism, Tumour-suppressor proteins, Mitochondria

Correction to: *Nature Metabolism* 10.1038/s42255-022-00593-x, published online 27 June 2022.

In the version of this article initially published, there were errors in the Fig. 4i,j right-hand lane labels, where “HIF2α-P-OH” originally appeared as “HIF1α-P-OH.” The labels have been corrected in the HTML and PDF versions of the article.

