# Supplementary material for: Impaired oxygen-sensitive regulation of mitochondrial biogenesis within the von Hippel-Lindau syndrome
Source: Nat Metab. Author manuscript; Available in PMC 2022 Jul 2. (PMC9236906; doi:10.1038/s42255-022-00593-x)
Supplement: Inventory_of_Supporting_Information [file EMS145242-supplement-Inventory_of_Supporting_Information.docx]

Inventory of Supporting Information

**Manuscript #: NATMETAB-A21014222D**.

**Corresponding author name(s):** Shuijie Li & Susanne Schlisio.

| Please complete each of the Inventory Tables below to outline your Extended Data and Supplementary Information items.  There are four sections:   - *Extended Data* - *Supplementary Information: Flat Files* - *Supplementary Information: Additional Files* - *Source Data*   Each section includes specific instructions. Please complete these tables as fully as possible. We ask that you avoid using spaces in your file names, and instead use underscores, i.e.: Smith_ED_Fig1.jpg not Smith ED Fig1.jpg  Please note that titles and descriptive captions will only be lightly edited, so please ensure that you are satisfied with these prior to submission.  If you have any questions about any of the information contained in this inventory, please contact the journal. |
| --- |
|  |

1. **Extended Data**

**Complete the Inventory below for all Extended Data figures.**

- Keep Figure Titles to one sentence only
- Upload your files as ‘Figure Files’ in our Manuscript Tracking system
- File names should include the Figure Number. i.e.: *Smith_ED_Fig1.jpg*
- Please be sure to include the file extension in the Filename. Note that Extended Data files must be submitted as .jpg, .tif or .eps files *only*, and should be approximately 10MB
- All Extended Data figure legends must be provided in the Inventory below and should not exceed 300 words each *(if possible)*
- Please include Extended Data *ONLY* in this table

| Figure # | Figure title  One sentence only | Filename  This should be the name the file is saved as when it is uploaded to our system. Please include the file extension. i.e.: *Smith_ED_Fig1.jpg* | Figure Legend  If you are citing a reference for the first time in these legends, please include all new references in the main text Methods References section, and carry on the numbering from the main References section of the paper. If your paper does not have a Methods section, include all new references at the end of the main Reference list. |
| --- | --- | --- | --- |
| Extended Data Fig. 1 | VHL regulates of mitochondrial mass independent of HIFa. | Extended_Data_Fig1.jpg | (a) List human primary PPGL tumors with characterized mutation status and 1p36 status that were analyzed by by nanoLC-MS/MS in Figure 1A-D. wt = wild-type. (b) Heatmap of significantly regulated mitochondrial proteins in VHL-mutant compared to VHL wild-type PPGL tumors (p < 0.05, two-tailed unpaired t test). (c) Top 5 cellular component of top 50 up (red)- and down (green)-regulated proteins for human VHL mutant PCC/PGL tumors compared to VHL wild type PCC/PGL tumors according to the false discovery rate (FDR). Medium confidence threshold (0.4) was used to define protein-protein interactions. (d) Immunoblot analysis of A498 VHL-null cells (−/−) stably transfected to generate HA-VHL (WT). n = 3 biological independent experiments. (e) Immunoblot analysis of 786-O cells with indicated genotype stably transduced with lentivirus encoding sgRNA targeting HIF2α. n = 3 biological independent experiments. (f) Venn diagram representing significantly downregulated proteins shared in VHL-null 786-O cells with type 2C VHL-L188V mutant cells and (g) shared with VHL mutant PPGL. (h, i) GO term enrichment in cellular component of 393 significantly down-regulated proteins (p values < 0.0001，two-tailed unpaired t test) comparing VHL-null to VHL-WT cells (h) and 200 significantly down-regulated proteins (p values < 0.0001，two-tailed unpaired t test) comparing VHL-L188V to VHL-WT cells (i) performed using DAVID and plotted using REVIGO. The size of the bubbles is indicative of the number of proteins annotated with that GO term; bubbles are color coded according to significance. |
| Extended Data Fig. 2 | VHL regulation of mitochondrial mass is hydroxylation and EglN3 dependent. | Extended_Data_Fig2.jpg | (a) Immunoblot analysis of 786-O cells with indicated VHL status transduced with lentiviral pL.KO shRNA targeting EGLN3 (shE3) or no targeting control (SCR). n = 3 biological independent experiments. (b) Immunoblot analysis of HeLa cells transduced with lentiviral pL.KO shRNA targeting EGLN1, EGLN2, EGLN3 or no targeting control. n = 3 biological independent experiments. (c) Immunoblot analysis of mouse cerebellum of indicated genotype. n = 4 biologically independent EGLN3 wildtype or knockout mice. (d) Immunoblot analysis of mouse skeletal muscles of indicated genotype. n = 3 biologically independent EGLN3 wildtype or knockout mice. (e) Immunoblot of primary EglN3-MEFs of indicated genotype with different passages. n = 3 biological independent experiments. (f) Immunoblot analysis of primary EGLN3-MEFs of indicated genotype. n = 3 biological independent experiments. (g) Left: Fluorescence images of primary EGLN3-MEFs of indicated genotype. Mitochondria were stained by MitoTracker Red. Right: Flow cytometry analysis of MitoTracker Green-stained primary MEFs of indicated genotype. Data are presented as mean values ± S.D. Two-tailed unpaired t test. ****p <0.0001. n = 3 biological independent experiments. (h) Immunoblot of EGLN3 primary MEFs with indicated genotype upon normoxic or anoxic conditions for 16h or treated with 1 mM DMOG or 50 μM FG0041 for 8 h. n = 3 biological independent experiments. (i) In contrast young adult, KO mice (18-19 weeks of age) show a comparable exhaustion time, performed work and performed power (n=16 per genotype, male mice). Data represent means ± SD and individual measurements. |
| Extended Data Fig. 3 | VHL interacts with TFAM within mitochondria. | Extended_Data_Fig3.jpg | (a) Immunoblot analysis of subcellular fractionation of SK-N-F1 cells. Cell lysates were fractionated into cytosolic and mitochondrial fractions. In addition, aliquots of the mitochondrial fractions were treated with 25 μg/ml Proteinase K with or without treatment with 1% Triton X-100. Fractions were analyzed by western blotting and the localization of VHL or EglN3 was assessed in comparison to that of protein markers of the cytosol (tubulin), outer mitochondrial membrane (TOM20), and mitochondrial matrix (mitochondrial ribosomal protein MRPL37). n = 3 biological independent experiments. (b) Representative images of proximity ligation assay (PLA) signal (green), DAPI (blue) and MitoTracker Red (red) triple staining in 786-0 cells expressing VHL wildtype. The images show the maximal intensity projection of the signal/staining. (c) 3D rendering and (d) Orthogonal view showing co-localization of PLA signal in mitochondria (yellow). Magnification 63x; scale bar: 5 µm. (b-d) Similar results were seen more than three times. |
| Extended Data Fig. 4 | TFAM is hydroxylated by EglN3 at Proline 53/66 causing pVHL recognition. | Extended_Data_Fig4.jpg | (a) Immunoprecipitation using antihydroxyproline antibody (HydroxyP) from 293FT cells that were transiently transfected with plasmids encoding Flag-TFAM and HA-EGLN1, HA-EGLN2 and HA-EGLN3. Immunoblots show co-immunoprecipitation of Flag-TFAM. n = 3 biological independent experiments. (b-c) Mass spectrometry of unmodified biotinylated TFAM-peptide-30-70. Shown is the representative fragmentation peptide spectra of non-hydroxylated Biotin-KPVSSYLR (b) and non-hydroxylated Biotin-EQLPIFKA (c). (d,e) Extracted ion chromatogram of biotinylated unmodified and mono hydroxylated proline residues 53 (d) or proline residues 66 (e) TFAM peptide following an in vitro hydroxylation reaction with EglN3 with indicated concentration of α-ketoglutarate (KG). Control indicates unmodified biotinylated TFAM-peptide that was not subjected to EGLN3 hydroxylation. (f) Hydroxylation levels of proline residues 53 and 66 of TFAM peptide following hydroxylation with EGLN3 generated via IVT with indicated concentration of KG. Data are presented as mean values ± SD. n = 3 biological experiments. One way ANOVA Tukey's Multiple Comparison Test. *p <0.05 ,**p <0.01. p=0.0288, p=0.0143, p=0.0148, p=0.0082. (g) Schematic illustration of synthetic biotinylated TFAM peptide hydroxylated at P-OH-53 and P-OH-66 and naïve TFAM peptide. (h) Autoradiograms showing recovery of 35S-labeled VHL protein (WT) or corresponding disease mutants (as indicated) bound to biotinylated HIF1α peptide (residues 556 to 575) with hydroxylated proline 564 (HIF1α-P-OH) and HIF2α peptide (residues 521 to 543) with hydroxylated proline 531 (HIF2α-P-OH). Biotinylated HIF1α and HIF2α naïve peptides were used as negative controls. n = 3 biological independent experiments. (i) Peptide pulldown using biotinylated TFAM-P-OH-53/66 peptide incubated with whole-cell lysates from A498 cells expressing HA-VHL WT or empty control. Biotinylated TFAM naïve peptide was used as negative control. n = 3 biological independent experiments. |
| Extended Data Fig. 5 | VHL restores cellular oxygen consumption rate. | Extended_Data_Fig5.jpg | (a) Seahorse XF-96 analysis of oxygen consumption rate (OCR). Mitochondrial respiration reflected by OCR levels was detected in 786-O cells with indicated genotype. The rates of basal respiration and maximal respiratory capacity were respectively quantified by normalization of amount of cells. One way ANOVA Tukey's Multiple Comparison Test. ****p <0.0001. (b) Seahorse XF-96 analysis of oxygen consumption rate (OCR) of 786-O cells with indicated VHL status transduced with lentiviral pL.KO shRNA targeting EGLN3 or no targeting control. The rates of basal respiration and maximal respiratory capacity were respectively quantified as described above. One way ANOVA Tukey's Multiple Comparison Test. ****p <0.0001. (c) Seahorse XF-96 analysis of oxygen consumption rate (OCR) of primary EGLN3+/+ and EGLN3-/- MEFs. The rates of basal respiration and maximal respiratory capacity were respectively quantified by normalization of amount of cells. One way ANOVA Tukey's Multiple Comparison Test. ***p <0.001, ****p <0.0001. (d) Seahorse XF-96 analysis of oxygen consumption rate (OCR) of primary EGLN3-MEFs of indicated genotype stably transduced with lentivirus encoding EGLN3 WT, catalytic death mutant or empty control. The rates of basal respiration and maximal respiratory capacity were respectively quantified as described above. ***p <0.001, ****p <0.0001. a-d, data are presented as mean values ± SD. n = 3 biological independent experiments. (e) Crystal violet staining of 786-O cells with indicated VHL status treated with high glucose (25 mM) or no glucose respectively for 36 hours. (f) Crystal violet staining of primary EGLN3+/+ and EGLN3-/- MEFs treated with 100 μM 3-bromopyruvic acid (3-BP) for 4 hours. (g) Crystal violet staining of primary EGLN3+/+ and EGLN3-/- MEFs treated with high glucose (25μM) or no glucose (0μM) respectively for 48 hours. (h) Crystal violet staining of 786-O cells with indicated VHL status transduced with lentiviral pL.KO shRNA targeting EGLN3 or no targeting control, treated with 100 μM 3-bromopyruvic acid (3-BP) for 4 hours. |
| Extended Data Fig. 6 | VHL decreases glycolysis. | Extended_Data_Fig6.jpg | (a) Extracellular acidification rate (ECAR) of 786-O cells with indicated genotype was monitored using the Seahorse XF-96 Extracellular Flux Analyzer with the sequential injection of glucose (10 mM), oligomycin (1 μM) and 2-deoxy-glucose (2-DG) (50 μM). The rates of glycolysis and glycolysis capacity were respectively quantified by normalization of amount of cells. One way ANOVA Tukey's Multiple Comparison Test. **p =0.003, ***p =0.0002, ****p <0.0001. (b) Extracellular acidification rate (ECAR) of 786-O cells with indicated VHL status transduced with lentiviral pL.KO shRNA targeting EGLN3 or no targeting control was measured as described above. The rates of glycolysis and glycolysis capacity were respectively quantified by normalization of amount of cells. One way ANOVA Tukey's Multiple Comparison Test. ****p <0.0001. (c) Extracellular acidification rate (ECAR) of primary EGLN3+/+ and EGLN3-/- MEFs. The rates of glycolysis and glycolysis capacity were respectively quantified by normalization of amount of cells. One way ANOVA Tukey's Multiple Comparison Test. ****p <0.0001. (d) Extracellular acidification rate (ECAR) of primary EGLN3-MEFs of indicated genotype stably transduced with lentivirus encoding EGLN3 WT, catalytic death mutant or empty control was monitored as described above. The rates of glycolysis and glycolysis capacity were respectively quantified by normalization of amount of cells. One way ANOVA Tukey's Multiple Comparison Test. ****p <0.0001. a-d, data are presented as mean values ± SD. n = 3 biological independent experiments. |
| Extended Data Fig. 7 | Low mitochondrial content in pheochromocytoma cells causes impaired differentiation. | Extended_Data_Fig7.jpg | (a) Immunoblot analysis of stable polyclonal PC12 cells expressing the indicated human VHL (huVHL) species. Stable polyclonal PC12 cells were transduced for 48 h with lentivirus encoding shRNA targeting endogenous rat VHL (endg. sh-ratVHL) or scramble control (shSCR) and subsequently treated with NGF for 6 days. n = 3 biological independent experiments. |
| Extended Data Fig. 8 |  |  |  |
| Extended Data Fig. 9 |  |  |  |
| Extended Data Fig. 10 |  |  |  |

***Delete rows as needed to accommodate the number of figures (10 is the maximum allowed).***

1. **Supplementary Information:**
2. **Flat Files**

**Complete the Inventory below for all additional textual information and any additional Supplementary Figures, which should be supplied in one combined PDF file.**

- **Row 1:** A combined, flat PDF containing any Supplementary Text, Discussion, Notes, Additional Supplementary Figures, Supplementary Protocols, simple tables, and all associated legends. Only one such file is permitted.
- **Row 2:** Nature Research’s Reporting Summary; if previously requested by the editor, please provide an updated Summary, fully completed, without any mark-ups or comments. **(Reporting Summaries are not required for all manuscripts.)**

| Item | Present? | Filename  This should be the name the file is saved as when it is uploaded to our system, and should include the file extension. The extension must be .pdf | A brief, numerical description of file contents.  i.e.: *Supplementary Figures 1-4, Supplementary Discussion, and Supplementary Tables 1-4.* |
| --- | --- | --- | --- |
| Supplementary Information | Yes | Supplementary Information.pdf | *Supplementary Figures 1-2, including Supplementary legend of Supplementary Figures 1-2*  (FACS gating strategies and WB quantification) |
| Reporting Summary | Yes | Reporting Summary |  |
| Peer Review Information | Choose an item. | *OFFICE USE ONLY* |  |

1. **Additional Supplementary Files**

**Complete the Inventory below for all additional Supplementary Files that cannot be submitted as part of the Combined PDF.**

- Do not list Supplementary Figures in this table (see section 2A)
- Where possible, include the title and description within the file itself
- Spreadsheet-based tables & data should be combined into a workbook with multiple tabs, not submitted as individual files.
- Compressed files are acceptable where necessary. ZIP files are preferred.
- Please note that the *ONLY* allowable types of additional Supplementary Files are:

| - Supplementary Tables | - Supplementary Audio | - Supplementary Videos | - Supplementary Software |
| --- | --- | --- | --- |
| - Supplementary Data, for example: raw NMR Data, Cryo-EM Data, Computational Data, Crystallographic Data, etc. | | | |

| Type | Number  If there are multiple files of the same type this should be the numerical indicator. i.e. “1” for Video 1, “2” for Video 2, etc. | Filename  This should be the name the file is saved as when it is uploaded to our system, and should include the file extension. i.e.: *Smith_ Supplementary_Video_1.mov* | Legend or Descriptive Caption  Describe the contents of the file |
| --- | --- | --- | --- |
| Supplementary Table | Supplementary Table S1 | Data_set_1.xlsx | The cellular proteomes from primary PPGL tumors were extracted and analyzed by nanoLC-MS/MS. 6,196 proteins were identified and quantified, 5,576 of which were common to all the samples |
| Supplementary Table | Supplementary Table S1 | Data_set_2.xlsx | List of mitochondrial proteins: Analyzing the proteome of *VHL*-null and *VHL****^L188V^*** cells confirmed that the percentage of mitochondrial proteins was significantly lower in both VHL null cells as compared to *VHL* wild-type expressing cells |
| Choose an item. |  |  |  |
| Choose an item. |  |  |  |
| Choose an item. |  |  |  |
| Choose an item. |  |  |  |

***Add rows as needed to accommodate the number of files.***

1. **Source Data**

**Complete the Inventory below for all Source Data files.**

- Acceptable types of Source Data for Main Figures and Extended Data Figures are:
  - Statistical Source Data
    - Plain Text (ASCII, TXT) or Excel formats only
    - One file for each relevant Figure, containing all source data
  - Full-length, unprocessed Gels or Blots
    - JPG, TIF, or PDF formats only
    - One file for each relevant Figure, containing all supporting blots and/or gels
- ‘Source Data’ is only allowed for Main Figures and Extended Data Figures.
  - Include Unprocessed Gels or Blots for Supplementary Figures as additional Supplementary Figures.
  - Include Statistical Source Data for Supplementary Figures as ‘Supplementary Data’ files and list them in section 2B.
  - Please see [this example of Source Data](https://www.nature.com/articles/s41591-019-0505-4) in a publication.

| Parent Figure or Table | Filename  This should be the name the file is saved as when it is uploaded to our system, and should include the file extension. i.e.: *Smith_SourceData_Fig1.xls,* or *Smith_ Unmodified_Gels_Fig1.pdf* | Data description  i.e.: Unprocessed Western Blots and/or gels, Statistical Source Data, etc. |
| --- | --- | --- |
| Source Data Fig. 1 | Unprocessed_western_blots_Fig1.pdf,  Statistical_SourceData_Fig1.xlsx | Unprocessed western blots,  Statistical Source Data |
| Source Data Fig. 2 | Unprocessed_western_blots_Fig2.pdf,  Statistical_SourceData_Fig2.xlsx | Unprocessed western blots,  Statistical Source Data |
| Source Data Fig. 3 | Unprocessed_western_blots_Fig3.pdf,  Statistical_SourceData_Fig3.xlsx | Unprocessed western blots,  Statistical Source Data |
| Source Data Fig. 4 | Unprocessed_western_blots_Fig4.pdf | Unprocessed western blots |
| Source Data Fig. 5 | Unprocessed_western_blots_Fig5.pdf,  Statistical_SourceData_Fig5.xlsx | Unprocessed western blots,  Statistical Source Data |
| Source Data Fig. 6 | Statistical_SourceData_Fig6.xlsx | Statistical Source Data |
| Source Data Fig. 7 | Unprocessed_western_blots_Fig7.pdf | Unprocessed western blots |
| Source Data Fig. 8 |  |  |
| Source Data Extended Data Fig. 1 | Unprocessed_western_blots_EDFig1.pdf | Unprocessed western blots |
| Source Data Extended Data Fig. 2 | Unprocessed_western_blots_EDFig2.pdf,  Statistical_SourceData_EDFig2.xlsx | Unprocessed western blots,  Statistical Source Data |
| Source Data Extended Data Fig. 3 | Unprocessed_western_blots_EDFig3 | Unprocessed western blots |
| Source Data Extended Data Fig. 4 | Unprocessed_western_blots_EDFig4.pdf,  Statistical_SourceData_EDFig4.xlsx | Unprocessed western blots,  Statistical Source Data |
| Source Data Extended Data Fig. 5 | Statistical_SourceData_EDFig5.xlsx | Statistical Source Data |
| Source Data Extended Data Fig. 6 | Statistical_SourceData_EDFig6.xlsx | Statistical Source Data |
| Source Data Extended Data Fig. 7 | Unprocessed_western_blots_EDFig7.pdf | Unprocessed western blots |
| Source Data Extended Data Fig. 8 |  |  |
| Source Data Extended Data Fig. 9 |  |  |
| Source Data Extended Data Fig. 10 |  |  |
