# Supplementary figures and images for: Impaired oxygen-sensitive regulation of mitochondrial biogenesis within the von Hippel-Lindau syndrome
Source: Nat Metab. Author manuscript; Available in PMC 2022 Jul 2. (PMC9236906; doi:10.1038/s42255-022-00593-x)

Extended Data Fig. 1

d  
(flipped horizontally for Fig. S1d)

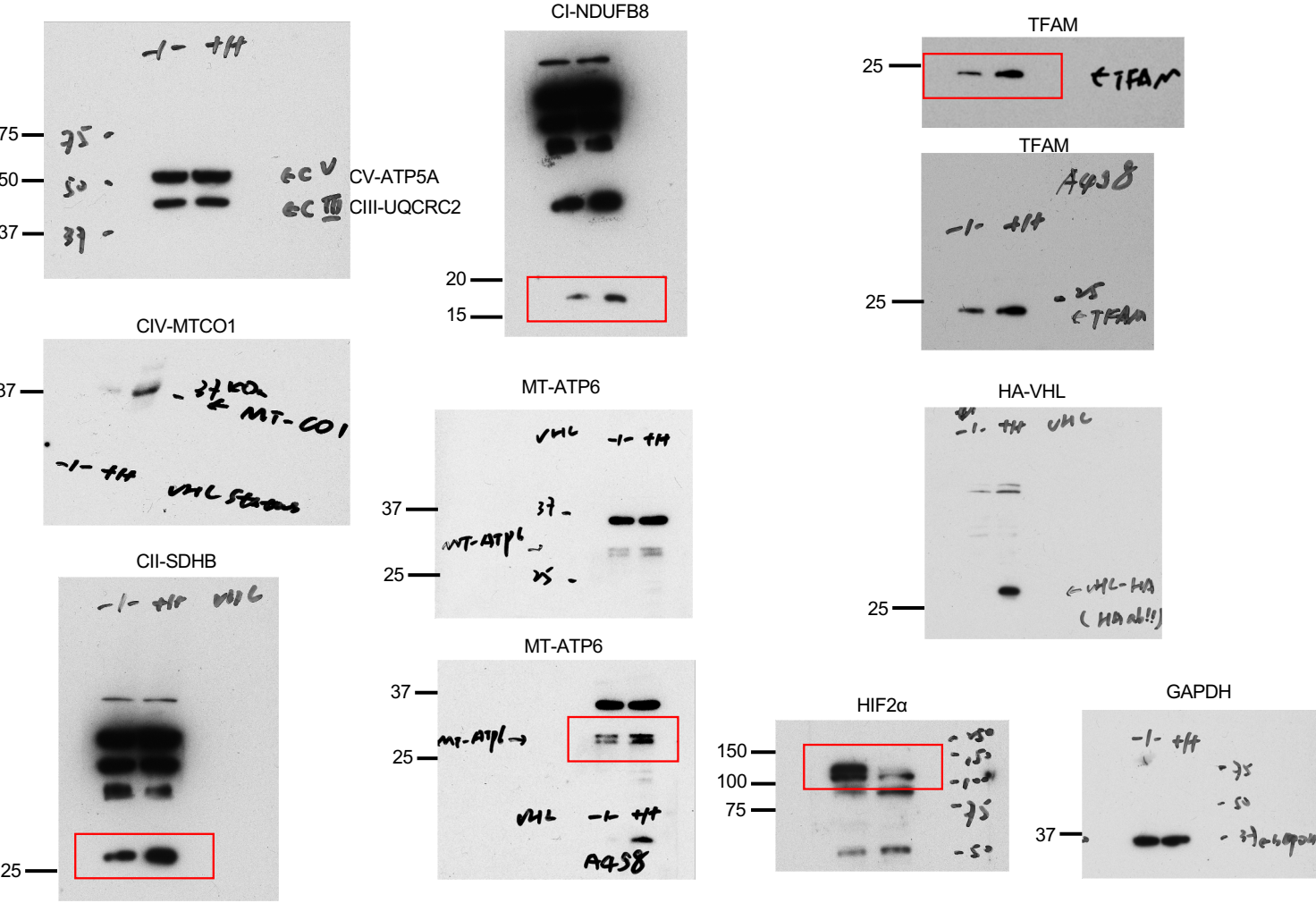

e

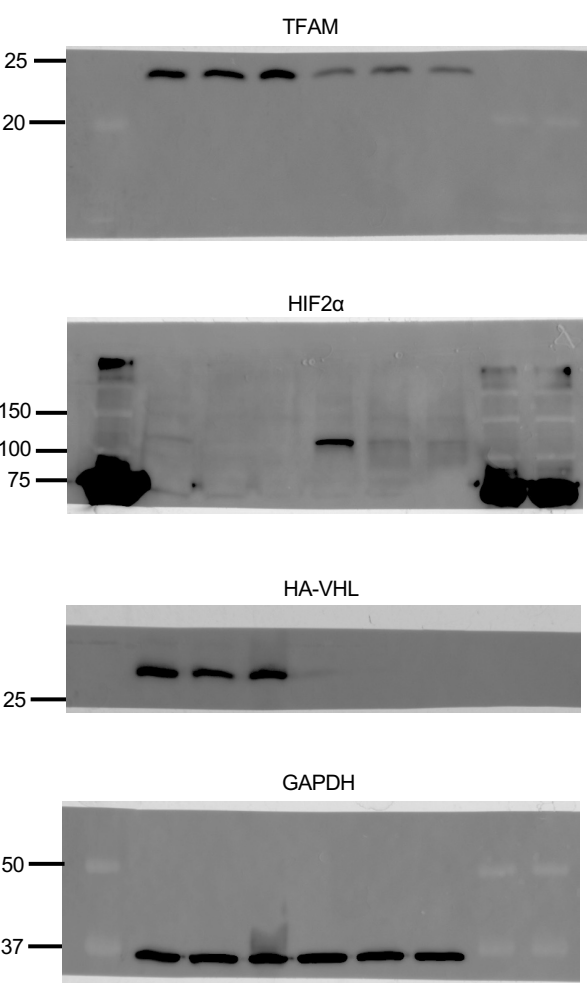

Supplement: Unprocessed_western_blots_EDFig1.pdf [file EMS145242-supplement-Unprocessed_western_blots_EDFig1_pdf.pdf]

Extended Data Fig. 2

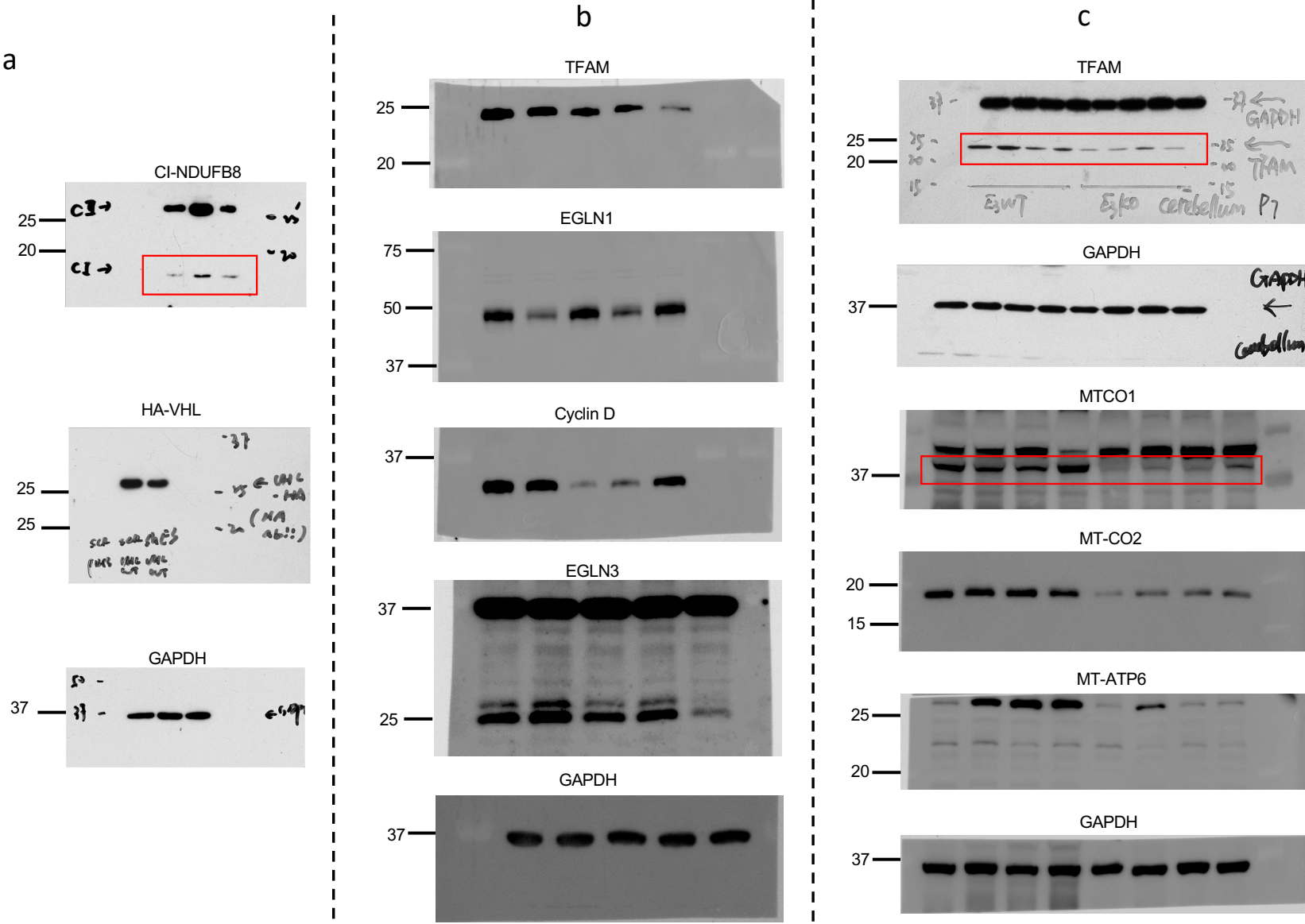

Extended Data Fig. 2

d

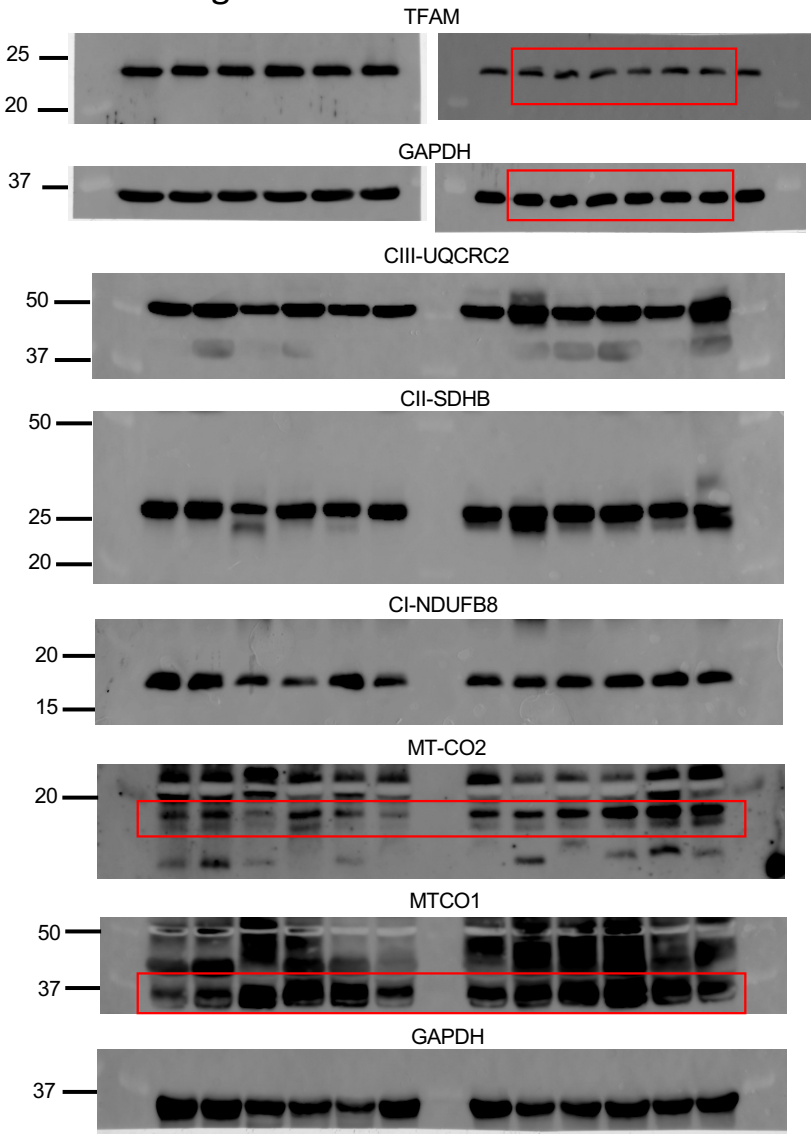

e

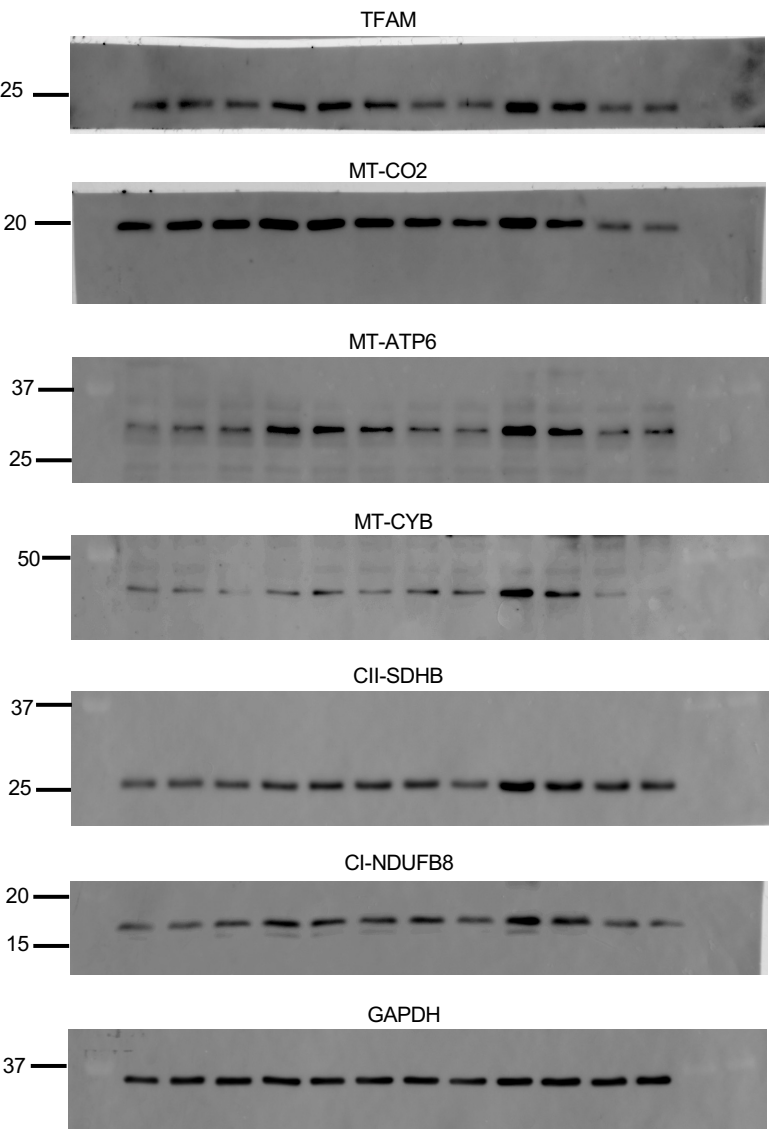

Extended Data Fig. 2

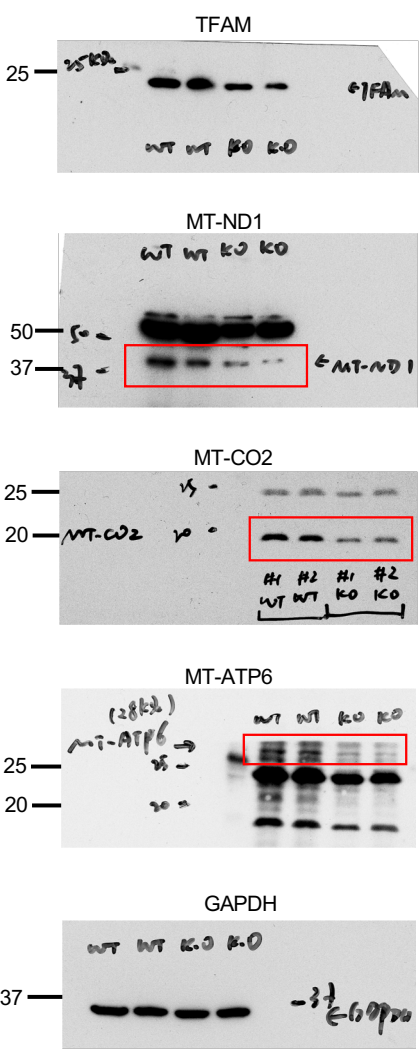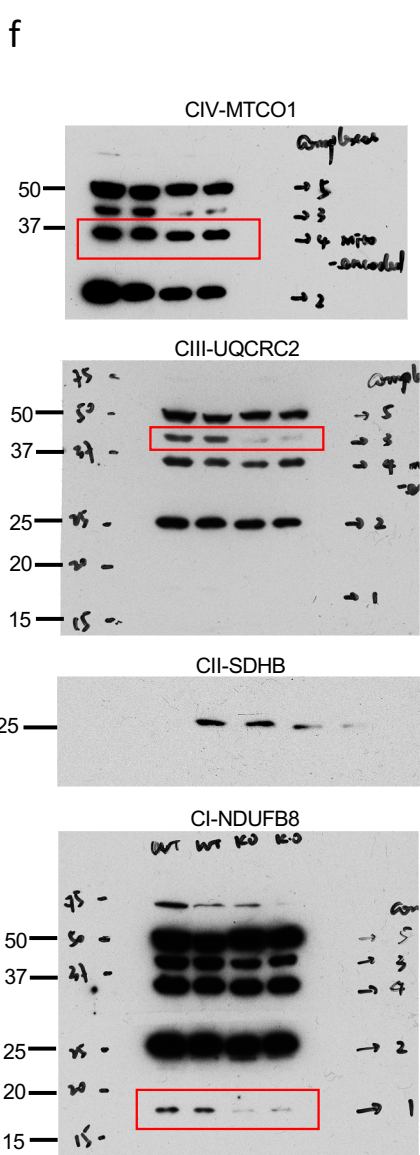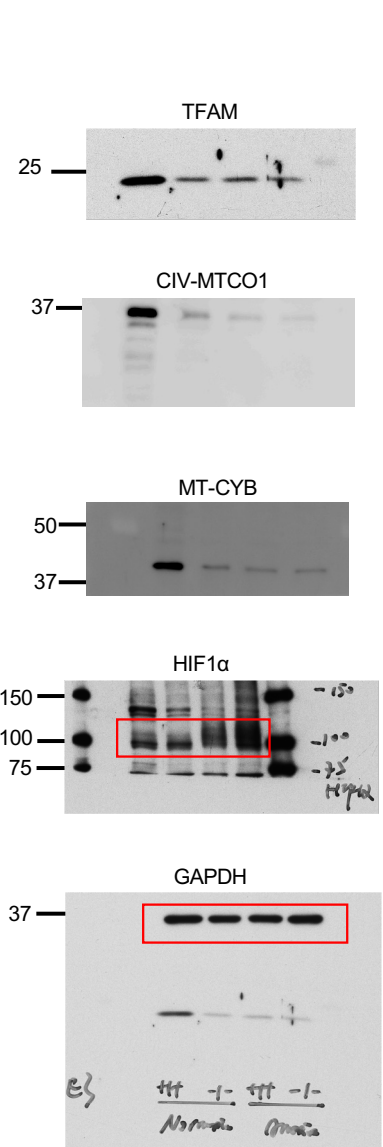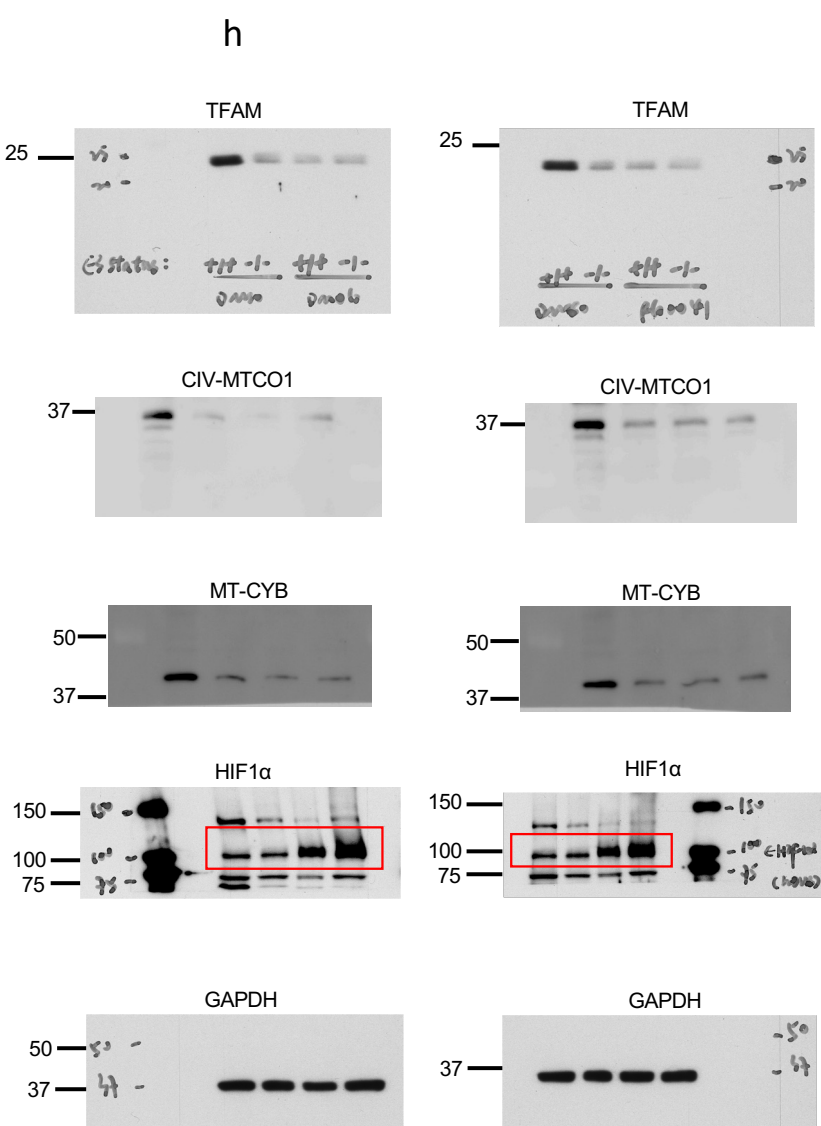

Supplement: Unprocessed_western_blots_EDFig2.pdf [file EMS145242-supplement-Unprocessed_western_blots_EDFig2_pdf.pdf]

Extended Data Fig. 3

a

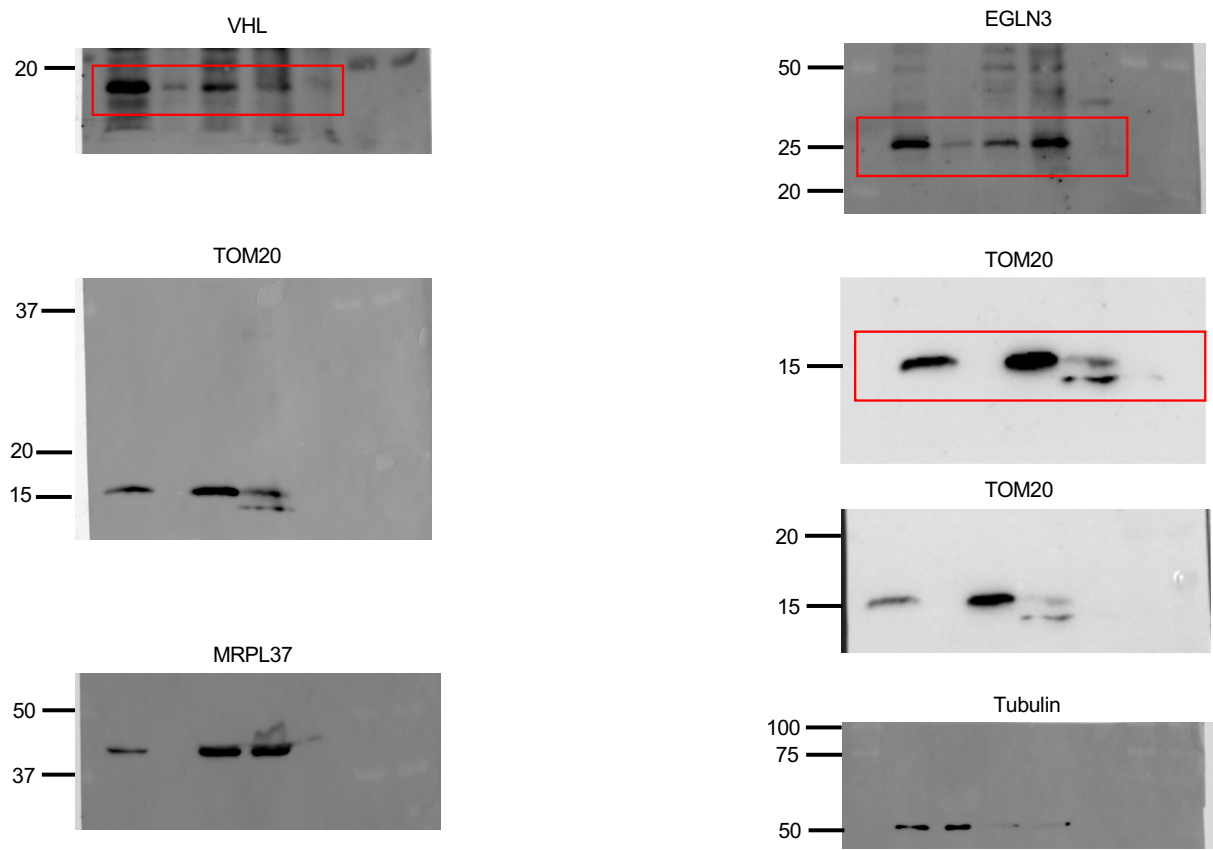

Supplement: Unprocessed_western_blots_EDFig3.pdf [file EMS145242-supplement-Unprocessed_western_blots_EDFig3_pdf.pdf]

Extended Data Fig. 4

a

Flag-TFAM

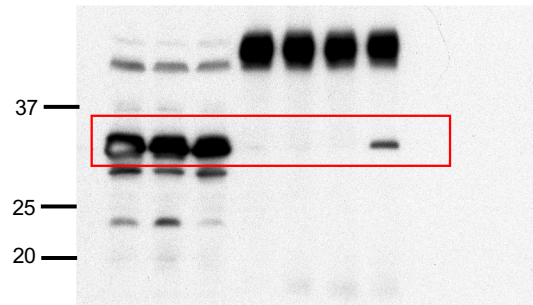

Flag-TFAM

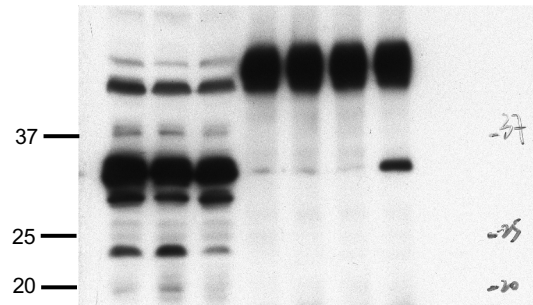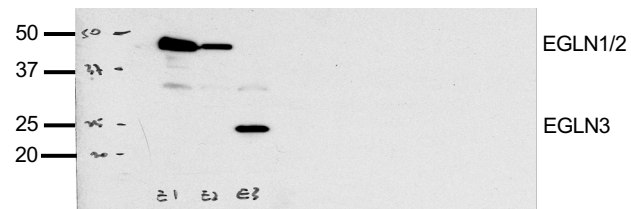

## h

S<sup>35</sup>VHL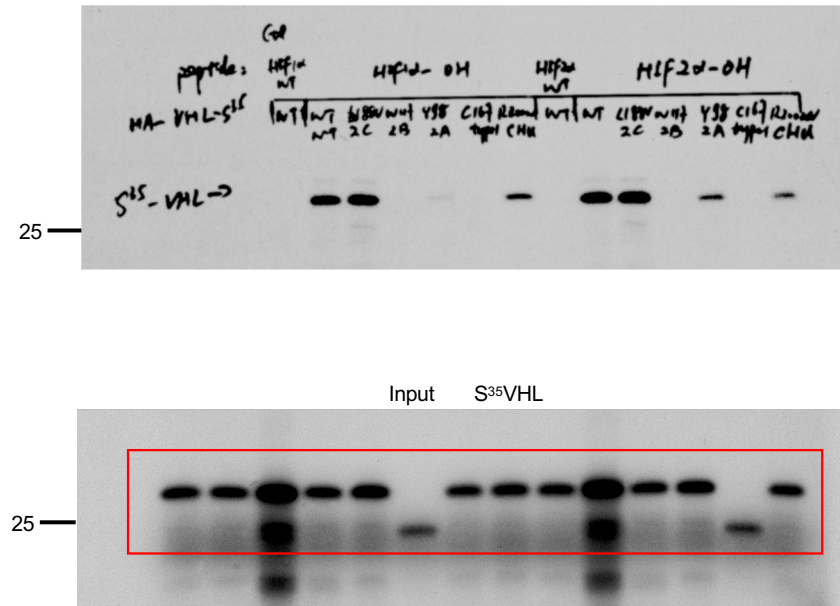

i

HA-VHL

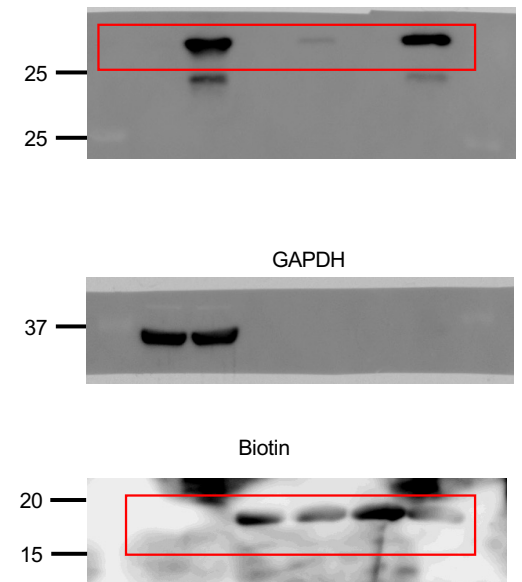

Supplement: Unprocessed_western_blots_EDFig4 [file EMS145242-supplement-Unprocessed_western_blots_EDFig4.pdf]

Extended Data Fig. 7

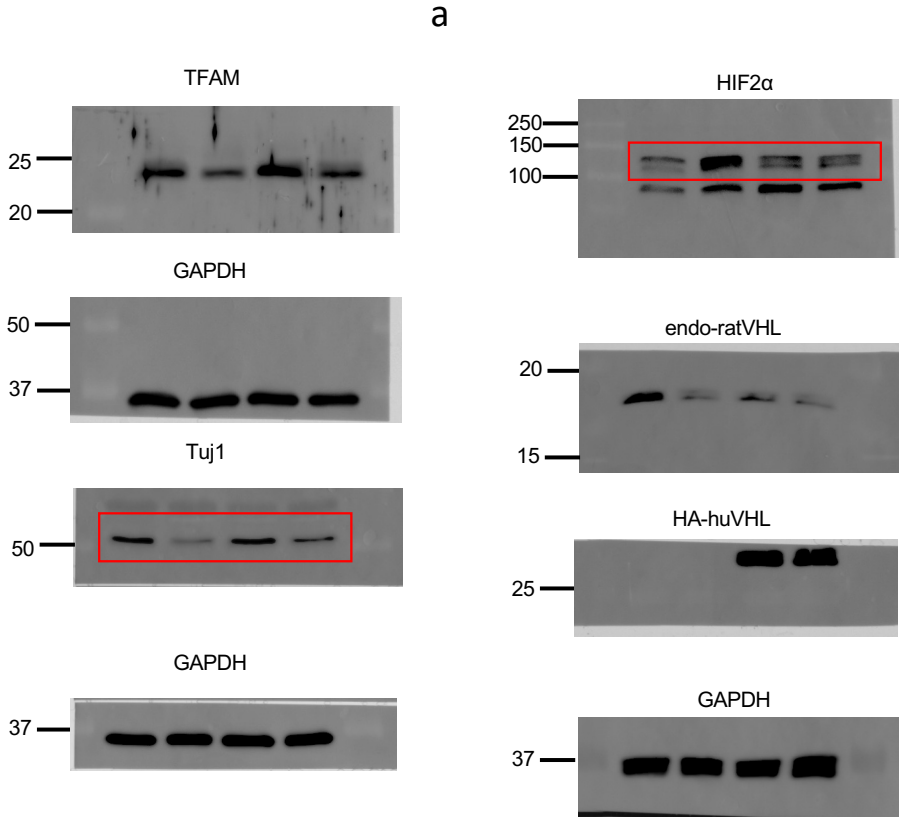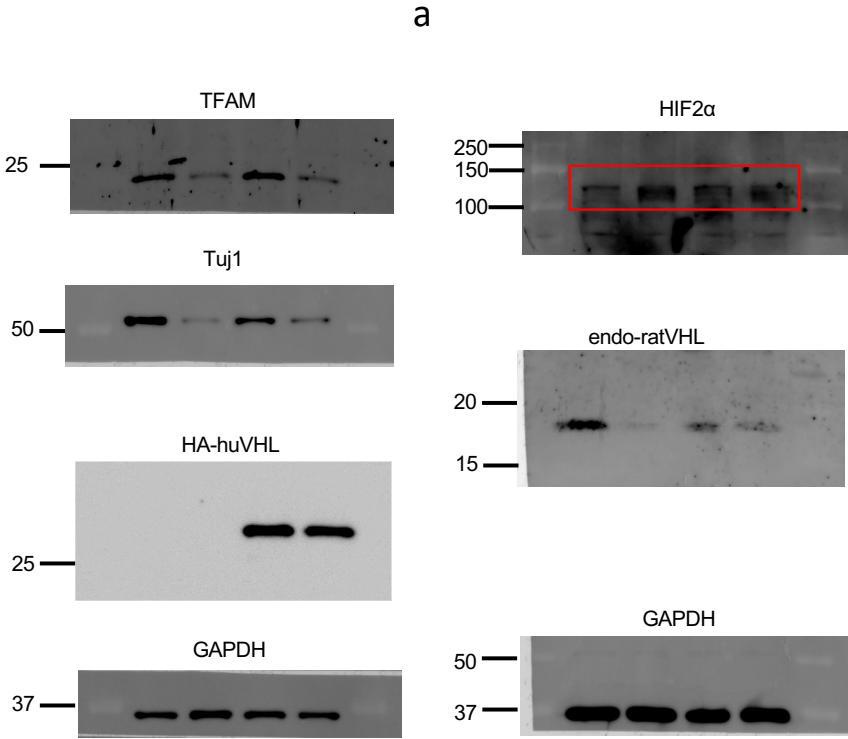

Supplement: Unprocessed_western_blots_EDFig7.pdf [file EMS145242-supplement-Unprocessed_western_blots_EDFig7_pdf.pdf]

Fig. 1

Fig. 1e

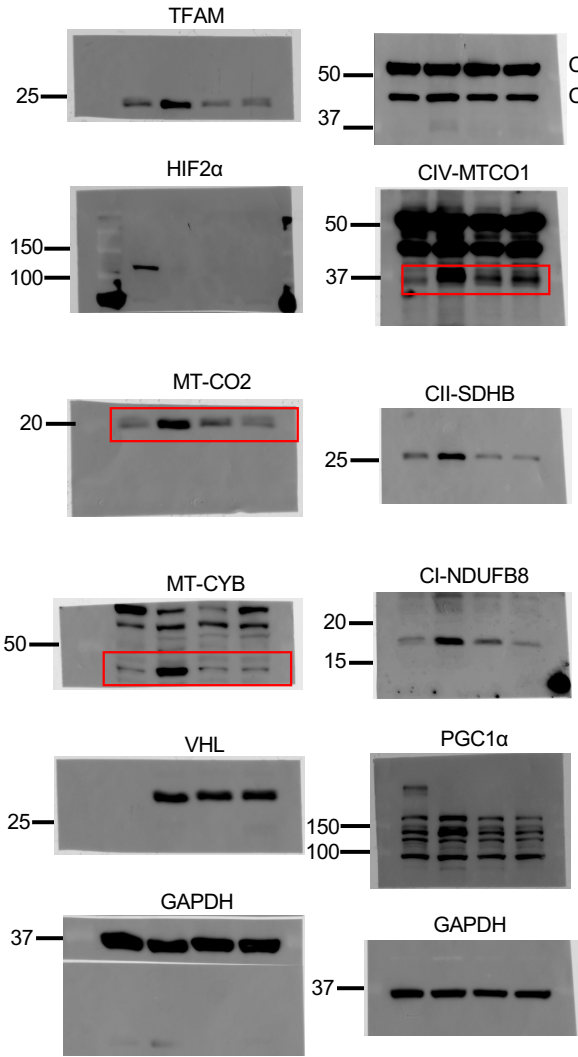

Fig. 1i

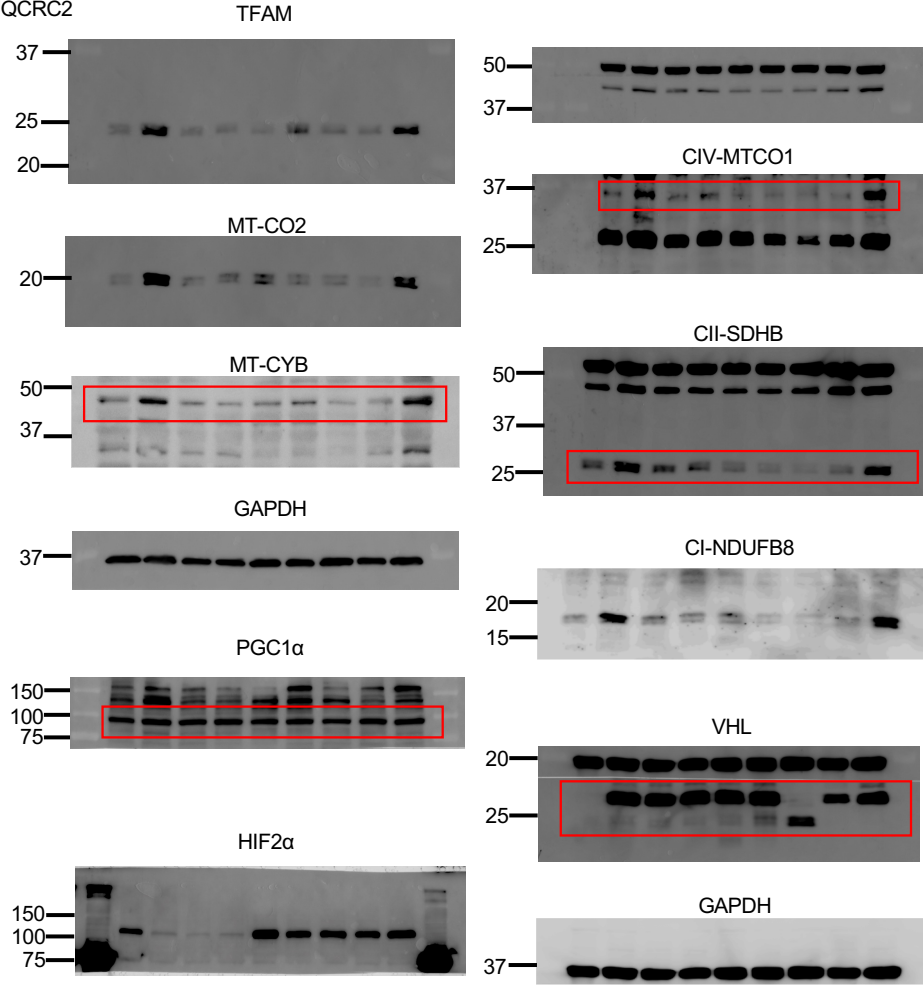

Fig. 1j

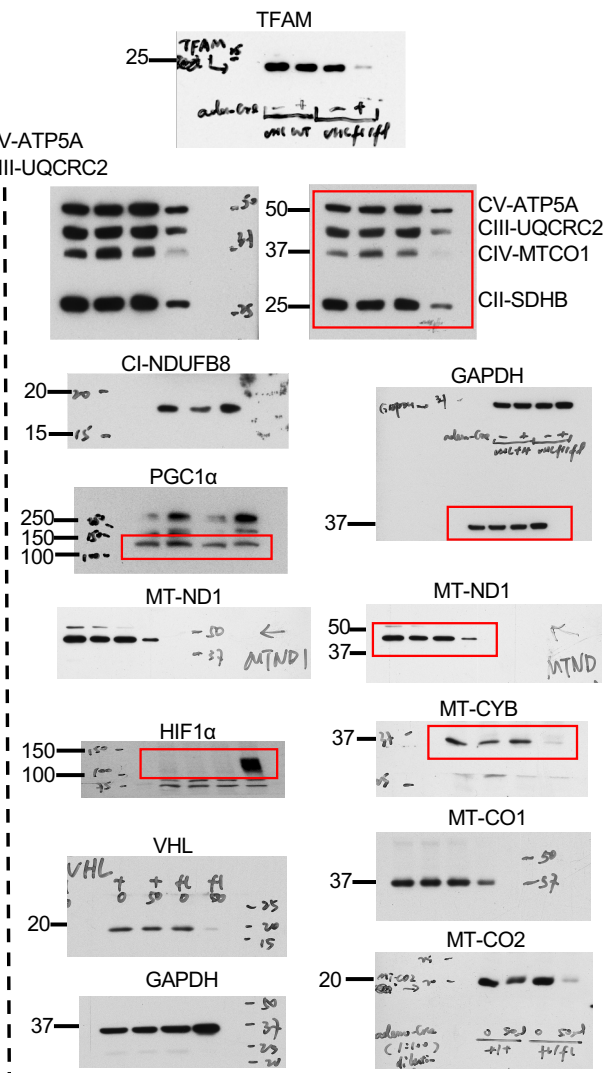

Supplement: Unprocessed_western_blots_Fig1.pdf [file EMS145242-supplement-Unprocessed_western_blots_Fig1_pdf.pdf]

Fig. 2

Fig. 2a

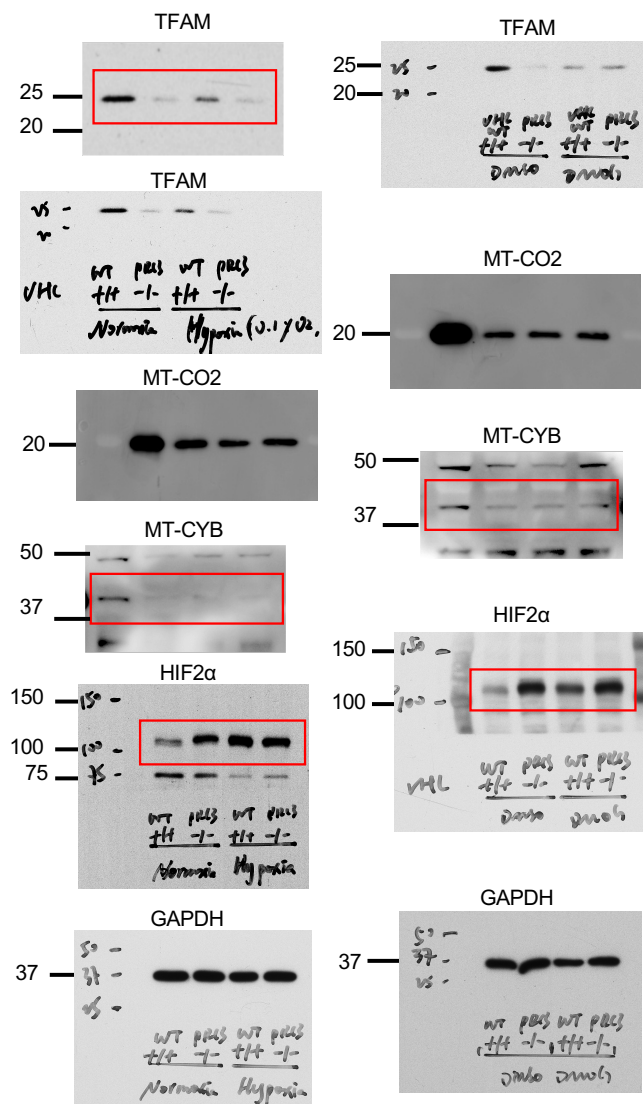

Fig. 2b

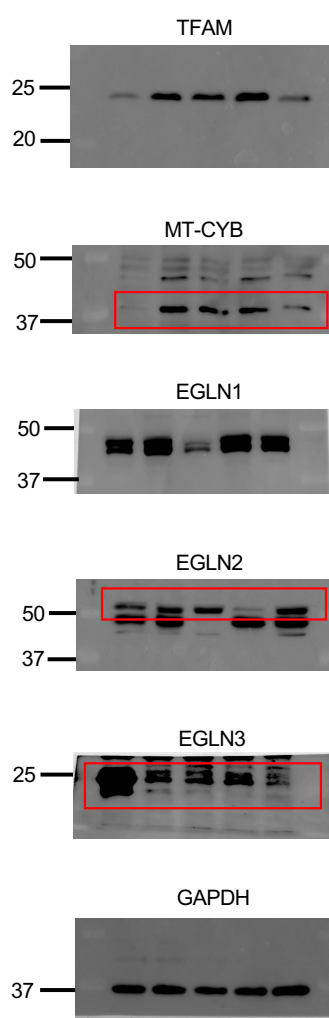

Fig. 2e

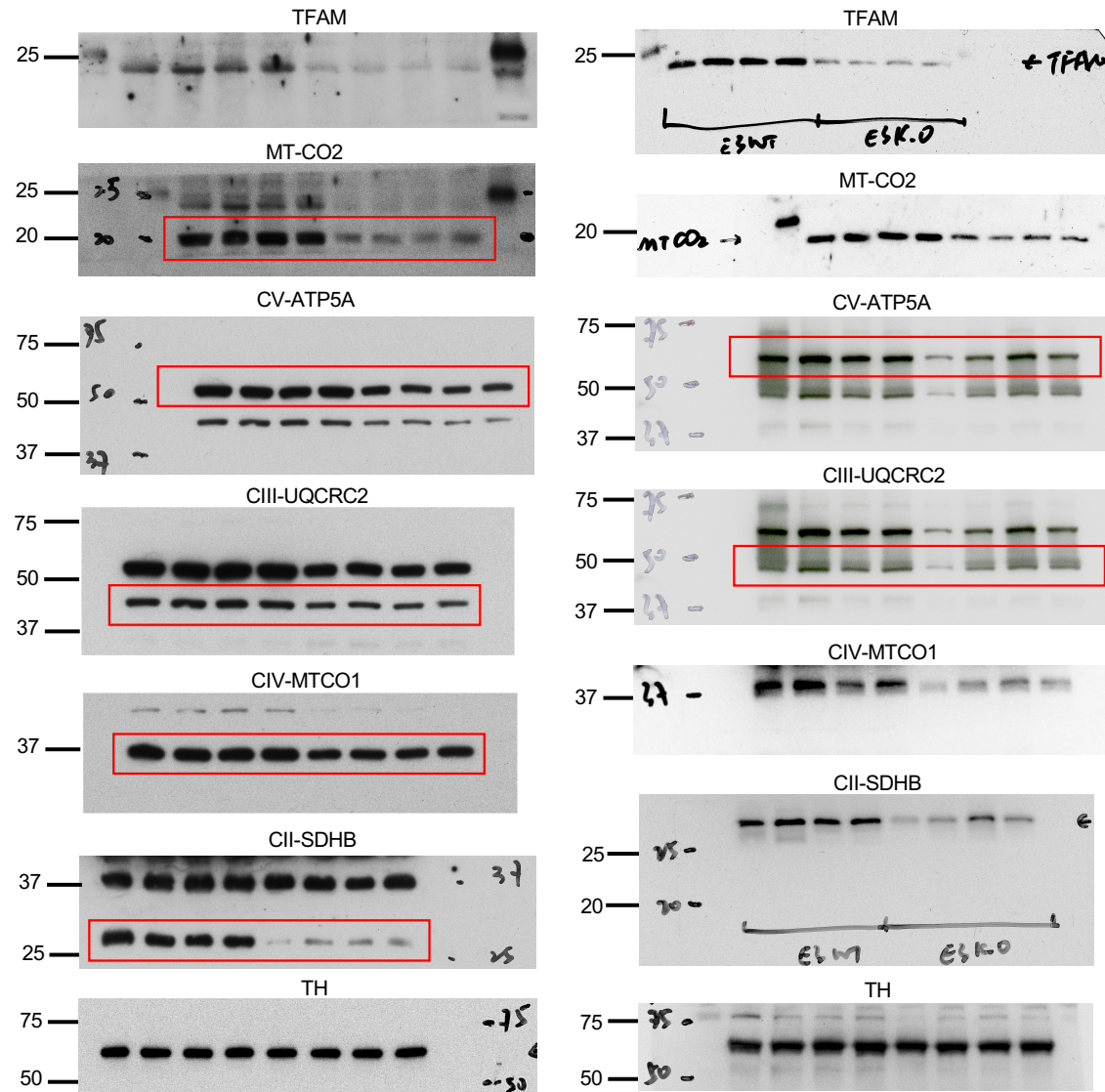

Fig. 2

Fig. 2f

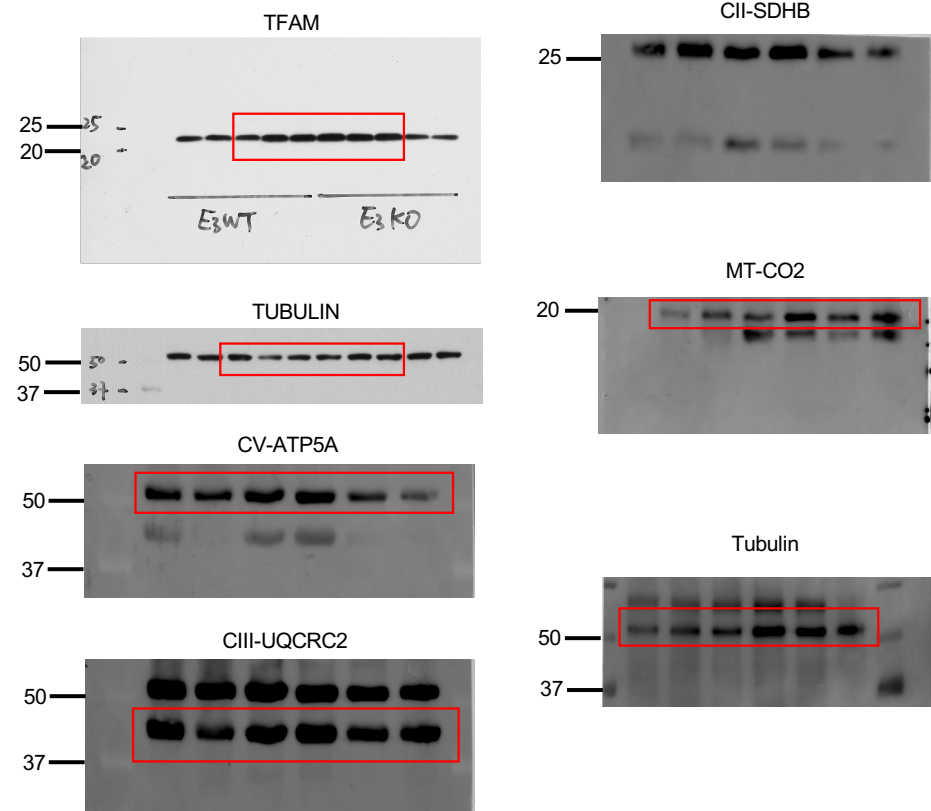

Fig. 2h

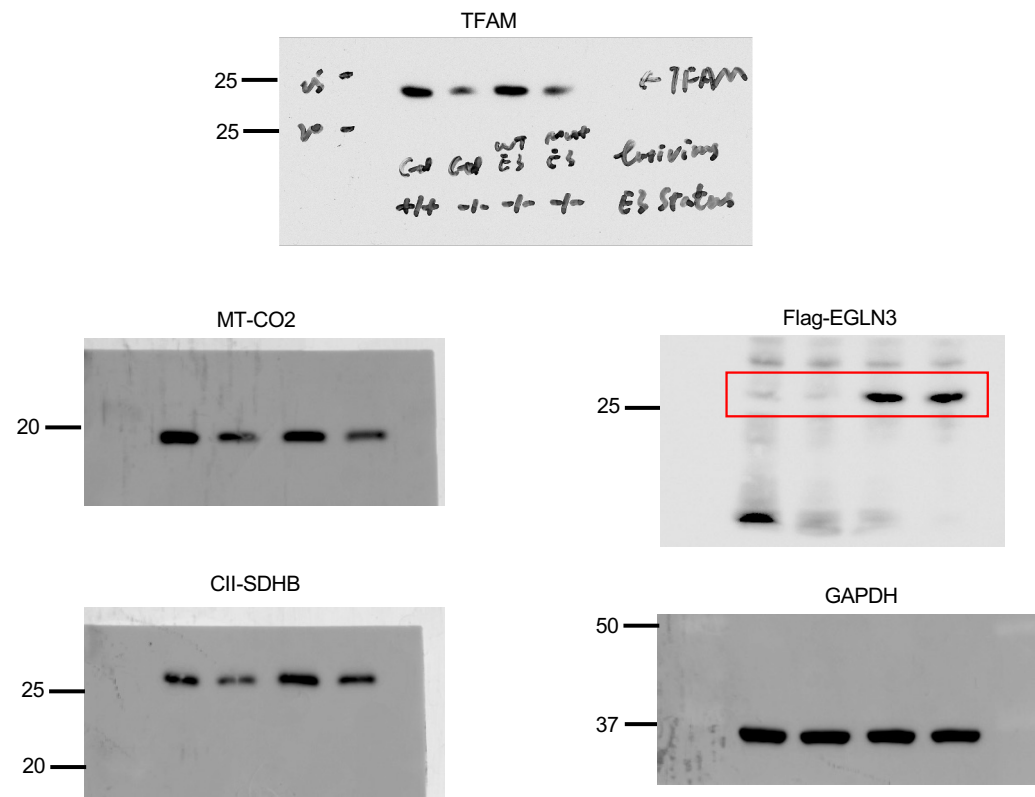

Supplement: Unprocessed_western_blots_Fig2.pdf [file EMS145242-supplement-Unprocessed_western_blots_Fig2_pdf.pdf]

Fig. 3

Fig. 3a

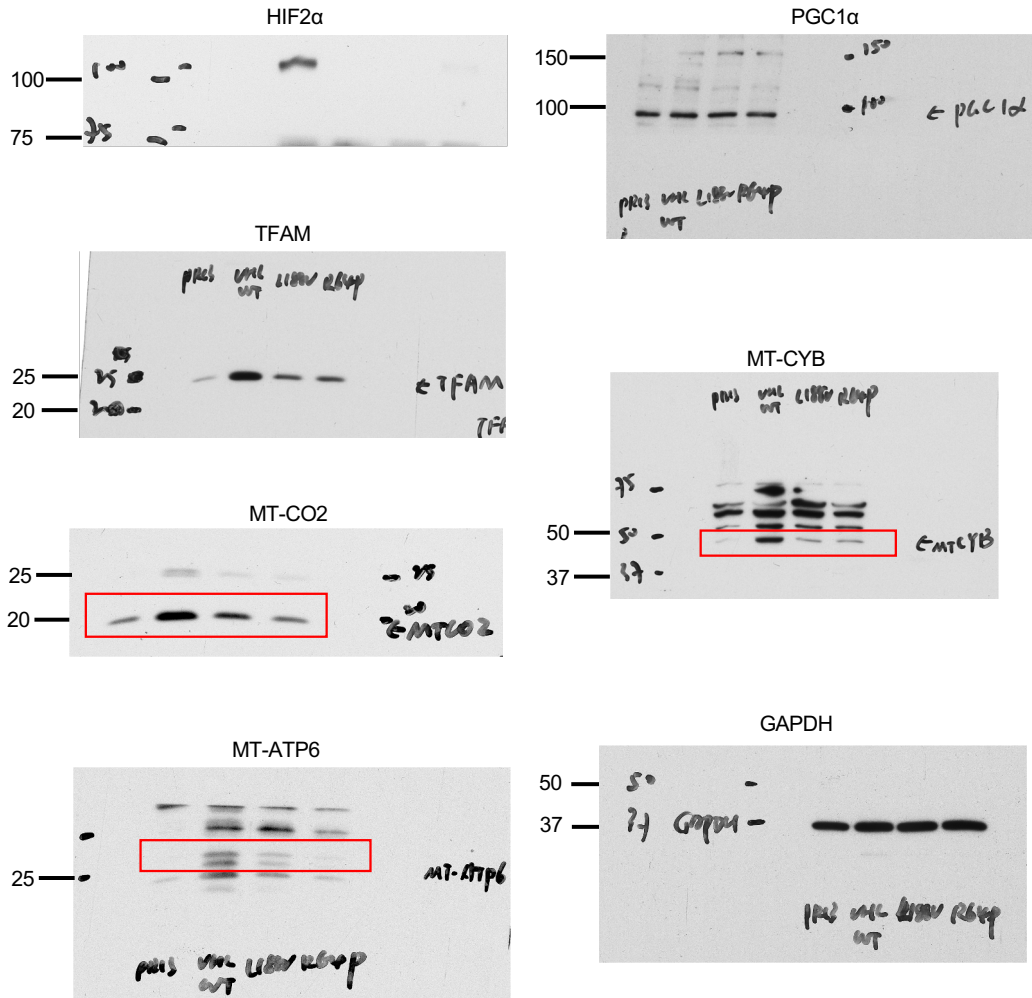

Fig. 3b

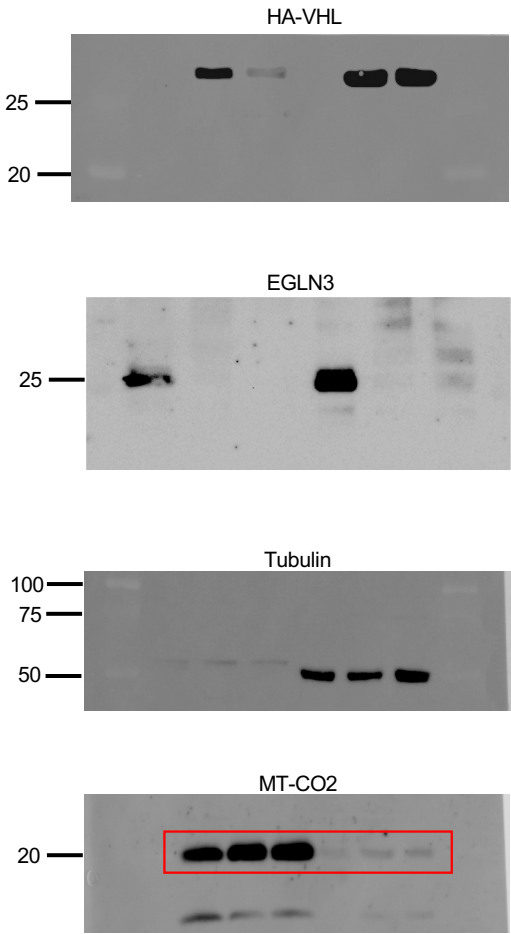

Fig. 3c

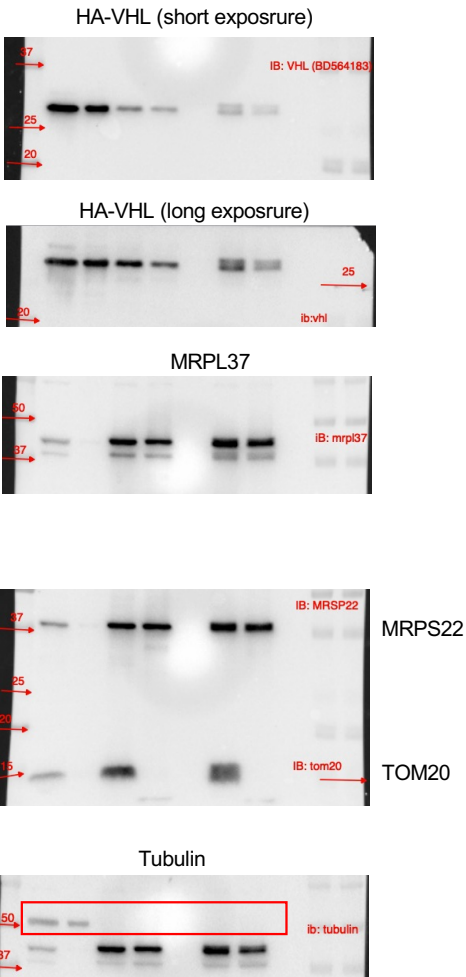

Fig. 3

Fig. 3f

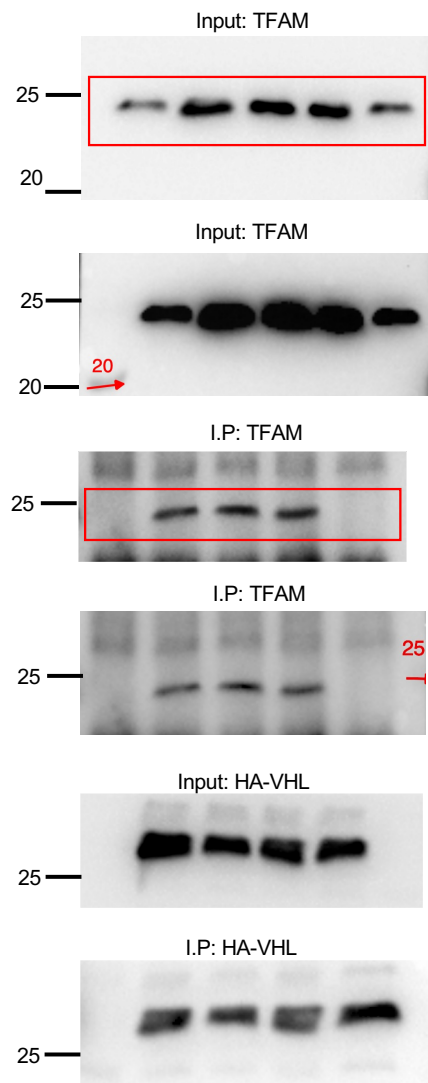

Fig. 3g

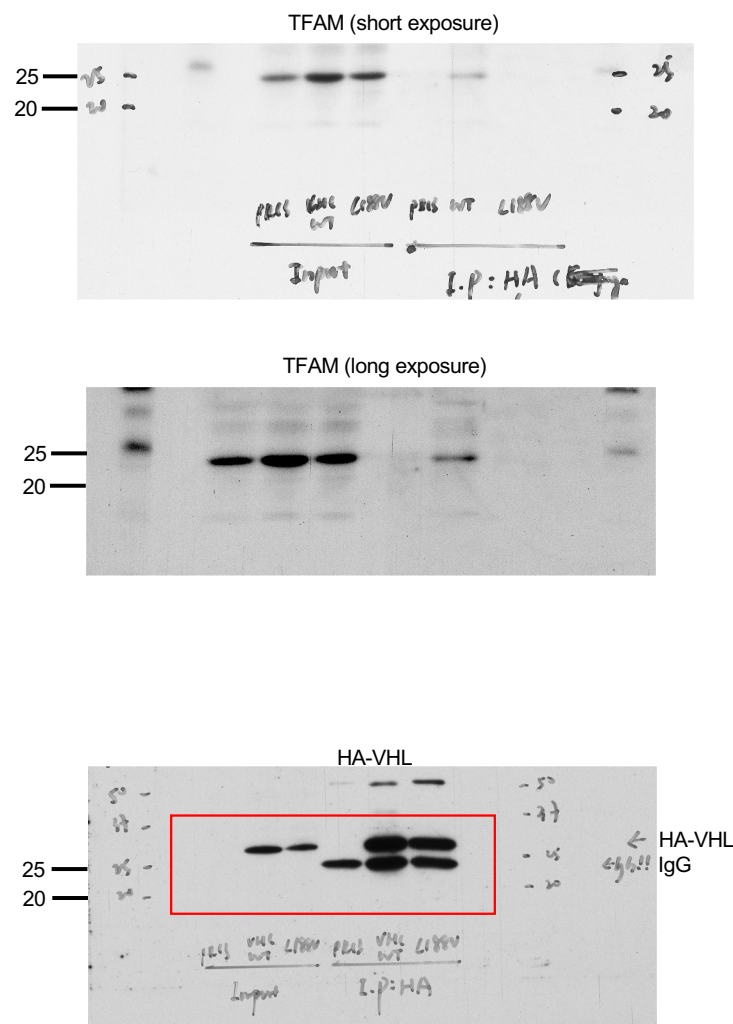

Fig. 3h

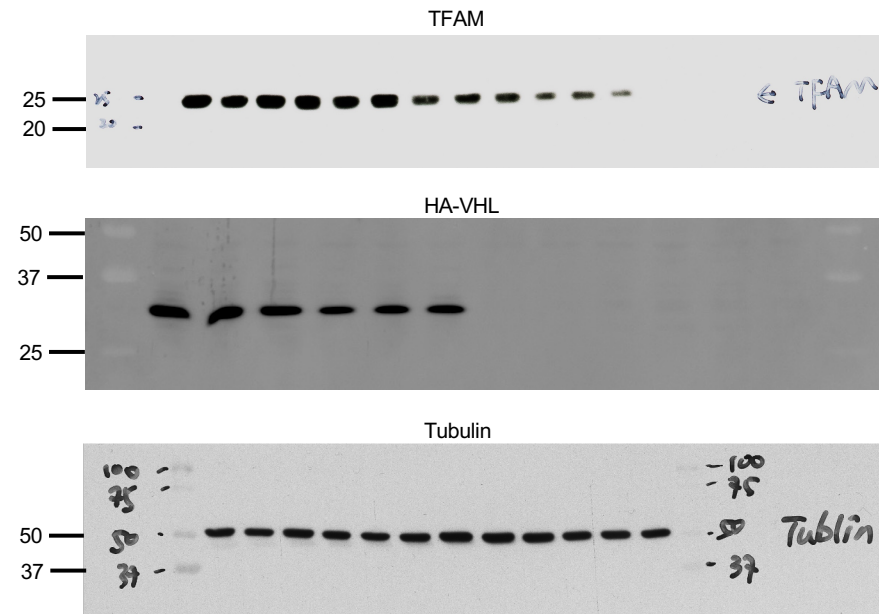

Fig. 3i

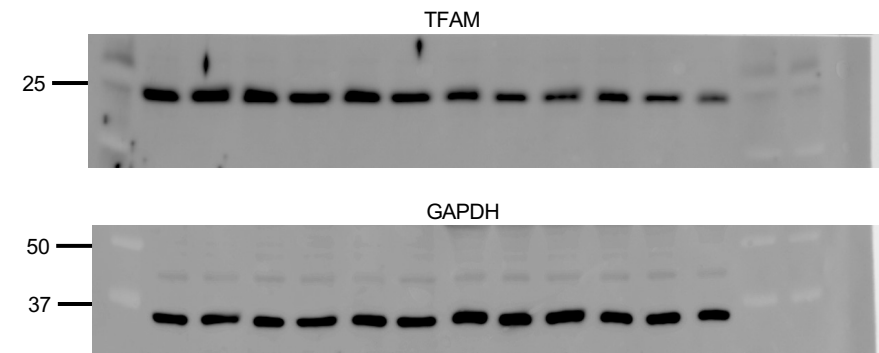

Supplement: Unprocessed_western_blots_Fig3.pdf [file EMS145242-supplement-Unprocessed_western_blots_Fig3_pdf.pdf]

Fig. 4

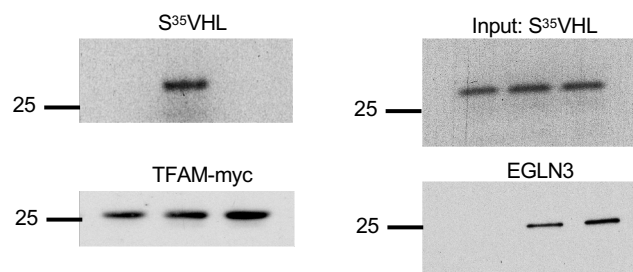

Fig. 4a

Fig. 4b

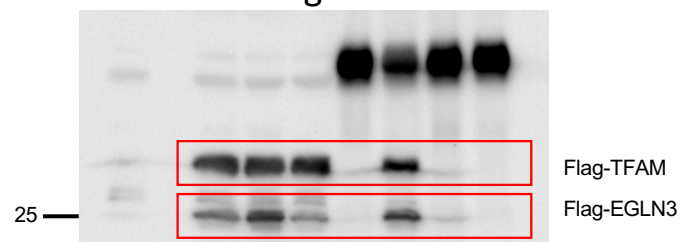

Fig. 4d

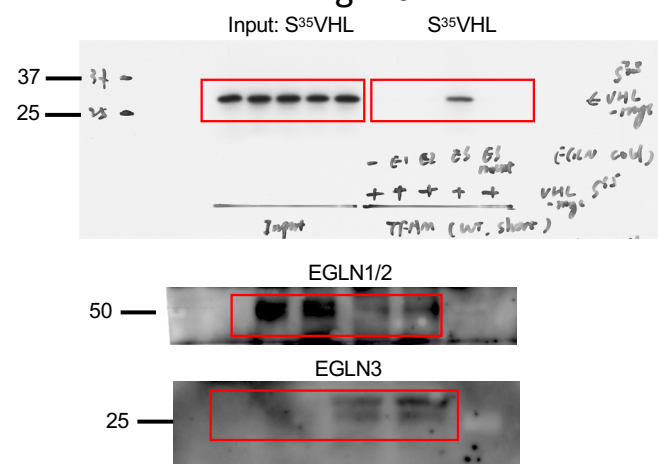

Fig. 4g

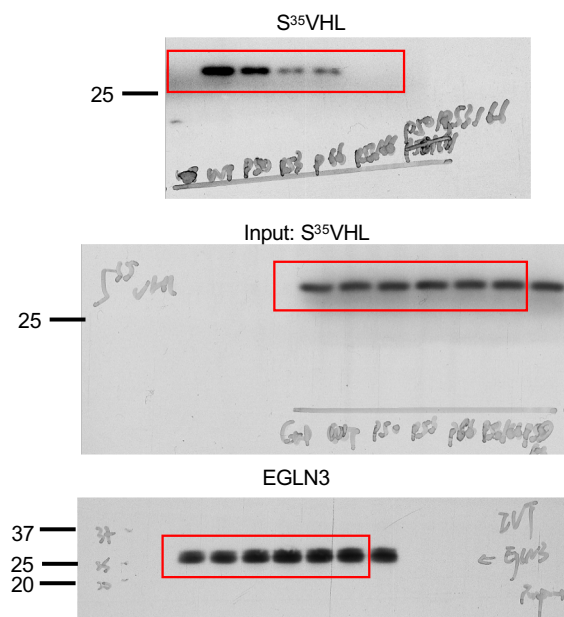

Fig. 4h

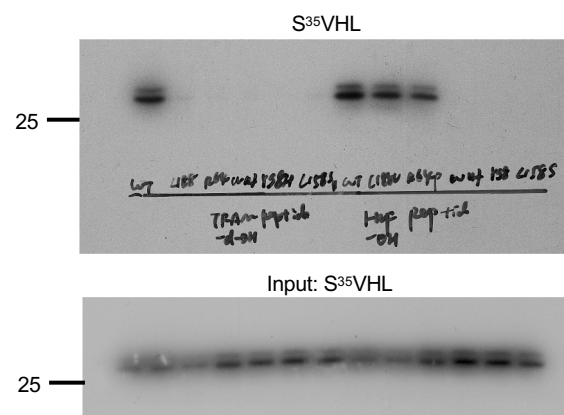

Fig. 4i

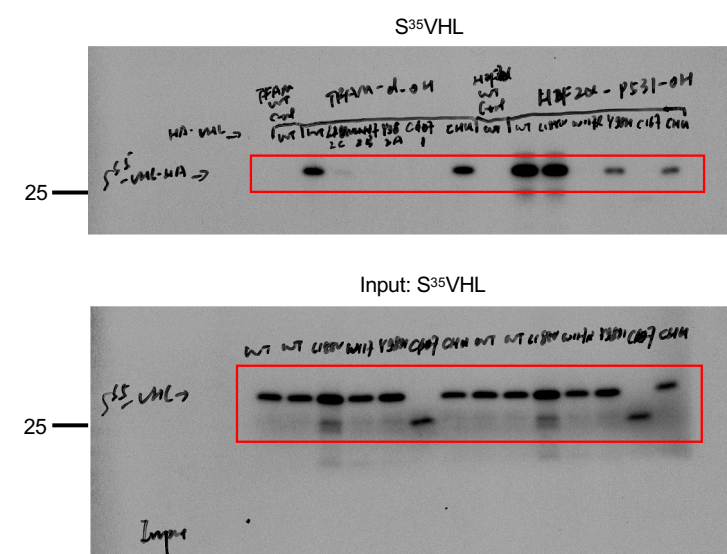

Fig. 4j

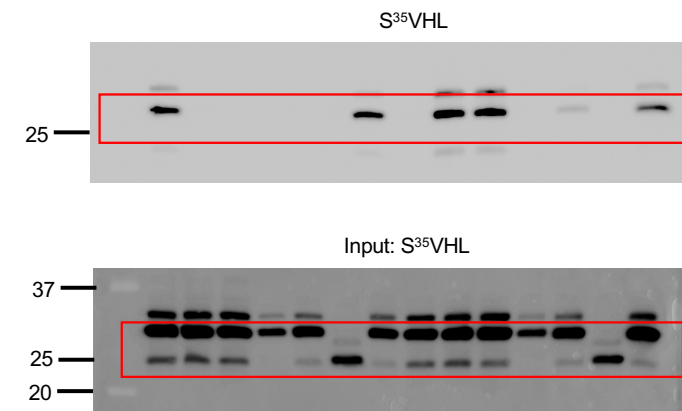

Supplement: Unprocessed_western_blots_Fig4.pdf [file EMS145242-supplement-Unprocessed_western_blots_Fig4_pdf.pdf]

Fig. 5

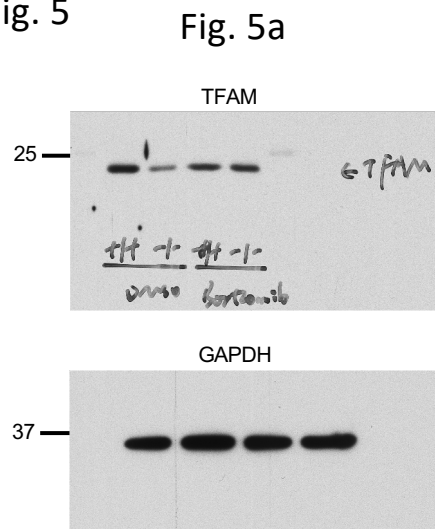

Fig. 5b

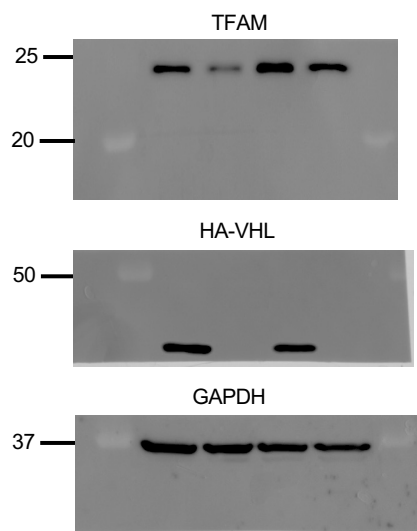

Fig. 5c

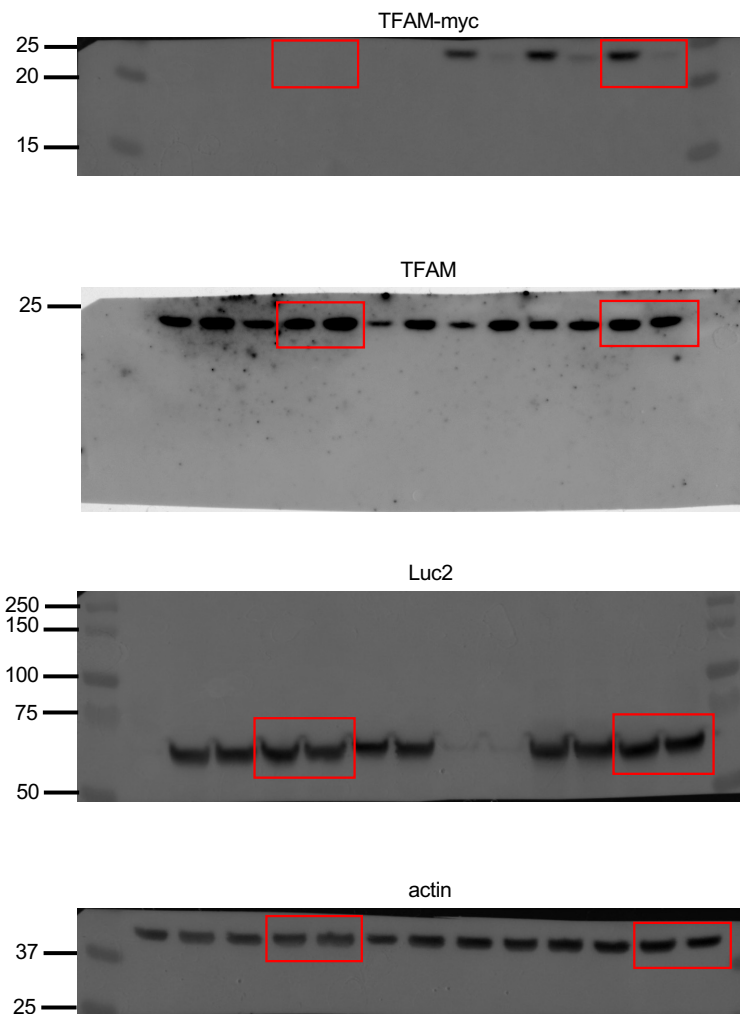

Fig. 5d

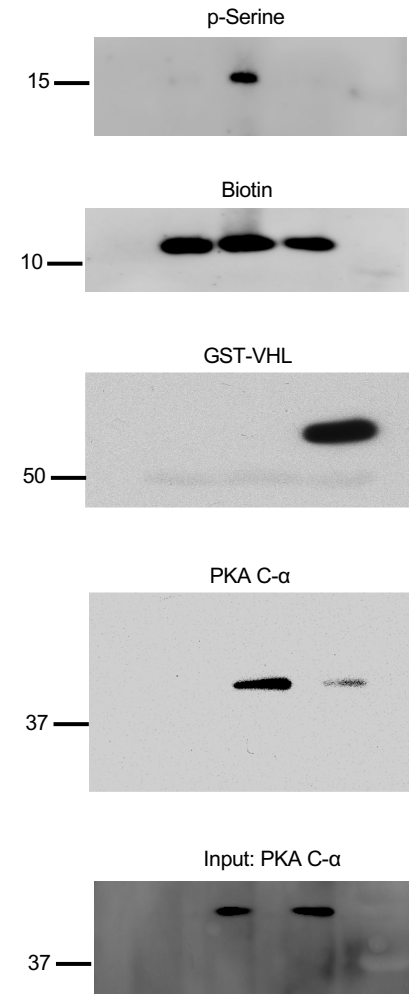

Fig. 5

Fig. 5e

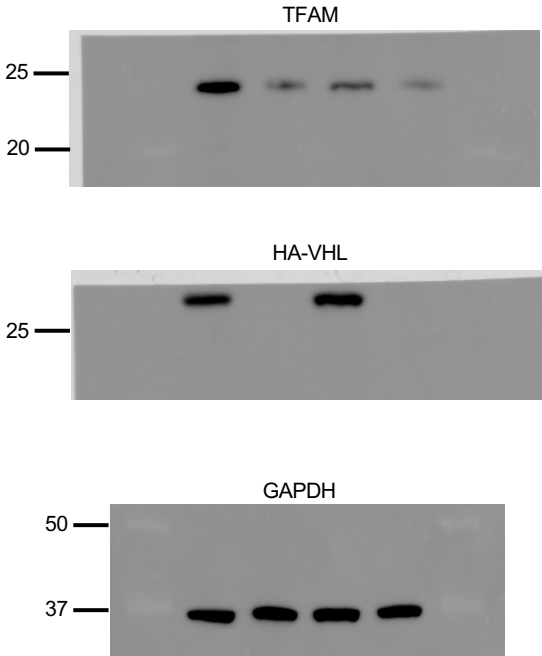

Fig. 5f

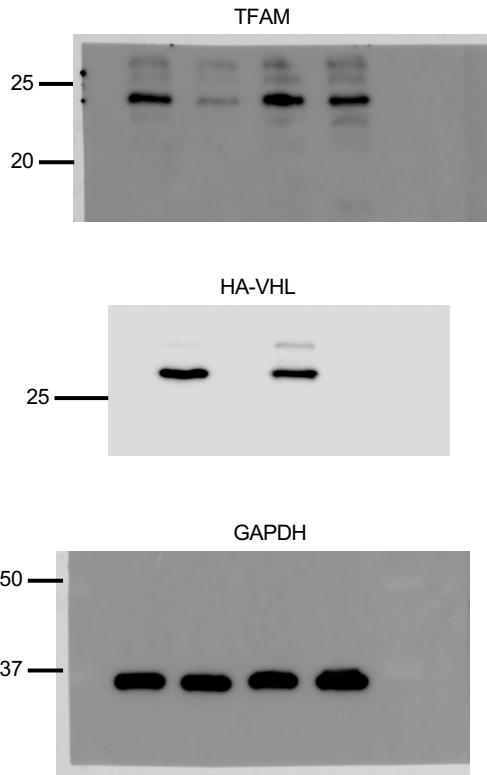

Fig. 5g

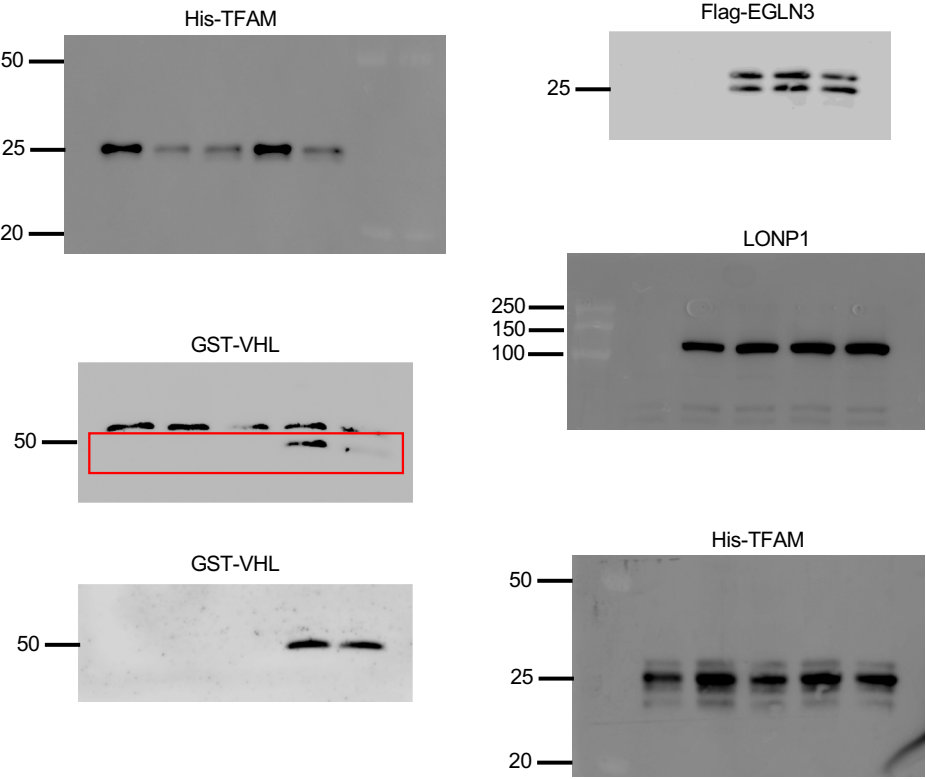

Supplement: Unprocessed_western_blots_Fig5.pdf [file EMS145242-supplement-Unprocessed_western_blots_Fig5_pdf.pdf]

Fig. 7

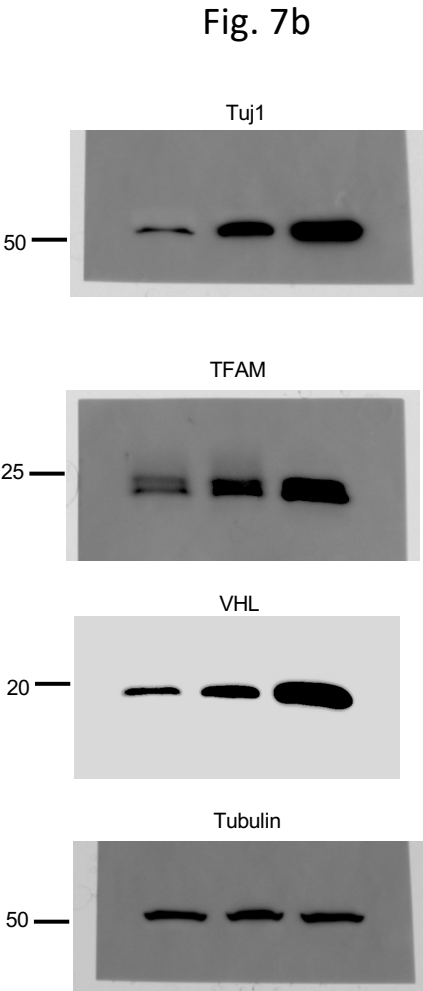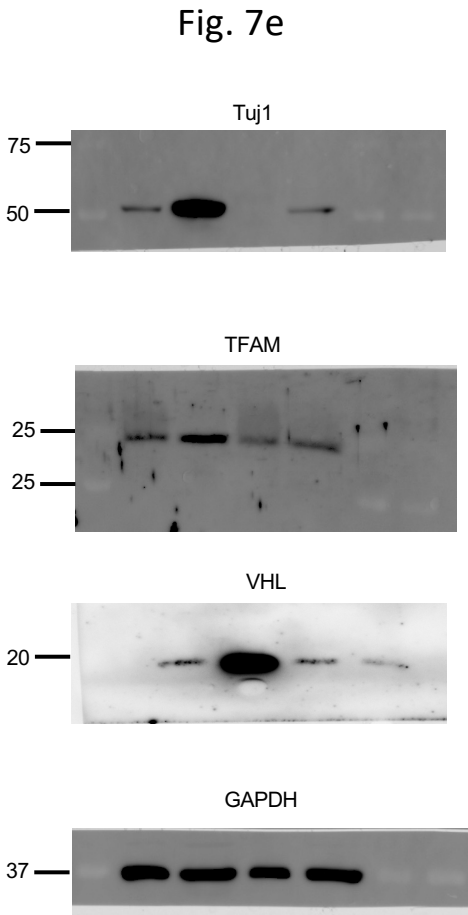

Supplement: Unprocessed_western_blots_Fig7.pdf [file EMS145242-supplement-Unprocessed_western_blots_Fig7_pdf.pdf]
