# Supplementary material for: Impaired oxygen-sensitive regulation of mitochondrial biogenesis within the von Hippel–Lindau syndrome
Source: Nat Metab. 2022 Jun 27;4(6):739–58. doi: 10.1038/s42255-022-00593-x (PMC9236906; doi:10.1038/s42255-022-00593-x)
Supplement: Supplementary file 1 [file 42255_2022_593_MOESM1_ESM.pdf]

nature portfolio

https://doi.org/10.1038/s42255-022-00593-x

Supplementary information

Impaired oxygen-sensitive regulation of mitochondrial biogenesis within the von Hippel–Lindau syndrome

In the format provided by the authors and unedited

Supplementary Information

Impaired oxygen-sensitive regulation of mitochondrial biogenesis within the von Hippel-Lindau syndrome.

Shuijie Li[1,2§], Wenyu Li[1*], Juan Yuan[1*], Petra Bullova[1], Jieyu Wu[1], Xuepei Zhang[3], Yong Liu[3], Monika Plescher[1], Javier Rodriguez[4], Oscar C. Bedoya-Reina[1], Paulo R. Jannig[5], Paula Valente-Silva[5], Meng Yu[6], Marie Arsenian Henriksson[1], Roman A. Zubarev[3], Anna Smed Sörensen[6], Carolyn K. Suzuki[7], Jorge L. Ruas[5], Johan Holmberg[8], Catharina Larsson[9], C. Christofer Juhlin[9], Alex von Kriegsheim[4] and Yihai Cao[1], Susanne Schlisio[1§]

[1]Department of Microbiology, Tumor and Cell Biology, Karolinska Institutet, Stockholm, Sweden

[2]College of Pharmacy, Harbin Medical University, Harbin, 150081 China

[3]Department of Medical Biochemistry and Biophysics, Stockholm, Sweden

[4]Edinburgh Cancer Research Centre, IGMM, University of Edinburgh, Edinburgh, UK

[5]Department of Physiology and Pharmacology, Karolinska Institutet, Stockholm, Sweden

[6]Department of Medicine, Karolinska University Hospital, Stockholm; Sweden

[7]Department of Biochemistry and Molecular Biology, New Jersey Medical School, University of Medicine and Dentistry of New Jersey, Newark, NJ 07103, USA

[8]Department of Molecular Biology, Faculty of Medicine, Umeå University, Umeå, Sweden.

[9]Department of Oncology-Pathology, Karolinska Institutet, Karolinska University Hospital, SE-17176 Stockholm, Sweden

* These authors contributed equally to this work

§ Corresponding authors: Shuijie Li (shuijie.li@ki.se), Susanne Schlisio (susanne.schlisio@ki.se)

Supplementary Figure 1

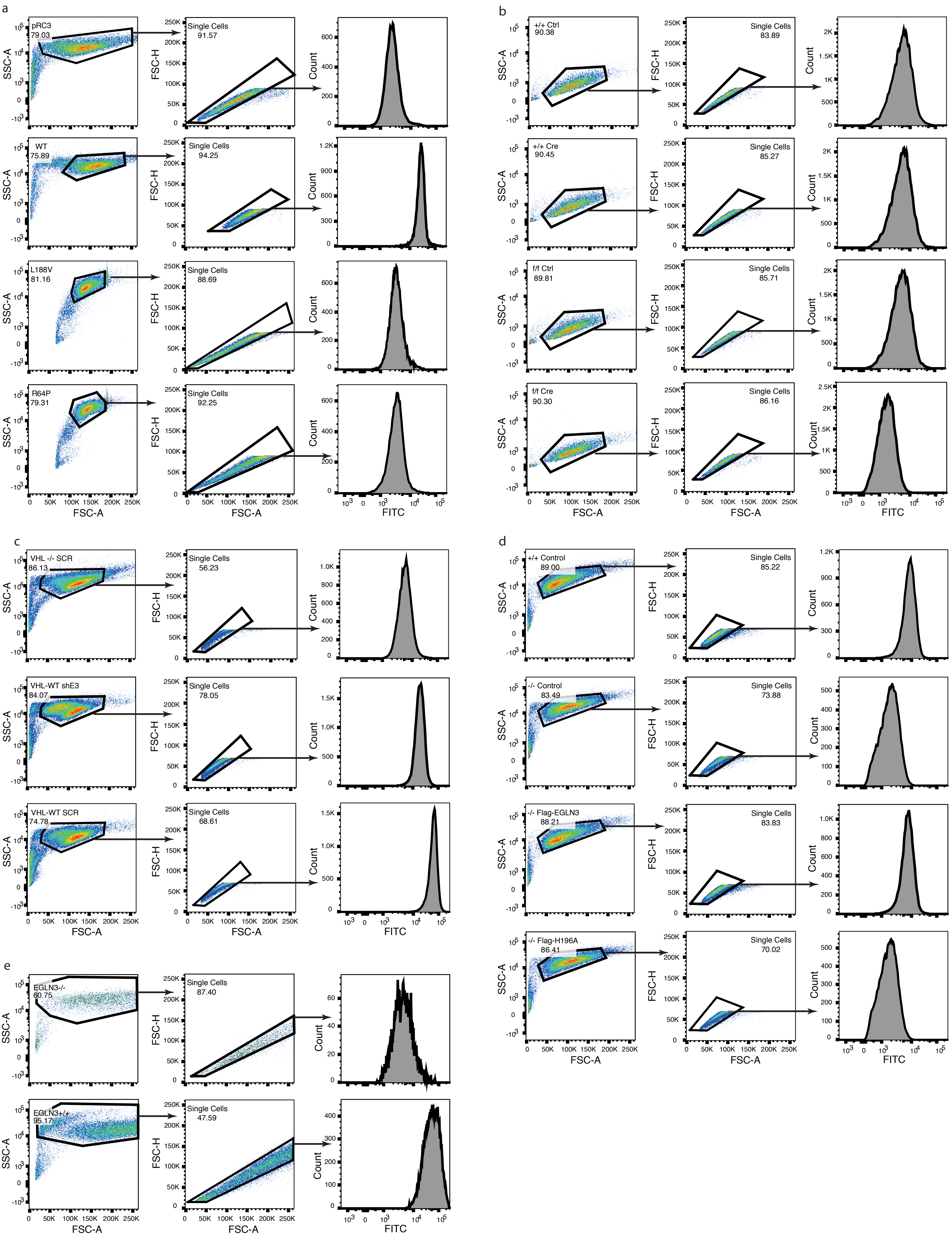

Supplementary Figure 1: FACS sequential gating strategies. Representative gating strategy of (a) Figure 1f, (b) Figure 1k, (c) Figure 2d, (d) Figure 2g and (e) Extended Data Fig. 2g. Briefly, fluorescence intensity was identified from single cells of each cell lines by flow cytometry.

Supplementary Figure 2

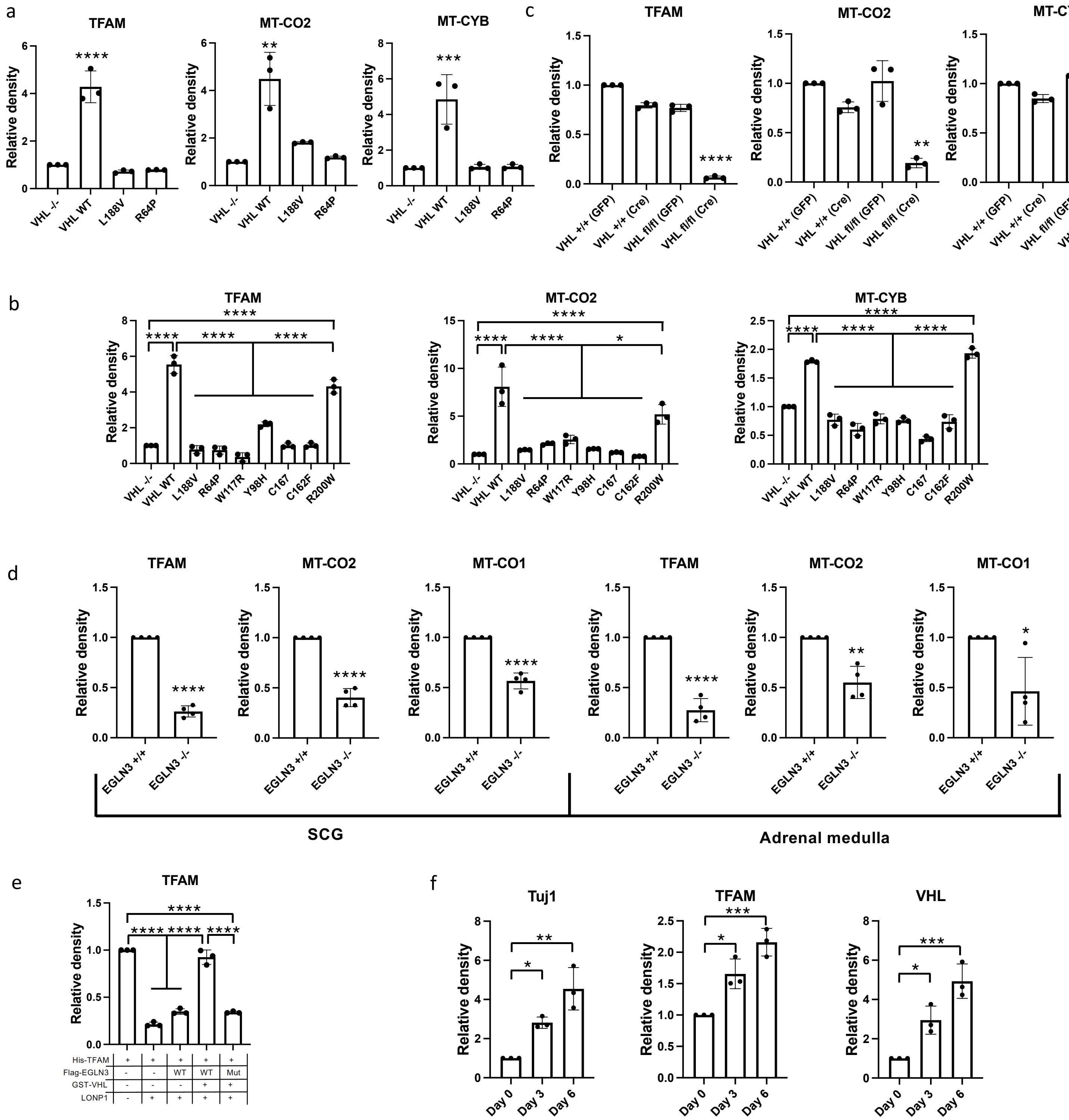

Supplementary Figure 2: Quantification of western blots.

a (Fig. 1e), b (Fig. 1i), c (Fig. 1j), e (Fig. 5g), f (Fig. 7b): Data are presented as mean values ± SD. Error bars show standard deviations (SD). One way ANOVA Tukey's Multiple Comparison Test. **** $p < 0.0001$, *** $p < 0.001$, ** $p < 0.01$, * $p < 0.05$. a (Fig. 1e): p =0.0017, p =0.0008. b (Fig. 1i): p = 0.0147. c (Fig. 1j): ** p =0.001. e (Fig. 5g): **** $p < 0.0001$.

f (Fig. 7b): p =0.0325, p=0.0013; p=0,012, p=0.0007; p=0.0253, p=0.0008.

d (Fig. 2e): Data are presented as mean values ± SD. Error bars show standard deviations (SD). Two-tailed unpaired *t* test. **** $p < 0.0001$, **p =0.0014, * p =0.0191.
