# Supplementary material for: Impaired oxygen-sensitive regulation of mitochondrial biogenesis within the von Hippel–Lindau syndrome
Source: Nat Metab. 2022 Jun 27;4(6):739–58. doi: 10.1038/s42255-022-00593-x (PMC9236906; doi:10.1038/s42255-022-00593-x)
Supplement: Supplementary file 2 [file 42255_2022_593_MOESM2_ESM.pdf]

nature research

Corresponding author(s): Shuijie Li & Susanne Schlisio

Last updated by author(s): May 12, 2022

Reporting Summary

Nature Research wishes to improve the reproducibility of the work that we publish. This form provides structure for consistency and transparency in reporting. For further information on Nature Research policies, see our Editorial Policies and the Editorial Policy Checklist.

Statistics

For all statistical analyses, confirm that the following items are present in the figure legend, table legend, main text, or Methods section.

n/a	Confirmed	
☐	☒	The exact sample size (n) for each experimental group/condition, given as a discrete number and unit of measurement
☐	☒	A statement on whether measurements were taken from distinct samples or whether the same sample was measured repeatedly
☐	☒	The statistical test(s) used AND whether they are one- or two-sided<br>*Only common tests should be described solely by name; describe more complex techniques in the Methods section.*
☒	☐	A description of all covariates tested
☐	☒	A description of any assumptions or corrections, such as tests of normality and adjustment for multiple comparisons
☐	☒	A full description of the statistical parameters including central tendency (e.g. means) or other basic estimates (e.g. regression coefficient) AND variation (e.g. standard deviation) or associated estimates of uncertainty (e.g. confidence intervals)
☐	☒	For null hypothesis testing, the test statistic (e.g. F, t, r) with confidence intervals, effect sizes, degrees of freedom and P value noted<br>*Give P values as exact values whenever suitable.*
☒	☐	For Bayesian analysis, information on the choice of priors and Markov chain Monte Carlo settings
☒	☐	For hierarchical and complex designs, identification of the appropriate level for tests and full reporting of outcomes
☒	☐	Estimates of effect sizes (e.g. Cohen's d, Pearson's r), indicating how they were calculated

Our web collection on statistics for biologists contains articles on many of the points above.

Software and code

Policy information about availability of computer code

Data collection	ATP/ADP ratio data were collected using Microsoft Excel.<br>The oxygen consumption rate (OCR) data were collected using Microsoft Excel and GraphPad Prism (Version 8.3.0 and Version 9).<br>Proximity Ligation Assay (PLA) statistical analysis data were collected using GraphPad Prism 9.<br>Flow cytometry data were collected FACSDiva 8.0 (BD Biosciences).<br>The nanoLC-MS/MS raw data files were collected using Xcalibur (Thermo Fisher Scientific) and directly loaded in Proteome Discoverer v2.3 and searched against human SwissProt protein databases (21,008 entries) using the Mascot 2.5.1 search engine (Matrix Science Ltd.).
Data analysis	All graphing and numeric data were analyzed with Microsoft Excel and GraphPad Prism.<br>STRING v10.5, DAVID and REVIGO were used for LC-MS/MS analysis.<br>Flow cytometry data were analysed using FlowJo software (Tree Star).<br>Western blot quantification was performed using Image J.

For manuscripts utilizing custom algorithms or software that are central to the research but not yet described in published literature, software must be made available to editors and reviewers. We strongly encourage code deposition in a community repository (e.g. GitHub). See the Nature Research guidelines for submitting code & software for further information.

Data

Policy information about availability of data

All manuscripts must include a data availability statement. This statement should provide the following information, where applicable:

- Accession codes, unique identifiers, or web links for publicly available datasets
- A list of figures that have associated raw data
- A description of any restrictions on data availability

Raw and analyzed mass spectrpmetry data is available at http://www.ebi.ac.uk/pride Username: reviewer_pxd022734@ebi.ac.uk Password: 3Up3pAom
All data are available on request from corresponding authors.

Field-specific reporting

Please select the one below that is the best fit for your research. If you are not sure, read the appropriate sections before making your selection.

☒ Life sciences ☐ Behavioural & social sciences ☐ Ecological, evolutionary & environmental sciences

For a reference copy of the document with all sections, see nature.com/documents/nr-reporting-summary-flat.pdf

Life sciences study design

All studies must disclose on these points even when the disclosure is negative.

Sample size	No statistical method to determine the sample sizes in this study. Sample sizes were chosen based on standard protocol and reference to existing literature (PMID: 35315437; PMID: 35228743; PMID: 31375625; PMID: 30067985)
Data exclusions	No data were excluded.
Replication	All attempts at replication were successful and the times of replication were stated in the figure legends.
Randomization	Human pheochromocytoma samples and renal cell carcinoma cells were randomized prior to nanoLC-MS/MS analysis. The samples or cells were randomized to be examined. The mice were randomly allocated to the experimental groups.
Blinding	Animal experiments were performed blinded to the genotype the mice. During the data collection and/or analysis, the xenograft experiment was not blinded since the nude mice with different treatment should be identifiable. The human pheochromocytoma samples and renal cell carcinoma cells were subjected to nanoLC-MS/MS analysis in a manner blinded to sample identity. During the data collection and/or analysis, all the samples were re-identified since samples groups with different genotypes should be identifiable.

Reporting for specific materials, systems and methods

We require information from authors about some types of materials, experimental systems and methods used in many studies. Here, indicate whether each material, system or method listed is relevant to your study. If you are not sure if a list item applies to your research, read the appropriate section before selecting a response.

Materials & experimental systems

n/a	Involved in the study	
☐	☒	Antibodies
☐	☒	Eukaryotic cell lines
☒	☐	Palaeontology and archaeology
☐	☒	Animals and other organisms
☐	☒	Human research participants
☒	☐	Clinical data
☒	☐	Dual use research of concern

Methods

n/a	Involved in the study	
☒	☐	ChIP-seq
☐	☒	Flow cytometry
☒	☐	MRI-based neuroimaging

Antibodies

Antibodies used	Rabbit monoclonal anti-TFAM (Cell Signaling Technology, Cat# 8076), Rabbit polyclonal anti-PKA C-α (Cell Signaling Technology, Cat# 4782), Rabbit monoclonal anti-PHD-2/Egln1 (Cell Signaling Technology, Cat# 4835), Rabbit monoclonal anti-HIF2α (Cell Signaling Technology, Cat# 7096), Rabbit polyclonal anti-TOM20 (Cell Signaling Technology, Cat# 13929), Rabbit polyclonal anti-TFAM (Abcam, Cat# ab131607), Mouse monoclonal anti-GAPDH (Abcam, Cat# ab8245), Mouse monoclonal anti-OXPHOS (Abcam Cat# ab110413), Mouse monoclonal anti-MT-CO1 (Abcam Cat# ab14705), Rabbit polyclonal anti-MT-CO2 (Abcam Cat# ab91317), Rabbit polyclonal anti-MT-ND1 (Abcam Cat# ab181848), Rabbit polyclonal anti-MT-ATP6 (Abcam Cat# ab192423), Rabbit polyclonal anti-MT-CYB (Abcam Cat# ab81215), Rabbit polyclonal anti-LONP1 (Abcam Cat# ab103809), Rabbit polyclonal anti-Tyrosine Hydroxylase (Abcam Cat# ab112), Rabbit polyclonal anti-Hydroxyproline (Abcam, Cat# ab37067,Lot: GR3215743-1 GR3179915-1), Rabbit monoclonal anti-

Cyclin D1 (Abcam Cat# ab134175), Rabbit polyclonal anti-HIF1α (Novus Biologicals,Cat# NB100-479), Rabbit polyclonal anti-HIF2α (Novus Biologicals, Cat# NB100-122), Mouse monoclonal anti-alpha-Tubulin (Sigma-Aldrich, Cat# T5168), Mouse monoclonal anti-HA (Sigma-Aldrich, Cat# H9658), Rabbit polyclonal anti-Flag (Sigma-Aldrich, Cat# F7425), Mouse monoclonal anti-PGC1α (Millipore, Cat# ST1202), Mouse monoclonal anti-VHL (BD Biosciences, Cat# 556347), Mouse monoclonal anti-VHL (BD Biosciences, Cat# 564183), Rabbit polyclonal anti-EGLN2 (Affinity Biosciences, Cat# DF7918), Mouse monoclonal anti-TUJ1 (Covance, Cat# MMS-435P), Mouse monoclonal anti-c-Myc (Thermo Fisher Scientific, Cat# 13-2500), Mouse monoclonal anti-p-Ser (16B4) (Santa Cruz Biotechnology, Cat# sc-81514).

Validation

None of the antibodies in this study are novel or uniques. All antibodies used in this study are commercially available and have been validated in previously published studies.
Rabbit monoclonal anti-TFAM (Cell Signaling Technology, Cat# 8076)
https://www.cellsignal.com/products/primary-antibodies/tfam-d5c8-rabbit-mab/8076

Rabbit polyclonal anti-PKA C-α (Cell Signaling Technology, Cat# 4782)
https://www.cellsignal.com/products/primary-antibodies/pka-c-a-antibody/4782

Rabbit monoclonal anti-PHD-2/Egln1 (Cell Signaling Technology, Cat# 4835)
https://www.cellsignal.com/products/primary-antibodies/phd-2-egln1-d31e11-rabbit-mab/4835

Rabbit monoclonal anti-HIF2α (Cell Signaling Technology, Cat# 7096)
https://www.cellsignal.com/products/primary-antibodies/hif-2a-d9e3-rabbit-mab/7096

Rabbit polyclonal anti-TOM20 (Cell Signaling Technology, Cat# 13929)
https://www.cellsignal.com/product/productDetail.jsp?productId=13929

Rabbit polyclonal anti-TFAM (Abcam, Cat# ab131607)
https://www.abcam.cn/mttfa-antibody-mitochondrial-marker-ab131607.html

Mouse monoclonal anti-GAPDH (Abcam, Cat# ab8245)
https://www.abcam.cn/gapdh-antibody-6c5-loading-control-ab8245.html

Mouse monoclonal anti-OXPHOS (Abcam Cat# ab110413)
https://www.abcam.cn/total-oxphos-rodent-wb-antibody-cocktail-ab110413.html

Mouse monoclonal anti-MT-CO1 (Abcam Cat# ab14705)
https://www.abcam.cn/mtco1-antibody-1d6e1a8-ab14705.html

Rabbit polyclonal anti-MT-CO2 (Abcam Cat# ab91317)
https://www.abcam.cn/mtco2-antibody-ab91317.html

Rabbit polyclonal anti-MT-ND1 (Abcam Cat# ab181848)
https://www.abcam.cn/mt-nd1-antibody-epr134662-ab181848.html

Rabbit polyclonal anti-MT-ATP6 (Abcam Cat# ab192423)
https://www.abcam.cn/mt-atp6-antibody-ab192423.html

Rabbit polyclonal anti-MT-CYB (Abcam Cat# ab81215)
https://www.abcam.cn/mt-cyb-antibody-ab81215.html

Rabbit polyclonal anti-LONP1 (Abcam Cat# ab103809)
https://www.abcam.cn/lonp1lon-antibody-ab103809.html

Rabbit polyclonal anti-Tyrosine Hydroxylase (Abcam Cat# ab112)
https://www.abcam.cn/tyrosine-hydroxylase-antibody-neuronal-marker-ab112.html

Rabbit polyclonal anti-Hydroxyproline (Abcam, Cat# ab37067,Lot: GR3215743-1 GR3179915-1)
https://www.abcam.cn/hydroxyproline-antibody-ab37067.html

Rabbit monoclonal anti- Cyclin D1 (Abcam Cat# ab134175)
https://www.abcam.cn/cyclin-d1-antibody-epr2241-c-terminal-ab134175.html

Rabbit polyclonal anti-HIF1α (Novus Biologicals,Cat# NB100-479)
https://www.novusbio.com/products/hif-1-alpha-antibody_nb100-479

Rabbit polyclonal anti-HIF2α (Novus Biologicals, Cat# NB100-122)
https://www.novusbio.com/products/hif-2-alpha-epas1-antibody_nb100-122

Mouse monoclonal anti-alpha-Tubulin (Sigma-Aldrich, Cat# T5168)
https://www.sigmaaldrich.cn/CN/zh/product/sigma/t5168

Mouse monoclonal anti-HA (Sigma-Aldrich, Cat# H9658)
https://www.sigmaaldrich.cn/CN/zh/product/sigma/h9658

Rabbit polyclonal anti-Flag (Sigma-Aldrich, Cat# F7425)
https://www.sigmaaldrich.cn/CN/zh/product/sigma/f7425

Mouse monoclonal anti-PGC1α (Millipore, Cat# ST1202)
https://www.merckmillipore.com/CN/zh/product/Anti-PGC-1-Mouse-mAb-4C1.3,EMD_BIO-ST1202

Mouse monoclonal anti-VHL (BD Biosciences, Cat# 556347)
https://www.bdbiosciences.com/en-us/products/reagents/microscopy-imaging-reagents/immunohistochemistry-reagents/purified-mouse-anti-vhl.556347

Mouse monoclonal anti-VHL (BD Biosciences, Cat# 564183)
https://www.bdbiosciences.com/en-us/products/reagents/flow-cytometry-reagents/research-reagents/single-color-antibodies-ruo/purified-mouse-anti-human-vhl.564183

Rabbit polyclonal anti-EGLN2 (Affinity Biosciences, Cat# DF7918)
http://www.affbiotech.com/goods-11601-DF7918-EGLN2_Antibody.html

Mouse monoclonal anti-TUJ1 (Covance, Cat# MMS-435P)
https://elifesciences.org/articles/70518

Mouse monoclonal anti-c-Myc (Thermo Fisher Scientific, Cat# 13-2500)
https://www.thermofisher.cn/cn/zh/antibody/product/c-Myc-Antibody-clone-9E10-Monoclonal/13-2500

Mouse monoclonal anti-p-Ser (16B4) (Santa Cruz Biotechnology, Cat# sc-81514)
https://www.scbt.com/p/p-ser-antibody-16b4?requestFrom=search

Eukaryotic cell lines

Policy information about cell lines

Cell line source(s)	Human renal cell carcinoma 786-O (CRL-1932) and A498 (HTB-44), HeLa (ATCC CCL-2) and rat pheochromocytoma cell line PC12 (CRL-1721) were purchased from ATCC. 293FT Cell Line ThermoFisher (Catalog number: R70007)<br>Primary mouse embryotic fibroblast (MEFs) were isolated from EglN3 wild-type and knockout mice.
Authentication	Cell lines were authenticated by short tandem repeats (STR) testing.
Mycoplasma contamination	Cells were tested negative for mycoplasma contamination.
Commonly misidentified lines<br>(See ICLAC register)	No misidentified cell lines were used.

Animals and other organisms

Policy information about studies involving animals; ARRIVE guidelines recommended for reporting animal research

Laboratory animals	Immunoblot experiments were performed using postnatal day1, day7 (female), 10-11, 16, 40 (female), 80 weeks old EglN3 WT and KO C57BL/6 mice.<br>Graded treadmill running test experiments were performed using 18-19 weeks and 56-60 weeks old EglN3 WT and KO C57BL/6 male mice. Mouse tumor xenograft experiment was performed using 6 – 8 weeks old male CB17/Icr-Prkdcscid/Rj mice purchased from Janvier Labs (France).
Wild animals	None
Field-collected samples	None
Ethics oversight	Ethical permits for animal studies were approved by the appropriate local and national authorities – Jordbruksverket, Sweden. Animal experiments were performed in accordance with Swedish animal welfare laws authorized by the Stockholm Animal Ethics Committee (Dnr 7694/17).<br>All treadmill running experiments were approved by the regional animal ethics committee of Northern Stockholm, Sweden (#4039-2018 and #4359-2020).<br>Mouse tumor xenograft models were approved by the Swedish Board of Agriculture (ethical number 6197-2019).

Note that full information on the approval of the study protocol must also be provided in the manuscript.

Human research participants

Policy information about studies involving human research participants

Population characteristics	VHL mutations in cases 21, 25, 96 and 108 as well as WT VHL status for the other 6 cases have been previously described (PMID: 24694336).<br>Case 21 was diagnosed at 31 years of age with cerebellar HB and PCC the same year. The patient has type 1 VHL syndrome with a germline VHL non-sense mutation Q73X (c.217C>T p.Gln73X). The mutation is a stop mutation resulting in truncated pVHL , i.e. type 1 VHL. The risk of developing PCC for patients with type 1 VHL is not zero, but lower than for type 2 VHL.<br>Case 25 was diagnosed with PCC at the age of 13 with normal epinephrine and norepinephrine levels with no evidence of metastasis or relapse and no additional tumors. The patient has type 2 VHL syndrome and carries a germline VHL missense mutation S65A (c.193T>G p.Ser65Ala).<br>Case 96 was diagnosed at age 47 with PCC and with trichoepithelioma eight years earlier (benign skin lesion). The patient is an apparently sporadic case (no VHL syndrome) with a somatic VHL missense mutation L129P (c.386T>C p.Leu129Pro). Constitutional mutations were revealed in EGLN1 (c.799G>A p.Glu267Lys) and SDHA (c.223C>T p.Arg75X)<br>Case 108 was diagnosed with abdominal PGL at 65 years of age, followed by basal cell carcinoma of the skin 15 years later and follicular lymphoma 20 years later. The patient is an apparently sporadic case (no VHL syndrome) with a somatic VHL missense mutation L198R (c.593T>G p.Leu198Arg).
Recruitment	PPGL tissue samples (9 PCC and 1 abdominal PGL) were collected from patients operated and diagnosed at the Karolinska University Hospital, Stockholm, Sweden, previously characterized for mutations in 14 proposed PPGL susceptibility genes. All samples were obtained with informed patient consent and with approval from the local ethical committees.
Ethics oversight	Collection and analyses of human samples (normal adrenal tissues, PCCs and PGLs) are covered by the ethical approvals Dnr 01-136, KI forskningsetikkommitté Nord and Dnr 2020-04226.

Note that full information on the approval of the study protocol must also be provided in the manuscript.

Flow Cytometry

Plots

Confirm that:

- [x] The axis labels state the marker and fluorochrome used (e.g. CD4-FITC).
- [x] The axis scales are clearly visible. Include numbers along axes only for bottom left plot of group (a 'group' is an analysis of identical markers).
- [x] All plots are contour plots with outliers or pseudocolor plots.
- [x] A numerical value for number of cells or percentage (with statistics) is provided.

Methodology

Sample preparation	Cells were stained with MitoTracker® Green FM (100 nM) at 37 °C for 30 min for labelling mitochondria.
Instrument	LSRFortessa flow cytometer (BD Biosciences)
Software	Samples were acquired using BD FACS Diva, and analysed using FlowJo version 10
Cell population abundance	No sorted cells were implicated in this study.
Gating strategy	Cells were gated by FSC-A vs SSC-A to exclude low size particles, then cells were further gated by FSC-A vs FSC-H to discriminate single cells. Fluorescence intensity was analyzed within single cells.

- [x] Tick this box to confirm that a figure exemplifying the gating strategy is provided in the Supplementary Information.
